# DFT Mechanistic Investigation into Ni(II)-Catalyzed Hydroxylation of Benzene to Phenol by H_2_O_2_

**DOI:** 10.1021/acs.inorgchem.3c04461

**Published:** 2024-03-12

**Authors:** Kaveh Farshadfar, Kari Laasonen

**Affiliations:** Department of Chemistry and Material Science, School of Chemical Engineering, Aalto University, 02150 Espoo, Finland

## Abstract

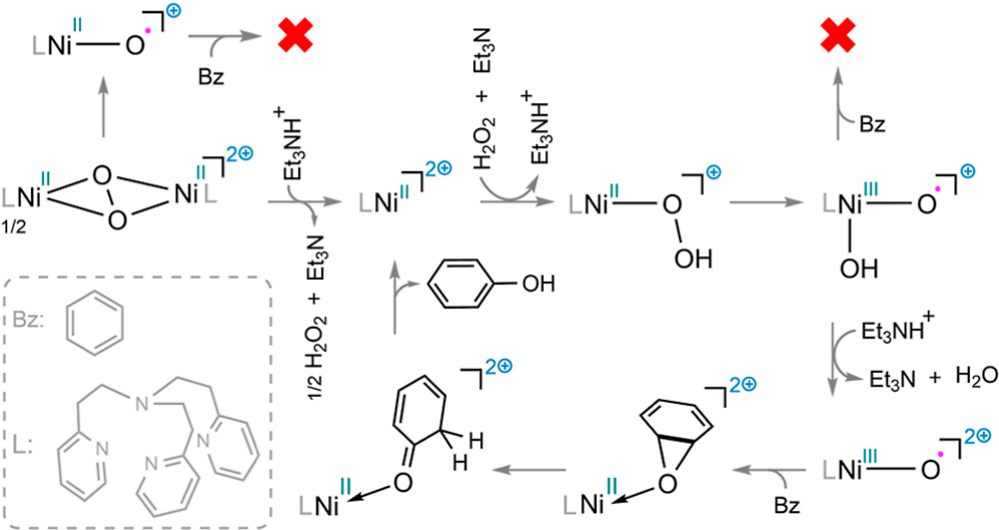

Introduction of oxygen into aromatic C–H bonds is intriguing from both fundamental and practical perspectives. Although the 3d metal-catalyzed hydroxylation of arenes by H_2_O_2_ has been developed by several prominent researchers, a definitive mechanism for these crucial transformations remains elusive. Herein, density functional theory calculations were used to shed light on the mechanism of the established hydroxylation reaction of benzene with H_2_O_2_, catalyzed by [Ni^II^(tepa)]^2+^ (tepa = tris[2-(pyridin-2-yl)ethyl]amine). Dinickel(III) bis(μ-oxo) species have been proposed as the key intermediate responsible for the benzene hydroxylation reaction. Our findings indicate that while the dinickel dioxygen species can be generated as a stable structure, it cannot serve as an active catalyst in this transformation. The calculations allowed us to unveil an unprecedented mechanism composed of six main steps as follows: (i) deprotonation of coordinated H_2_O_2_, (ii) oxidative addition, (iii) water elimination, (iv) benzene addition, (v) ketone generation, and (vi) tautomerization and regeneration of the active catalyst. Addition of benzene to oxygen, which occurs via a radical mechanism, turns out to be the rate-determining step in the overall reaction. This study demonstrates the critical role of Ni-oxyl species in such transformations, highlighting how the unpaired spin density value on oxygen and positive charges on the Ni–O^•^ complex affect the activation barrier for benzene addition.

## Abstract 2

During the Ni(II)-catalyzed hydroxylation process of benzene to phenol by H_2_O_2_, although dinickel dioxygen species can exist as stable structures, they cannot function as an active catalyst. DFT calculations reveal that the rate-determining step in this transformation is the addition of benzene to oxygen, which necessitates Ni–O species with high oxyl character and a significant positive charge.

## Introduction

Phenol and its derivatives are a valuable class of chemical precursors used in the synthesis of various industrial products, including dyes, phenol-formaldehyde resins, bisphenol A, caprolactam, pharmaceuticals, medicines, polymers, and raw materials for numerous chemicals.^1^ The current industrial process for phenol production relies on three-step cumene methods, which involve the propylation of benzene or toluene, autoxidation to obtain the cumene hydroperoxide derivative, and Hock rearrangement. These reactions require a high temperature, high pressure, and strongly acidic conditions, resulting in the production of acetone as a byproduct in equal quantities during the final step. Furthermore, the overall yield of phenol from benzene is disappointingly low, reaching only 5%.^2,3^ Therefore, the industry desires a simpler and more efficient process that can be conducted under milder conditions. In particular, there has been considerable research focused on developing a direct aromatic oxygenation reaction using cost-effective and environmentally benign oxidants such as O_2_ and H_2_O_2_.^4−19^ Even so, introducing hydroxyl functionality into arenes using these oxidants presents challenges due to the modest reactivity of aromatic C–H bonds and the tendency for phenols to overoxidize, ultimately resulting in a diminished reaction efficiency.^20−22^

**Scheme 1:**
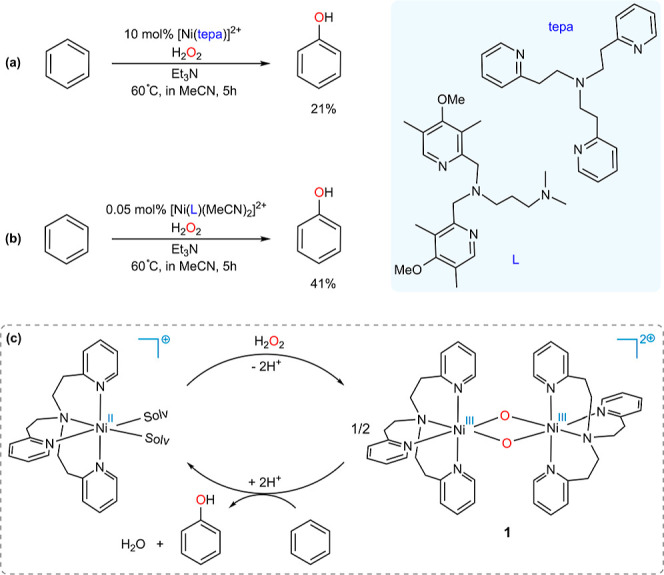
Nickel-Catalyzed Hydroxylation of Benzene Using H_2_O_2_ Developed by (a) Itoh et al. and (b) Mayilmurugan et al. (c) Proposed Catalytic Mechanism by Itoh and Co-workers

In 2015, Itoh and co-workers^14^ reported a remarkable study in which they described the catalytic ability of nickel(II) complexes supported by pyridylalkylamine ligands as homogeneous catalysts for the direct hydroxylation of benzene to phenol using H_2_O_2_ as the oxidant (Scheme 1a). The same approach was executed by Mayilmurugan and co-workers employing similar ligands (Scheme 1b).^10^ Both research groups suggested that a dinickel(III) bis(μ-oxo) species serves as an active catalyst in the hydroxylation process of benzene and its derivatives (Scheme 1c).

Although Itoh and other researchers have demonstrated that dinickel(III) bis(μ-oxo) complexes can form under such conditions,^23−26^ there is no experimental or computational support for their involvement in the hydroxylation of benzene. It is noteworthy that while a few instances of intramolecular oxidation involving aromatic groups embedded within the supporting ligands of dinickel(III) bis(μ-oxo) species have been reported,^23,27,28^ there are no known cases of intermolecular oxidation of benzene derivatives occurring through such a species. Moreover, in 2018, Itoh and co-workers reported the synthesis of the [Ni^III^-(dpema)_2_(O)_2_]^2+^ complex (dpema:*N*,*N*-(di-[2-pyridine-2-yl]ethyl)methylamine), which exhibited oxygenation reactivity toward external substrates but not aromatic oxidation.^29^

This outstanding oxidation system prompted us to conduct a comprehensive density functional theory (DFT) study to investigate the mechanistic aspects of benzene hydroxylation utilizing H_2_O_2_ as the oxidant and this particular type of Ni(II) complex as a catalyst. Elucidating the mechanism underlying this hydroxylation reaction can enhance our knowledge of the processes involved in other transition metal-catalyzed hydroxylation reactions of aromatic substrates using H_2_O_2_.^4,5,7,9,11,12,30−37^ To do so, we selected the [Ni^II^(tepa)]^2+^ complex based on the study by Itoh et al., which has demonstrated its superior efficiency over other analogous nickel complexes supported by pyridylalkylamine ligands.^14^

## Results and Discussion

### Preliminary Evaluation of the Mechanism Proposed by Itoh et al.

As discussed in the Introduction, the dinickel(III) bis(μ-oxo) complex has been surmised to be the key intermediate responsible for the catalytic hydroxylation of benzene (Scheme 1c). This species can be produced when hydrogen peroxide is added to the two molecules of [Ni^II^(tepa)]^2+^. Hence, we commenced our mechanistic inquiry by assessing the capability of **1** to oxidize benzene. It should be pointed out that side-on dioxygen 3d metal complexes commonly adopt either a bis(μ-oxo)^24,26,29,38−43^ or μ–η^2^:η^2^-peroxo isomeric form.^44−47^ In the present case, the μ–η^2^:η^2^-peroxo form (^**s**^**2**), which is an antiferromagnetic substance with two spin-up electrons on one nickel atom and two spin-down electrons on the other nickel atom, was found to exhibit greater stability than that of the bis(μ-oxo) form (**1**) (see Supporting Information for details).

**Figure 1:**
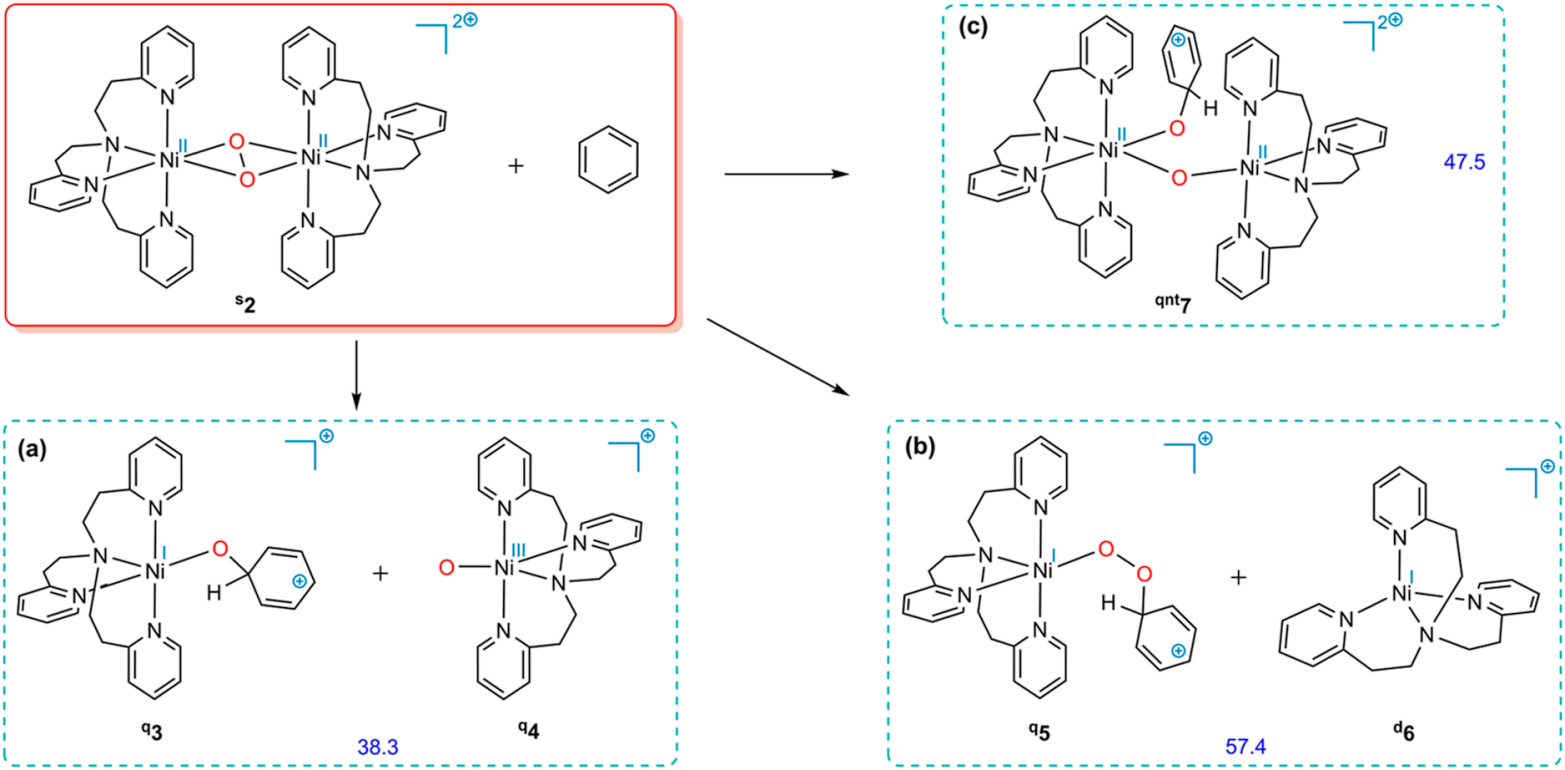
DFT calculated reaction potential pathways at the SMD/B3LYP-D3/def2-TZVP//SMD/B3LYP-D3/6-31G(d),SDD level of theory for the (a) and (b) substitution reactions between benzene and ^**s**^**2** and (c) addition of benzene to ^**s**^**2**. The most stable ground state for each structure has been taken into account to calculate the free energies of the reactions. The superscripts “s”, “d”, “q”, and “qnt” represent the singlet, doublet, quartet, and quintet ground states, respectively. Free energies are given in kcal/mol (in blue).

As depicted in Figure 1a,^48^ the substitution reaction between benzene and ^**s**^**2**, yielding (tepa)Ni–O–Bz (Bz = benzene) and (tepa)Ni–O species, exhibits a pronounced thermodynamic unfavorability, with a Δ*G*_rxn_ = 36.8 kcal/mol, rendering it infeasible. Similarly, an alternate pathway for the substitution reaction leading to the production of (tepa)Ni–O–O-Bz and (tepa)Ni is extremely endothermic at Δ*G*_rxn_ = 57.4 kcal/mol (Figure 1b). Also, the addition of benzene to oxygen without the full cleavage of ^**s**^**2** is highly unfavorable by Δ*G*_rxn_ = 47.5 kcal/mol, suggesting that it is unlikely (Figure 1c). On the basis of these results, it can be deduced that complex ^**s**^**2** is inactive toward the oxidation of benzene due to the energetically unfavorable nature of the substitution reactions involving benzene.

### Other Potential Pathways for Benzene Hydroxylation Starting from [Ni^II^(tepa)]^2+^

**Figure 2:**
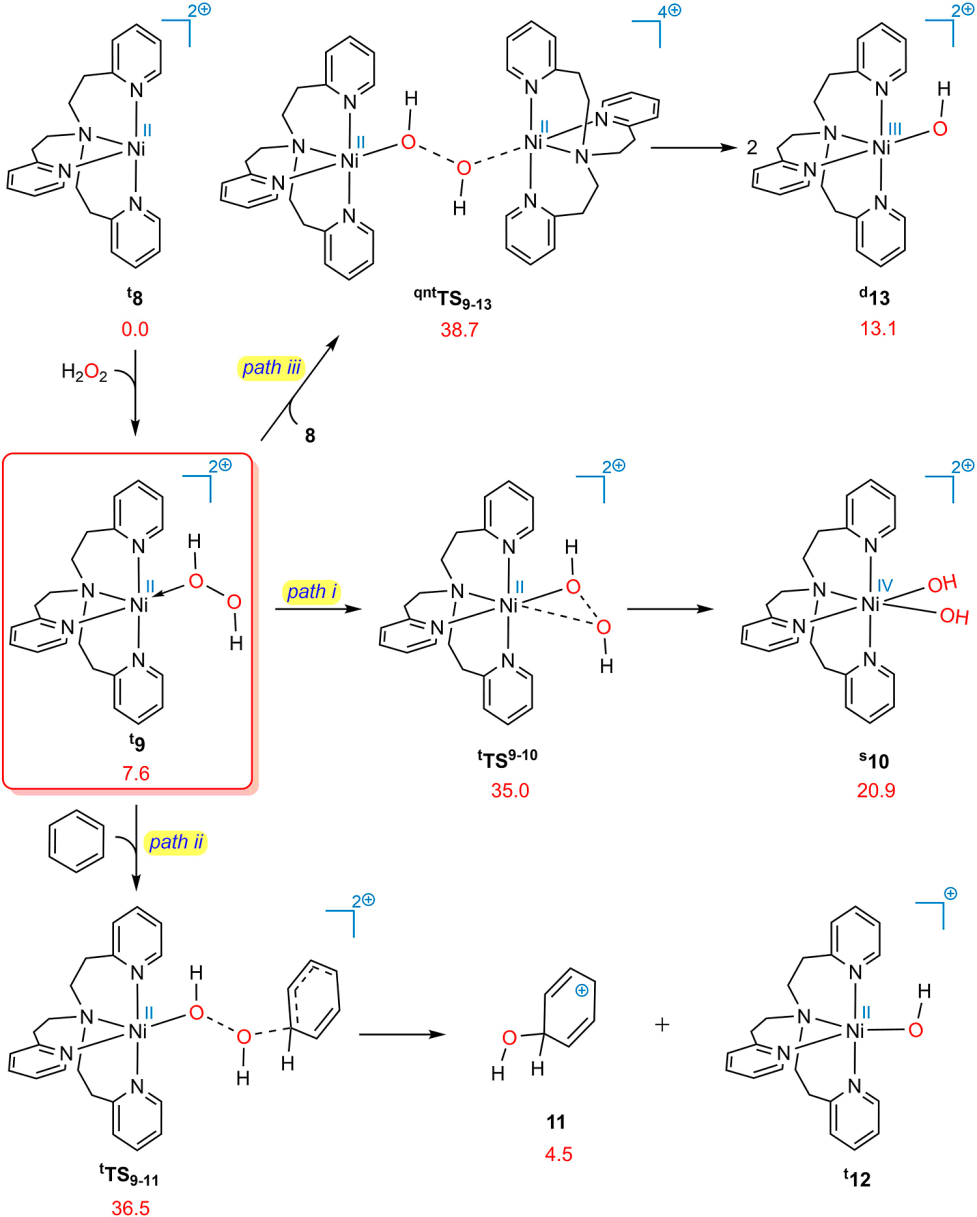
Calculated reaction pathways for oxidative addition of H_2_O_2_ to [Ni^II^(tepa)]^2+^ (path i); nucleophilic attack on coordinated H_2_O_2_ by benzene (path ii) and [Ni^II^(tepa)]^2+^ (path iii). The superscripts “s”, “d”, “t”, and “qnt” represent the singlet, doublet, triplet, and quintet ground states, respectively. Free energies calculated at the SMD/B3LYP-D3/def2-TZVP//SMD/B3LYP-D3/6-31G(d),SDD level of theory are given in kcal/mol (in red).

We then extended our investigation by exploring potential mechanistic pathways underlying benzene hydroxylation, which are discussed in the following. [Ni^II^(tepa)]^2+^ (^**t**^**8**), an octahedral complex with two vacant coordination sites, is determined to have a slightly lower free energy than those with one or two coordinated molecules of acetonitrile as the solvent (see Supporting Information for details). Here, commencing from ^**t**^**8**, we examine potential pathways for benzene hydroxylation:

A Oxidative addition of H_2_O_2_ to [Ni^II^(tepa)]^2+^: The Ni(IV) species is expected to exhibit a higher oxidation potential for driving benzene hydroxylation compared to that of Ni(II). However, to proceed with the oxidative addition of H_2_O_2_ to the Ni center, an activation energy barrier of 35.0 kcal/mol must be overcome, which is inaccessible (path i in Figure 2).
B Nucleophilic attack on coordinated H_2_O_2_: The nucleophilic attack of benzene on coordinated H_2_O_2_ in ^**t**^**9** is concerted with the breaking of the O–O bond, leading to the formation of carbocation (**11**) alongside Ni(II) species (^**t**^**12**). The generated carbocation, due to its high acidity, would readily undergo proton donation to convert into phenol. Nevertheless, the calculations demonstrate that this pathway is impeded by a 36.5 kcal/mol activation energy barrier (path ii in Figure 2).

We also examined an alternative route in which ^**t**^**8** undergoes nucleophilic attack on coordinated H_2_O_2_ instead of benzene (path iii in Figure 2). Nonetheless, the transition structure for this transformation lies 38.7 kcal/mol above the reactants. It follows that this activation barrier is high enough to hinder the progression of the reaction.

### Catalytic Process Uncovered by DFT Calculations

#### Generation of Oxyl

In the wake of the aforementioned results, we have once again been compelled to seek a different route for this process. Triethylamine (Et_3_N) is added to the reaction mixture during the hydroxylation of benzene and its derivatives.^14^ According to our calculations, when complex ^**t**^**9** is treated with Et_3_N, it can undergo deprotonation in a barrierless manner, resulting in the production of ^**t**^**14** with a relative free energy of −1.2 kcal/mol. Indeed, the coordination of H_2_O_2_ with Ni(II) greatly enhances its acidity to the extent that it can readily donate a proton to a weak base such as Et_3_N. The resulting complex (^**t**^**14**) is now more prone to the oxidative addition of the OOH^–^ ligand, with a relative free energy barrier of 22.7 [21.5 – (−1.2)] kcal/mol.

The greater tendency of ^**t**^**14** toward oxidative addition (Δ*G*^*⧧*^ = 22.7) relative to ^**t**^**9** (Δ*G*^*⧧*^ = 27.4) can be attributed to the following factors. (1) ^**t**^**14** carries less positive charge than ^**t**^**9**, resulting in an elevated electron density at the Ni center, as confirmed by natural population analysis (NPA), which indicated an increase of 0.124 units. This heightened electron density at the Ni center renders ^**t**^**14** more prone to oxidation. (2) During the oxidative addition, two electrons are transferred from the d orbitals of Ni to the σ^★^(O–O) orbital, which cleaves the O–O bond. Moving toward a more electron-rich Ni center somewhat diminishes the effective nuclear charge experienced by electrons in the d shell, which, in turn, increases their energy levels. As a result, transferring electrons to the σ^★^(O–O) orbital in ^**t**^**14** is more facilitated than that in ^**t**^**9**. (3) There is an intrinsic instability associated with Ni(IV) species. In ^**s**^**10**, the nickel center is confined to the +4 oxidation state, whereas in ^**t**^**15**, nickel can adapt to the +3 oxidation state by accepting one electron from oxygen, resulting in the formation of the oxyl species. This will make the generation of ^**t**^**15** more favorable than that of ^**s**^**10**.

**Figure 3:**
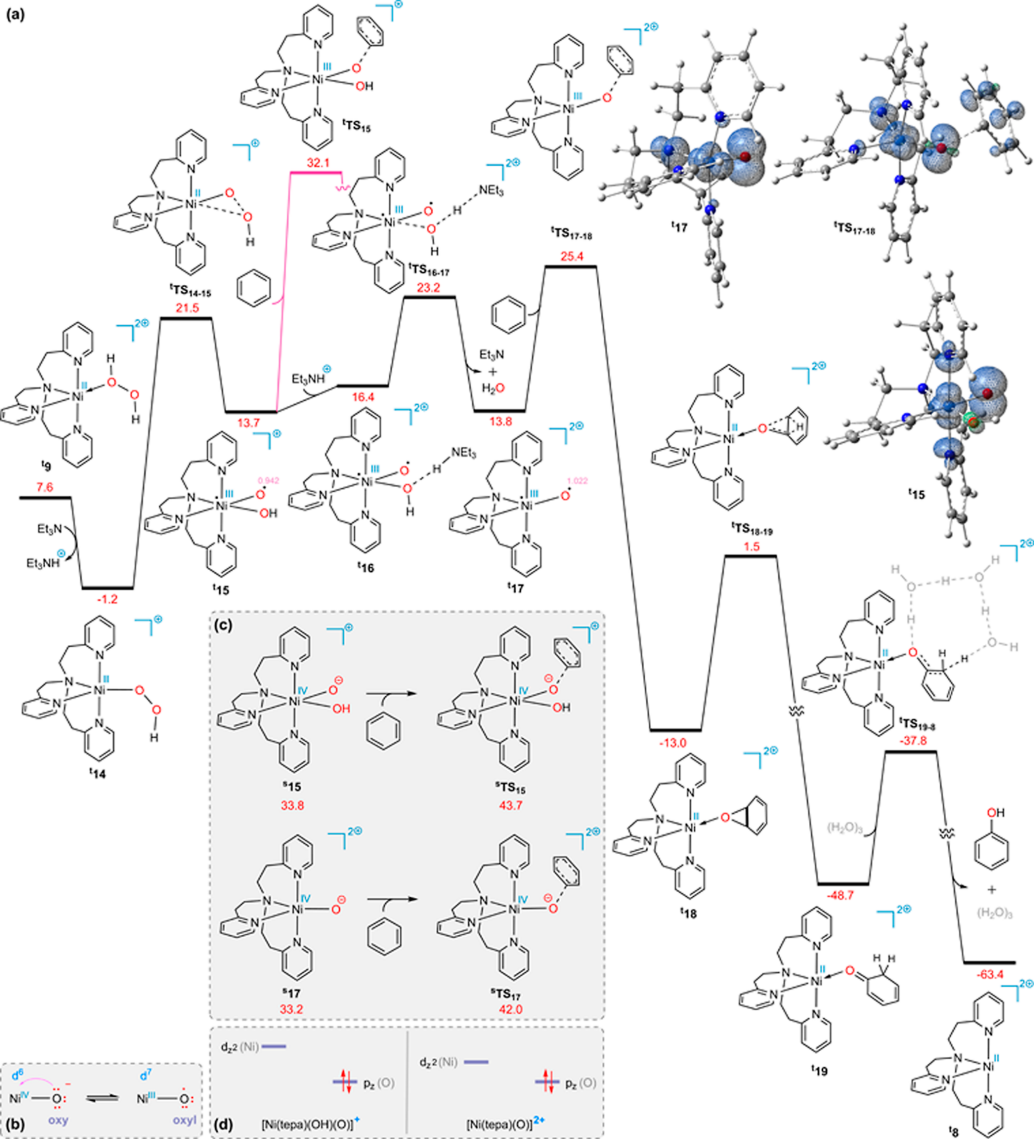
(a) Calculated energy profile for [Ni^II^(tepa)]^2+^-catalyzed hydroxylation of benzene; spin density distribution plots for ^**t**^**15**, ^**t**^**17**, and ^**t**^**TS**_**17**–**18**_ at an isosurface value of 0.006. (b) Equilibria between oxy and oxyl forms. (c) Calculated energy barrier for the nucleophilic attack of benzene to the oxy group of ^**s**^**15** and ^**s**^**17**. (d) Schematic illustration of the effect of the formal charge of the nickel complex on the ease of conversion from oxy to oxyl. The superscripts “s” and “t” represent the singlet and triplet ground states, respectively. The relative Gibbs free energy values obtained from the SMD/B3LYP-D3/def2-TZVP//SMD/B3LYP-D3/6-31G(d),SDD calculations are given in kcal/mol (in red) and spin density values in e/Å^3^ (in pink).

The ensuing nickel complex (^**t**^**15**) may undergo benzene attack at its oxygen atom. Considering two metal-oxy and metal-oxyl structures,^49^ there are two distinct pathways through which benzene can attack Ni–O (Figure 3b): nucleophilic attack on the oxy group, which occurs via the singlet ground state ^**s**^**TS**_**15**_, or a radical mechanism targeting the oxyl group through the triplet ground state ^**t**^**TS**_**15**_. DFT calculations indicate a substantial energy barrier of 44.9 [43.7 – (−1.2)] kcal/mol for the nucleophilic attack mechanism [Figure 3c (top)]. Although the radical mechanism exhibits a comparatively lower activation energy of 33.3 [32.1 – (−1.2)] kcal/mol, it is still fairly high. It is, however, possible for Et_3_NH^+^ to, after forming a hydrogen-bonded adduct (^**t**^**16**), protonate the hydroxide ligand via the transition state ^**t**^**TS**_**16**–**17**_, leading to the elimination of water and the production of intermediate ^**t**^**17**. The ensuing complex is prone to undergo benzene attack via the radical mechanism (^**t**^**TS**_**17**–**18**_) as the key step of this transformation (Δ*G*^*⧧*^ = 26.6 [25.4 – (−1.2)] kcal/mol). It is noteworthy that, similar to ^**s**^**15**, the nucleophilic attack of benzene on the oxy group at ^**s**^**17**, which should be performed at the singlet ground state (^**s**^**TS**_**17**_), is hindered by a high activation energy barrier of 43.2 [42.0 – (−1.2)] kcal/mol. [Figure 3c (bottom)]. It is notable that species ^**t**^**8**, ^**t**^**14**, ^**t**^**15**, and ^**t**^**17** and transition structures ^**t**^**TS**_**15**_ and ^**t**^**TS**_**17**–**18**_ exhibit squared total spin angular momentum operator ⟨*S*^2^⟩ values of 2.006, 2.006, 2.020, 2.012, 2.088, and 2.093, respectively, based on our calculations. These values indicate that they bear minimal spin contamination.

### Why Does [Ni^II^(tepa)(O)]^2+^ Have a Greater Tendency than That of [Ni^II^(tepa)(O)(OH)]^+^ toward Addition of Benzene through the Radical Mechanism?

Investigating why the free energy barriers differ for ^**t**^**TS**_**15**_ (Δ*G*^*⧧*^ = 33.1 kcal/mol) and ^**t**^**TS**_**17**–**18**_ (Δ*G*^*⧧*^ = 26.6 kcal/mol) can provide insight into these processes. ^**t**^**15** and ^**t**^**17** possess respective spin density values of 0.942 and +1.022 e/Å^3^ on oxygen, respectively (noted in pink in Figure 3a). Certainly, the spin density is a decisive parameter in the radical mechanism. This difference in spin densities on oxygen can be rationalized on the basis of the orbital energies. As mentioned above, two oxy and oxyl forms of Ni–O can coexist, and only the oxyl form, which is a diradical, can react with benzene via the radical mechanism. The oxyl form is the result of transferring an electron from the occupied oxygen p_*z*_ orbital to the unoccupied nickel d_*z*_^2^ orbital (Figure 3b). Thus, the energy level of the d_*z*_^2^ orbital significantly influences the contribution of the oxyl structure. ^**t**^**17**, which carries more positive charges than ^**t**^**15**, has lower orbital energy levels for nickel. Consequently, the diminished energy level for the vacant d_*z*_^2^ orbital facilitates O(p_*z*_) → Ni(d_*z*_^2^) electron transfer, resulting in a greater contribution of the oxyl form (Figure 3d). Therefore, ^**t**^**17** is more likely to participate in the radical mechanism.

### Formation of the Second C–O Bond

**Figure 4:**
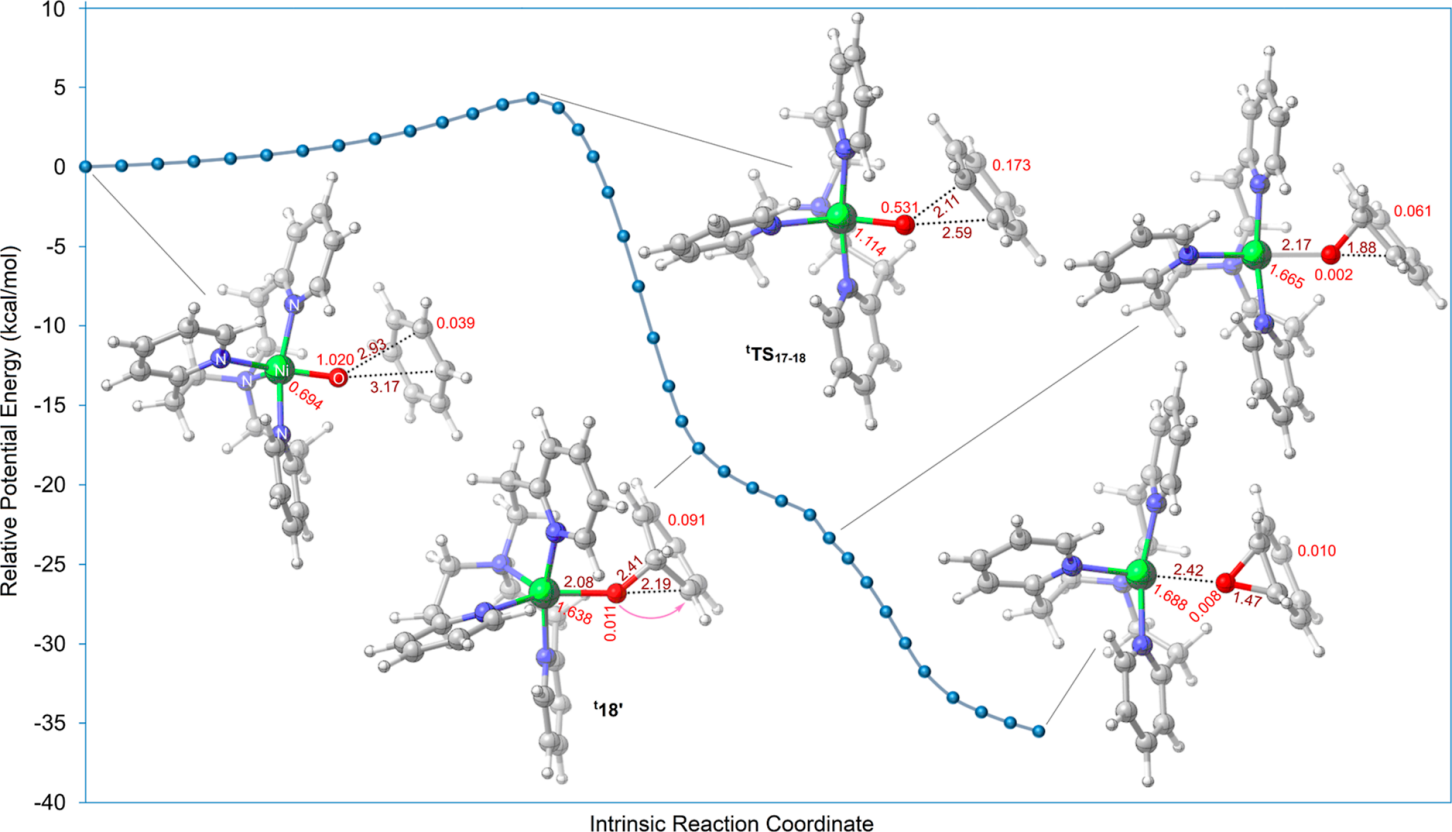
Plot of SMD/B3LYP-D3/6-31G(d),SDD energies for points along the IRC of ^**t**^**TS**_**17**–**18**_. The superscript “t” represents the triplet ground state. Interatomic distances are shown in Å (in brown) and spin density values for Ni atoms, O atoms, and benzene components for selected structures along the IRC path in e/Å^3^ (in red).

Intrinsic reaction coordinate (IRC) calculations at the B3LYP-D3/6-31G(d),SDD level of theory (Figure 4) show that the transition structure ^**t**^**TS**_**17**–**18**_ connects ^**t**^**17** to ^**t**^**18**, passing through a nonlocal minimum structure ^**t**^**18**′, where a C–O bond has been formed. Subsequently, in the zwitterionic benzene-O component in ^**t**^**18**′, oxygen nucleophilically attacks the generated carbocation to form a new C–O bond, furnishing ^**t**^**18** with a relative free energy of −13.0 kcal/mol (Figure 3a).

Features of this step were also sought in terms of the spin density distribution on the nickel, oxygen, and benzene components (Figure 4). The Mulliken spin density analysis indicates that initially, the two single electrons are predominantly located on the nickel and oxygen atoms. On approaching ^**t**^**TS**_**17**–**18**_, spin density on oxygen decreases and that on nickel increases. The changes in Mulliken spin densities indicate that as the unpaired electron on oxygen pairs with one π electron from the benzene ring, the remaining unpaired electron in benzene is transferred to the nickel center.

### Generation of the Ketone

The conversion of benzene oxide to phenol has been documented in various catalytic systems.^13,50−54^ Here, we describe a pathway in which nickel complex ^**t**^**8** plays a catalytic role in the conversion of phenol from benzene oxide as well. Intermediate ^**t**^**18** is reactive toward ring opening (breaking of the O–C bond) of its epoxide moiety via transition structure ^**t**^**TS**_**18**–**19**_ by overcoming an energy barrier of 14.5 kcal/mol. A hydride shift occurs immediately after ring opening to give intermediate ^**t**^**19** in a highly exothermic fashion (Figure 3a). Indeed, DFT results suggest that ^**t**^**TS**_**18**–**19**_ is directly connected to ^**t**^**19**, and this coordinated ketone to the nickel center is the only stationary point on the potential energy surface beyond this transition structure.

### Tautomerization to Phenol and Regeneration of the Active Catalyst

Coordination of the ketone to the Ni(II) in ^**t**^**19** causes the β-hydrogen to become remarkably acidic,^55,56^ leading to its facile deprotonation by Et_3_N or H_2_O. Due to the significantly higher concentration of water compared to that of Et_3_N, water was used as the proton shuttle in this context. A cluster of three water molecules (H_2_O)_3_^57−59^ then drives this keto–enol tautomerization via ^**t**^**TS**_**19**–**8**_, yielding the phenol product and regenerating the active catalyst ^**t**^**8** (Figure 3a).

### Production of the Dinickel Dioxygen Complex

**Figure 5:**
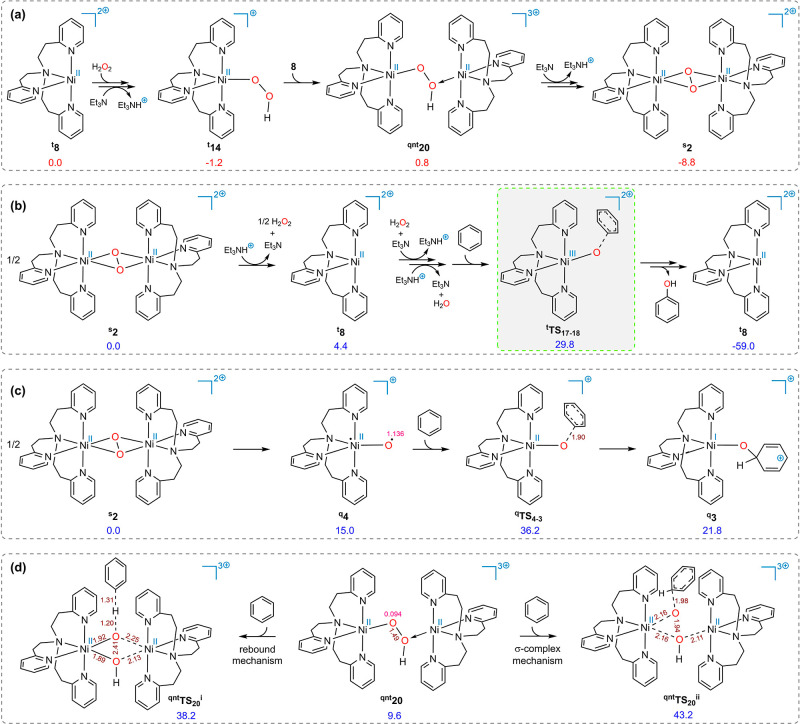
(a) Calculated pathway for the formation of ^**s**^**2**. (b) Calculated relative free energy for the key transition structures (^**t**^**TS**_**17**–**18**_) and the final product starting from ^**s**^**2**. (c) Calculated free energies of reactions between ^**q**^4 and benzene. (d) Calculated free energies for the transition structures of the oxygen-rebound and σ-complex mechanisms. The superscripts “s”, “t”, “q”, and “qnt” represent the singlet, triplet, quartet, and quintet ground states, respectively. Free energies calculated at the SMD/B3LYP-D3/def2-TZVP//SMD/B3LYP-D3/6-31G(d),SDD level of theory are given in kcal/mol and spin density value in e/Å^3^ (in pink).

As mentioned earlier, few dinickel dioxygen complexes have been characterized thus far. Therefore, we conducted an investigation into the formation of such a complex in the present case. After the coordination of H_2_O_2_ to ^**t**^**8** and the first deprotonation, the resulting intermediate (^**t**^**14**) can bind to the Ni center of another species ^**t**^**8** to form ^**qnt**^**20**. Et_3_N can deprotonate complex ^**qnt**^**20** with a trivial energy barrier of 2.7 kcal/mol. The ensuing species undergoes a rearrangement from the end-on to the side-on binding mode, resulting in complex ^**s**^**2**. The reaction free energy for the process of 2[Ni^II^(tepa)]^2+^ + H_2_O_2_ + 2Et_3_N → [Ni^II^(tepa)_2_O_2_]^2+**s**^**2** + 2Et_3_NH^+^ is −8.8 kcal/mol. In fact, although complex ^**s**^**2** lies outside the catalytic cycle, it serves as the resting state of the catalyst; therefore, we designated it as the new energy reference point. From the foregoing computational data, we conclude that the rate-determining step of the whole process is ^**t**^**TS**_**17**–**18**_, with a barrier height of 29.8 kcal/mol (as shown in Figure 5b), which is in line with the experiment. The catalytic cycle shown in Scheme S1 summarizes our calculation results related to the mechanism of the title reaction.

### Is the Oxygen Spin Density the Sole Crucial Feature for Benzene Addition to Oxygen?

We assumed that ^**q**^4 is directly generated from the homolytic cleavage of ^**s**^**2** in Figure 5c (the heterolytic cleavage of ^**s**^**2** is provided in Figure S4, Supporting Information). While molecule ^**q**^4 possesses a high spin density of 1.138 e/Å^3^ on oxygen, the calculated energy difference between it and the corresponding transition structure for the attack of benzene on its oxyl group (^**q**^**TS**_**4**–**3**_) is calculated to be 21.2 kcal/mol. In contrast, the analogous energy difference between ^**t**^**17** and ^**t**^**TS**_**17**–**18**_ is 11.6 kcal/mol. This can be attributed to their different abilities to generate carbocations. Both routes proceed through a carbocation structure, although after ^**t**^**TS**_**17**–**18**_, the structure involving the carbocation (^**t**^**18**′) is not a local minimum and readily transforms into ^**t**^**18**. Ni(III) in ^**t**^**17** has a higher propensity for carbocation generation compared to that of Ni(II) in ^**q**^4. The endothermicity of the reaction ^**q**^4 + benzene → ^**q**^**3** confirms this statement. ^**q**^**TS**_**4**–**3**_ is also a late transition state, as evident from the short C–O distance (1.90 Å), making it more sensitive to the stability of ^**q**^**3**. Overall, it follows that not only is the spin density on the oxyl group an important feature for the addition of benzene to oxygen but also that the positive charges on the Ni–O^•^ complex, or in other words, the oxidation state of the Ni center, is crucial.

### Is ^**qnt**^20 Capable of the Hydroxylation of Benzene?

The oxygen-rebound mechanism is commonly proposed in metal–oxygen-mediated C–H activation processes.^60−62^ This mechanism involves the initial abstraction of a hydrogen atom from the R–H substrate by M–O^•^, generating a radical R^•^ and a metal hydroxide intermediate. Subsequently, the organic radical attacks the M–OH center, leading to the formation of an alcohol group.

**Scheme 2:**
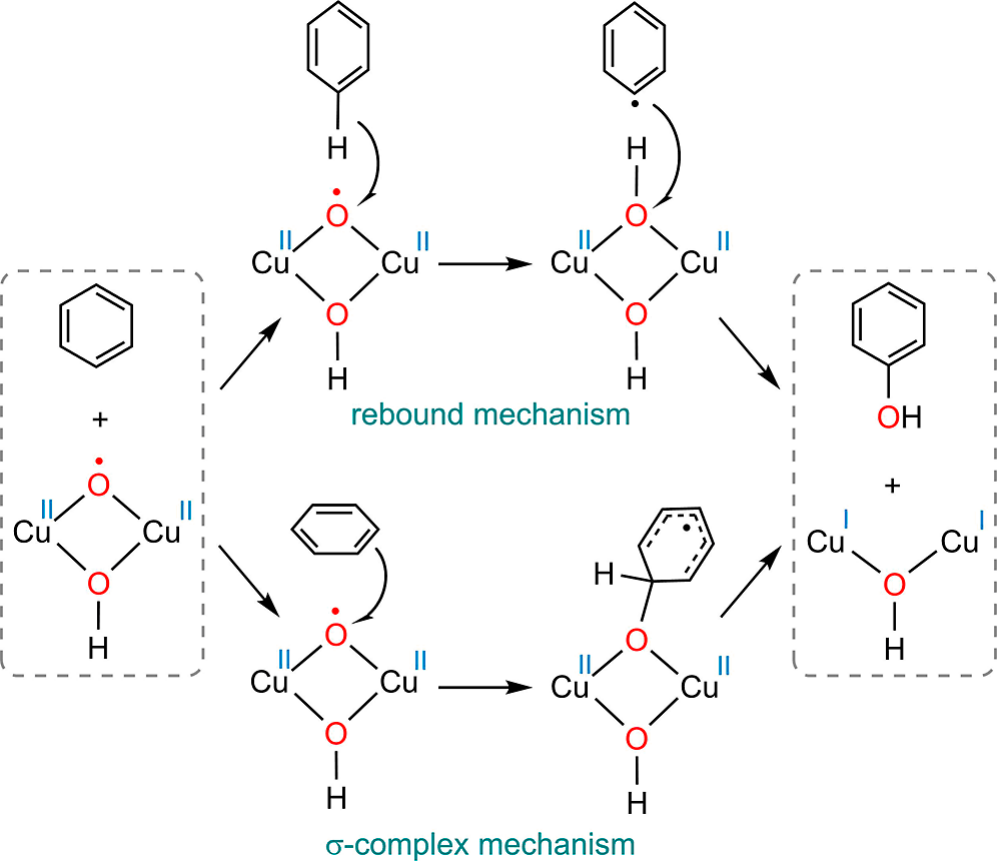
Hydroxylation of Benzene into Phenol by a Cu^II^(μ–O^•^)(μ–OH)Cu^II^ Complex via Two Different Mechanisms Reported by Lledós et al.^63^

Lledós and co-workers recently computationally demonstrated that a Cu^II^(μ–O^•^)(μ–OH)Cu^II^ complex is capable of oxidizing benzene into phenol in a stoichiometric reaction via the oxygen-rebound mechanism as well as a σ-complex mechanism (Scheme 2a).^63^ The σ-complex mechanism begins with the attack of an oxyl species on the π system of benzene, leading to the formation of a σ complex. In a subsequent step, a proton shuttle facilitates the transfer of a proton from the ipso carbon to the oxygen, producing phenol. We calculated these possibilities starting from complex ^**qnt**^**20** (Figure 5d) and found that the addition of benzene requires activation barriers as high as 38.2 and 43.2 kcal/mol via the transition states ^**qnt**^**TS**_20_^**i**^ and ^**qnt**^**TS**_20_^ii^ for the oxygen-rebound and σ-complex mechanisms, respectively. In both cases, the O–O distance in the hydroperoxo ligand elongates spontaneously without an additional transition state. This unreactivity of ^**qnt**^**20** toward the oxidation of benzene could be attributed to the lack of oxyl character of the oxygen atom. In the copper complex studied by Lledós et al., the oxygen atom possesses a spin density of 1.18 e/Å^3^,^63^ whereas this value for the analogous oxygen in ^**qnt**^**20** is only 0.094 e/Å^3^.

### Benchmark Calculations

To compare the results obtained from the SMD/B3LYP-D3/def2-TZVP//SMD/B3LYP-D3/6-31G(d),SDD level of theory with that of other established methods for organometallic transformations, we recalculated the reaction pathway using single-point calculations by M06, M06-D3, M06-L, and M06-L-D3 DFT methods along with the def2-TZVP basis set (Table 1). Furthermore, many studies have demonstrated that a modified exact exchange fraction of 15% (15% HF) for the B3LYP method, originally set at 20% (20% HF),^64^ leads to the best agreement with experiments in calculating the energy of organometallic compounds.^65−69^ Therefore, we assigned a row of benchmark calculations to the B3LYP method with an exact exchange fraction of 15%. Different levels of theory demonstrate that transition structures ^**t**^**TS**_**16**–**17**_ and ^**t**^**TS**_**17**–**18**_ lie at energies significantly lower than those of transition structure ^**t**^**TS**_**15**_. This confirms that water elimination precedes the addition of benzene to the oxyl group.

**Table 1:** Calculated Free Energies (kcal/mol) of the Key Intermediates and Transition Structures Relative to Those of ^**s**^**2** Using Different DFT Methods

	B3LYP-D3	B3LYP-D3, 15% HF	M06	M06-D3	M06-L	M06-L-D3
^**t**^**8**	4.4	5.8	1.8	3.9	8.4	11.1
^**t**^**14**	3.2	3.1	3.7	4.5	5.8	8.5
^**t**^**15**	18.1	14.1	18.7	19.4	8.9	11.6
^**t**^**TS**_**15**_	36.5	31.1	43.8	41.6	29.4	32.1
^**t**^**TS**_**16**_**_–_**_**17**_	27.6	21.6	33.5	29.1	21.4	24.1
^**t**^**17**	18.2	14.5	15.1	16.5	11.5	14.2
^**t**^**TS**_**17_–_18**_	29.8	24.1	30.8	28.7	21.6	24.3

## Conclusions

In this study, we conducted DFT calculations to interrogate the mechanistic features of the hydroxylation of benzene to phenol using H_2_O_2_ catalyzed by [Ni^II^(tepa)]^2+^. This investigation indicates that the dinickel dioxygen species, which has been proposed as the active catalyst for this transformation, is inactive toward the oxidation of benzene. Although our findings establish dinickel(II) μ–η^2^:η^2^-peroxo (^**s**^**2**) as the most stable catalyst form, its inactivity toward the hydroxylation of benzene rules this resting state out of the catalytic cycle. ^**s**^**2** could be in equilibrium with [Ni^II^(tepa)]^2+^. Then, coordinated H_2_O_2_, after deprotonation, undergoes oxidative addition at the Ni(II) center, resulting in the formation of the oxyl radical (^**t**^**15**). Addition of benzene to oxyl is the key step in this process, where the spin density value of oxygen determines the ease of this addition through a radical mechanism. Protonation of the hydroxide ligand in ^**t**^**15** renders it more susceptible to binding benzene to oxygen, so benzene is added through a radical mechanism to ^**t**^**17**. Subsequently, as the epoxide ring opens, hydrogen from the cleaved carbon migrates to a neighboring carbon, forming a ketone structure. [Ni^II^(tepa)]^2+^ can catalyze the tautomerization step to produce phenol as the final product, while water molecules can serve as a proton-shuttling agent.

The indispensable nature of this DFT study becomes evident in its ability to shed light on elusive intermediates with high energy levels, such as ^**t**^**15**, ^**t**^**16**, and ^**t**^**17**, whose experimental detection would be deemed impracticable due to their low abundance. The information provided in this research contributes to our understanding of the mechanism underlying this valuable nickel-catalyzed reaction and may guide scientists in developing novel catalytic processes for arene hydroxylation.

## Computational Details

Gaussian 16^70^ was used to fully optimize all the structures reported in this paper at the B3LYP level of theory.^71^ For all the calculations, solvent effects were considered using the SMD solvation model^72^ with acetonitrile as the solvent. The SDD basis set^73,74^ with effective core potential was chosen to describe nickel. The 6-31G(d) basis set was used for other atoms.^75^ This basis set combination will be referred to as BS1. We also employed the D3 empirical dispersion correction for all of the calculations. Frequency calculations were carried out at the same level of theory as those for structural optimization. Transition structures were located by using the Berny algorithm. IRC calculations were used to confirm the connectivity between transition structures and minima.^76,77^ To further refine the energies obtained from the SMD/B3LYP-D3/SDD,6-31G(d) calculations, we carried out single-point energy calculations using the B3LYP-D3 functional method with the SMD solvation model in acetonitrile along with a larger basis set (BS2) for all of the optimized structures. BS2 utilizes the def2-TZVP basis set^78^ on all atoms. The tight convergence criterion and ultrafine integral grid were exploited to increase the accuracy of the calculations. The free energy for each species in solution was calculated using the following formula
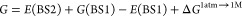
1where Δ*G*^1atm→1M^ = 1.89 kcal/mol is the free-energy change for compression of 1 mol of an ideal gas from 1 atm to the 1 M solution phase standard state. It is worth noting that different spin states have been computed in the energy calculations of nickel complexes, and the most stable ones have been reported.

## Supporting Information Available

Additional computational results and Cartesian coordinates of all calculated species (PDF)
