# Supplementary material for: DFT Mechanistic Investigation into Ni(II)-Catalyzed Hydroxylation of Benzene to Phenol by H2O2
Source: Inorg Chem. 2024 Mar 12;63(12):5509–19. doi: 10.1021/acs.inorgchem.3c04461 (PMC11186014; doi:10.1021/acs.inorgchem.3c04461)
Supplement: Supplementary file 1 — ic3c04461_si_001.pdf [file ic3c04461_si_001.pdf]

DFT Mechanistic Investigation into Ni(II)-Catalyzed Hydroxylation of Benzene to Phenol by H_2O_2

Kaveh Farshadfar,* and Kari Laasonen*

Department of chemistry and material science, School of chemical engineering, Aalto University, 02150 Espoo, Finland

Email: Kaveh.Farshadfar@aalto.fi, Kari.Laasonen@aalto.fi

Content:

Figure S1. Relative free energy of different dinickel bis(μ-oxo) and μ-η2:η2-peroxo isomer forms. (page S2)

Figure S2. DFT calculated reaction potential pathways potential substitution reactions benzene and **2**^{S} and addition of benzene to ^{s}**2**. (page S3)

Figure S3. Stability comparison between complexes with mono- and bi-coordinated solvents to nickel relative to $[Ni^{II}(tepa)]^{2+}$ (^{t}**8**). (page S4)

Other pathways to generate Ni-oxyl species (page S4)

Figure S4. Calculated mechanism for heterolytic cleavage of ^{s}**2**. (page S5)

Figure S5. Spin density distribution plots for key Ni species at an isosurface value of 0.006. (page S6)

Scheme S1. DFT-proposed catalytic cycle for hydrogenation of benzene using H_2O_2 catalyzed by $[Ni^{II}(tepa)]^{2+}$ (^{t}**8**). (page S7)

Table S1. Cartesian coordinates and total energies for all of the calculated structures. (page S8)

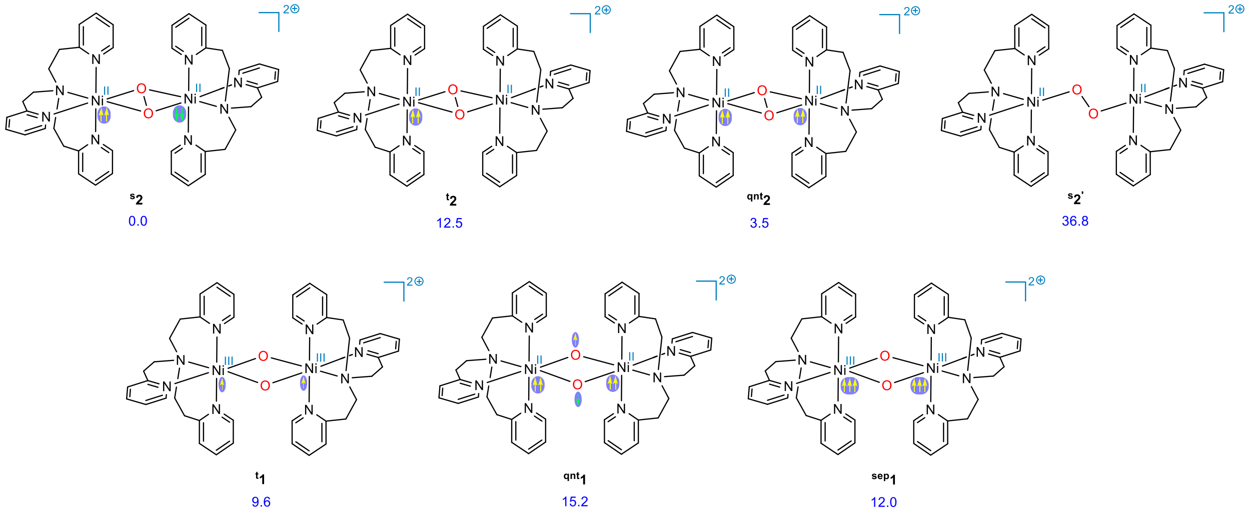

Figure S1. Relative free energy of different dinickel bis(μ-oxo) and μ-η^2:η^2-peroxo isomer forms. The superscripts "s", "t", "qnt", and "sep" represent the singlet, triplet, quintet, and septet ground states, respectively. Free energies calculated at the SMD/B3LYP-D3/def2-TZVP//SMD/B3LYP-D3/6-31G(d),SDD level of theory level of theory are given in kcal/mol (in blue). Our attempts to locate other potential bis(μ-oxo) and μ-η^2:η^2-peroxo ground states were unsuccessful and collapsed to one of the given structures.

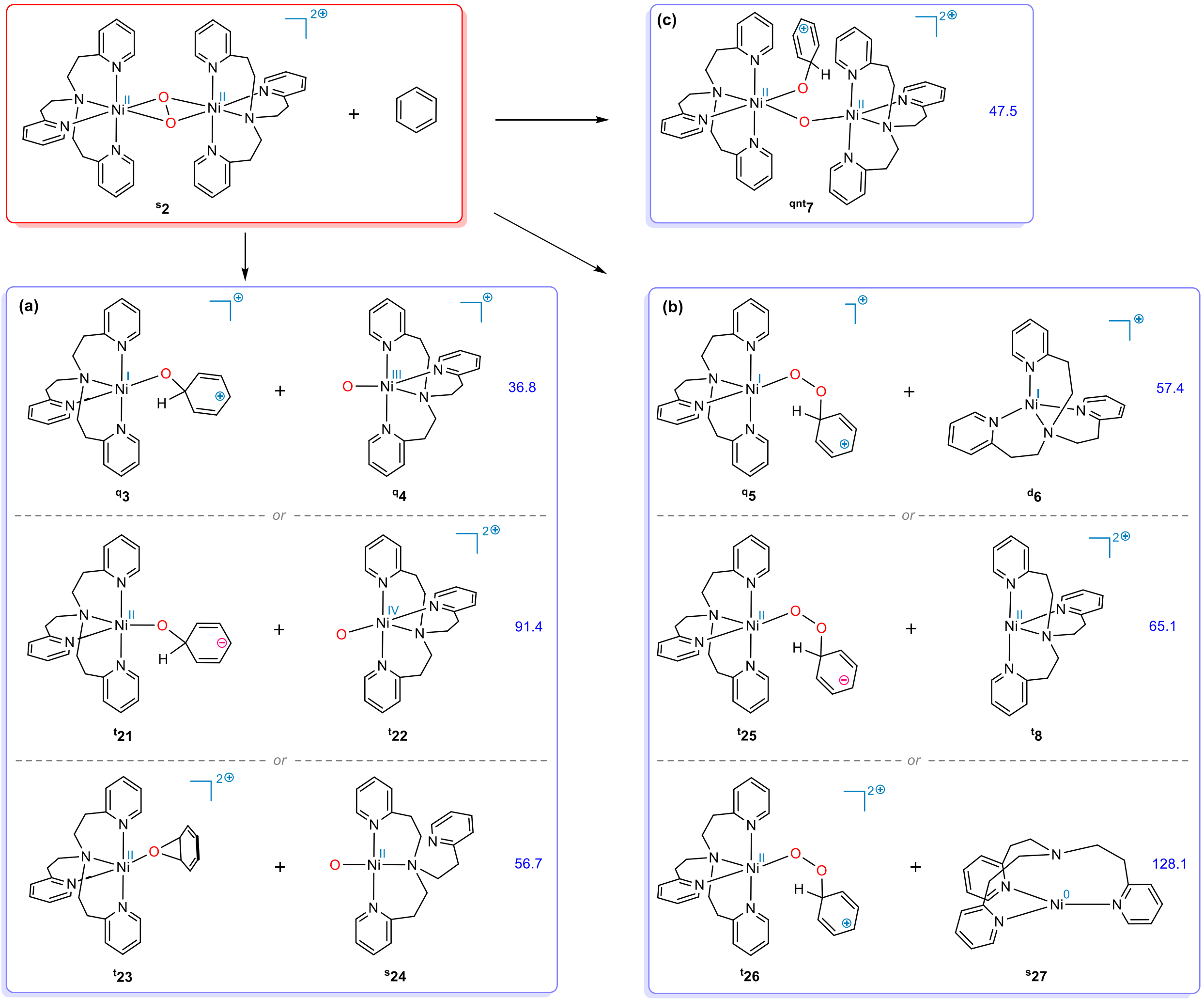

Figure S2. DFT calculated reaction potential pathways at the SMD/B3LYP-D3/def2-TZVP//SMD/B3LYP-D3/6-31G(d),SDD level of theory for the (a) and (b) potential substitution reactions benzene and ^{s}**2** (c) addition of benzene to ^{s}**2**. The superscripts "s", "d", "t", "q", and "qnt" represent the singlet, doublet, triplet, quartet, and quintet ground states, respectively. Free energies are given in kcal/mol (in blue)

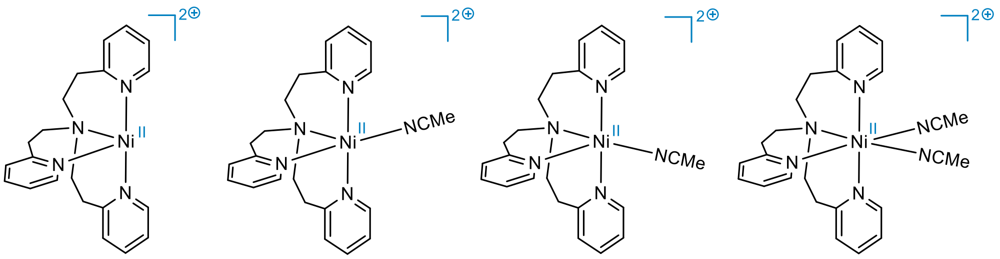

^{t}**8**	^{t}**8**^{i}	^{t}**8**^{ii}	^{t}**8**^{iii}
0.0	0.8	0.9	3.1

Figure S3. Stability comparison between complexes with mono- and bi-coordinated solvents to nickel relative to [Ni^{II}(tepa)]$^{2+}$ (^{t}**8**). The superscript "t" represents the triplet ground states. The relative Gibbs free obtained from the SMD/B3LYP-D3/def2-TZVP//SMD/B3LYP-D3/6-31G(d),SDD calculations are given in kcal/mol (in red).

Other pathways to generate Ni-oxyl species

We thoroughly explored the other potential pathways for Ni-oxyl species generation as intermediates for this transformation. As illustrated in Figure 5c, homolytic cleavage of ^{s}**2** can generate ^{q}**4**, which exhibits high activation energies for benzene binding and, therefore, cannot occur. Heterolytic cleavage of ^{s}**2** results in the generation of ^{t}**17** alongside ^{t}**28**; however, this reaction is strongly thermodynamically unfavorable. We also explored the generation of ^{t}**17** through the decomposition of ^{qnt}**20** into ^{t}**17** and ^{t}**12**, and it was found that there is a high energy barrier for this dissociation (Figure S4).

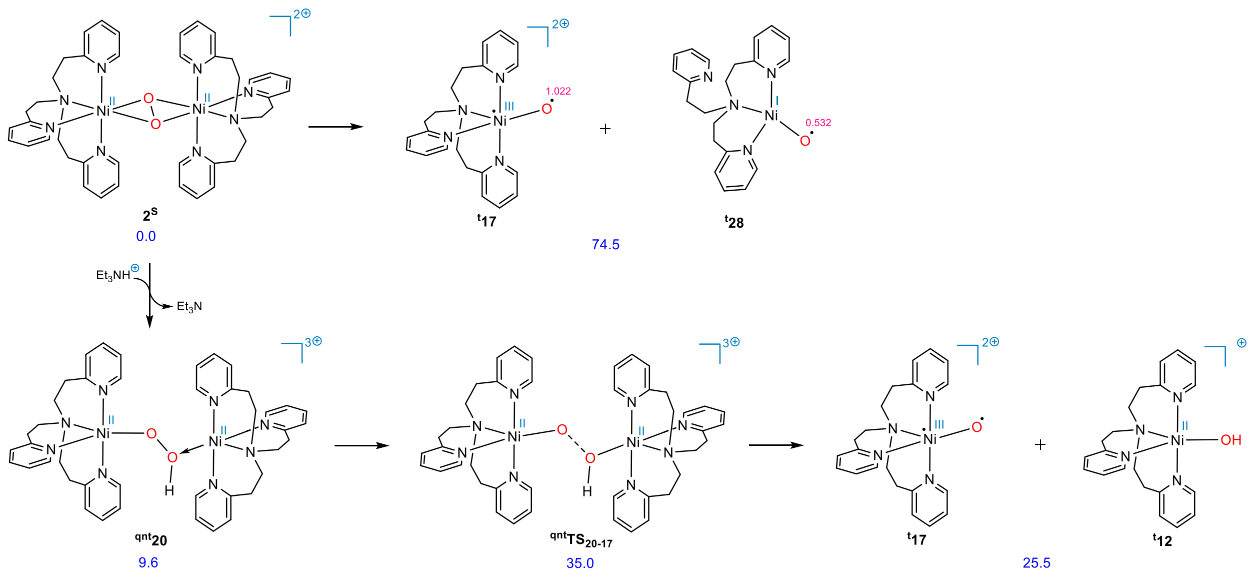

Figure S4. Calculated mechanism for heterolytic cleavage of ^{s}**2** resulting in the generation of ^{t}**17** and ^{t}**18** (top) and for production of ^{t}**17** from decomposition of ^{qnt}**20** (bottom). The superscripts "s", "t", and "qnt" represent the singlet, triplet, and quintet ground states, respectively. The relative Gibbs free obtained from the SMD/B3LYP-D3/def2-TZVP//SMD/B3LYP-D3/6-31G(d),SDD calculations are given in kcal/mol (in blue). The relative Gibbs free obtained from the SMD/B3LYP-D3/def2-TZVP//SMD/B3LYP-D3/6-31G(d),SDD calculations are given in kcal/mol (in blue) and spin density values in e/Å^{3} (in pink).

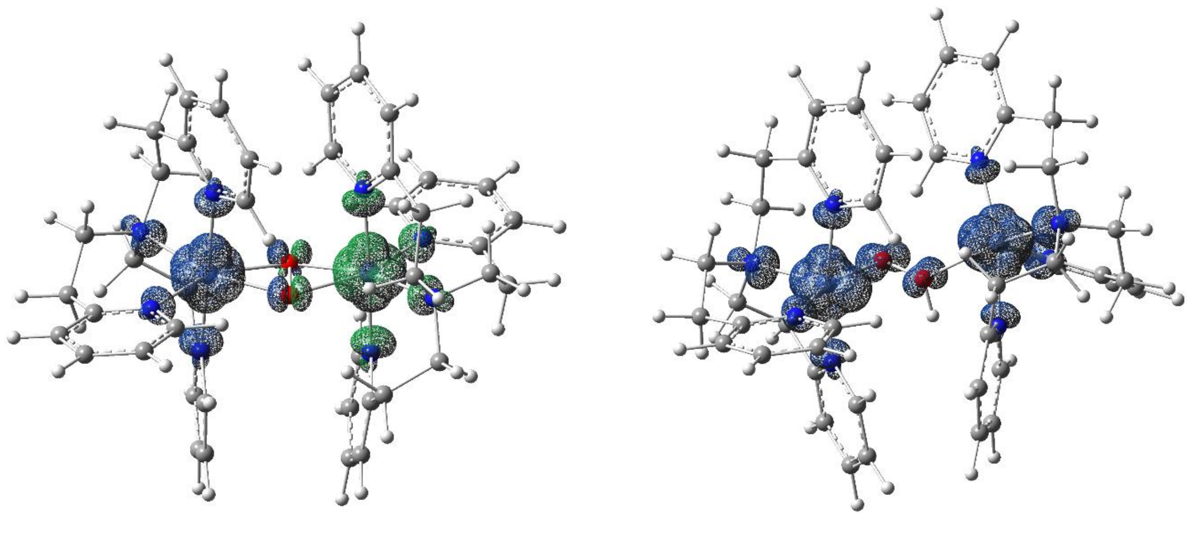

^{s}**2** ^{qnt}**20**

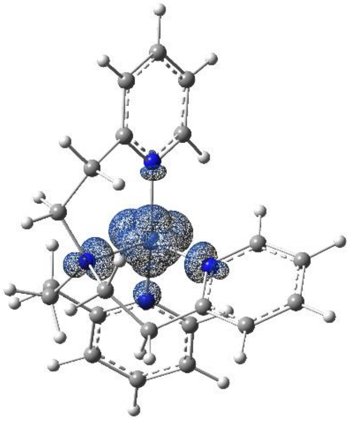

^{t}8

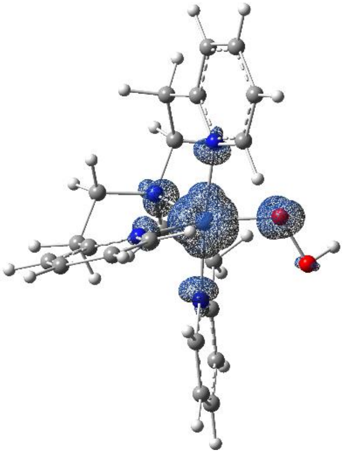

^{t}14

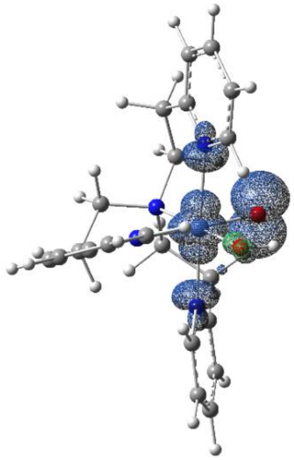

^{t}15

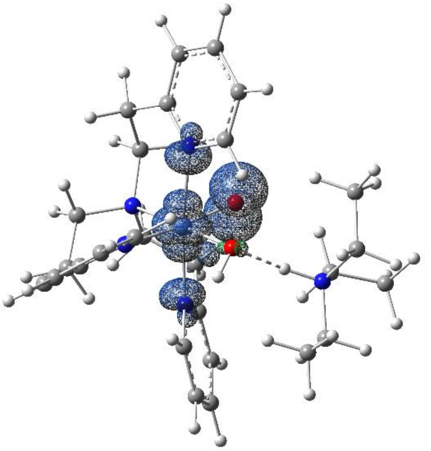

^{t}16

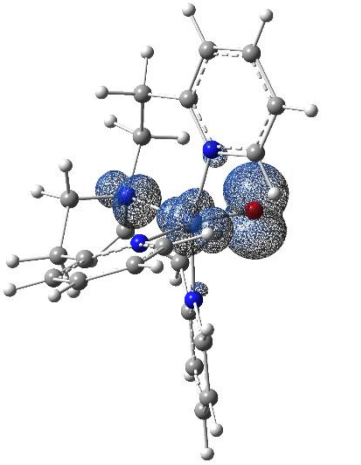

^{t}17

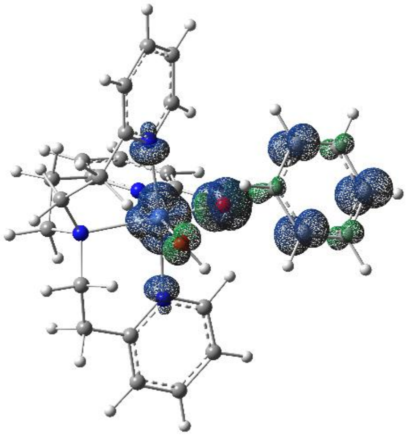

$^{t}TS_{15}$

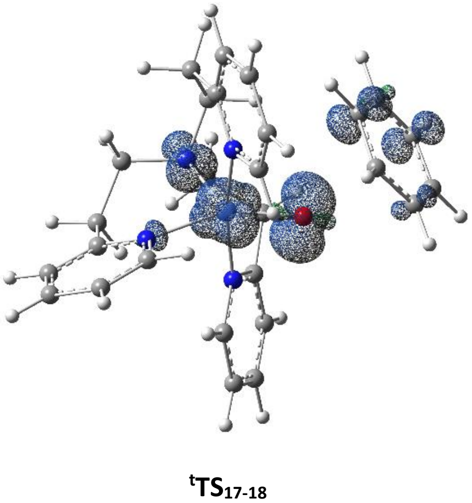

$^{t}TS_{17\text{-}18}$

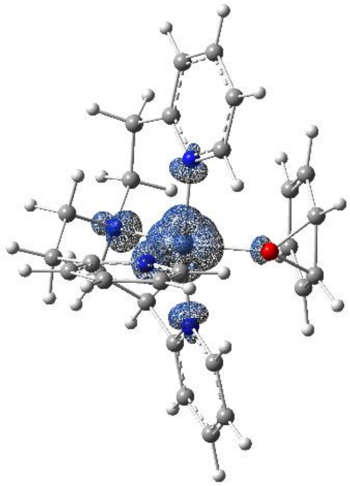

^{t}18

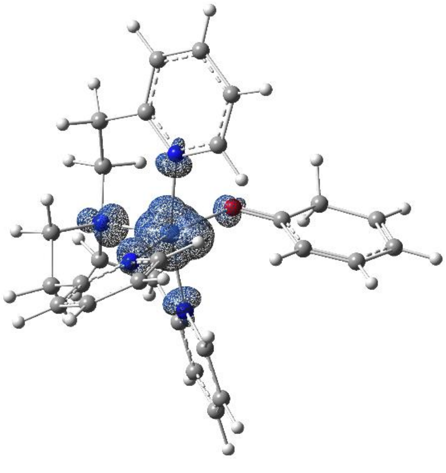

^{t}19

Figure S5. Spin density distribution plots for key Ni species at an isosurface value of 0.006. The superscripts "s", "t", and "qnt" represent the singlet, triplet, and quintet ground states, respectively.

Scheme S1. DFT-proposed catalytic cycle for hydrogenation of benzene using H_2O_2 catalyzed by $[Ni^{II}(tepa)]^{2+}$ (^{t}**8**). The superscripts "s", and "t" represent the singlet, and triplet ground states, respectively.

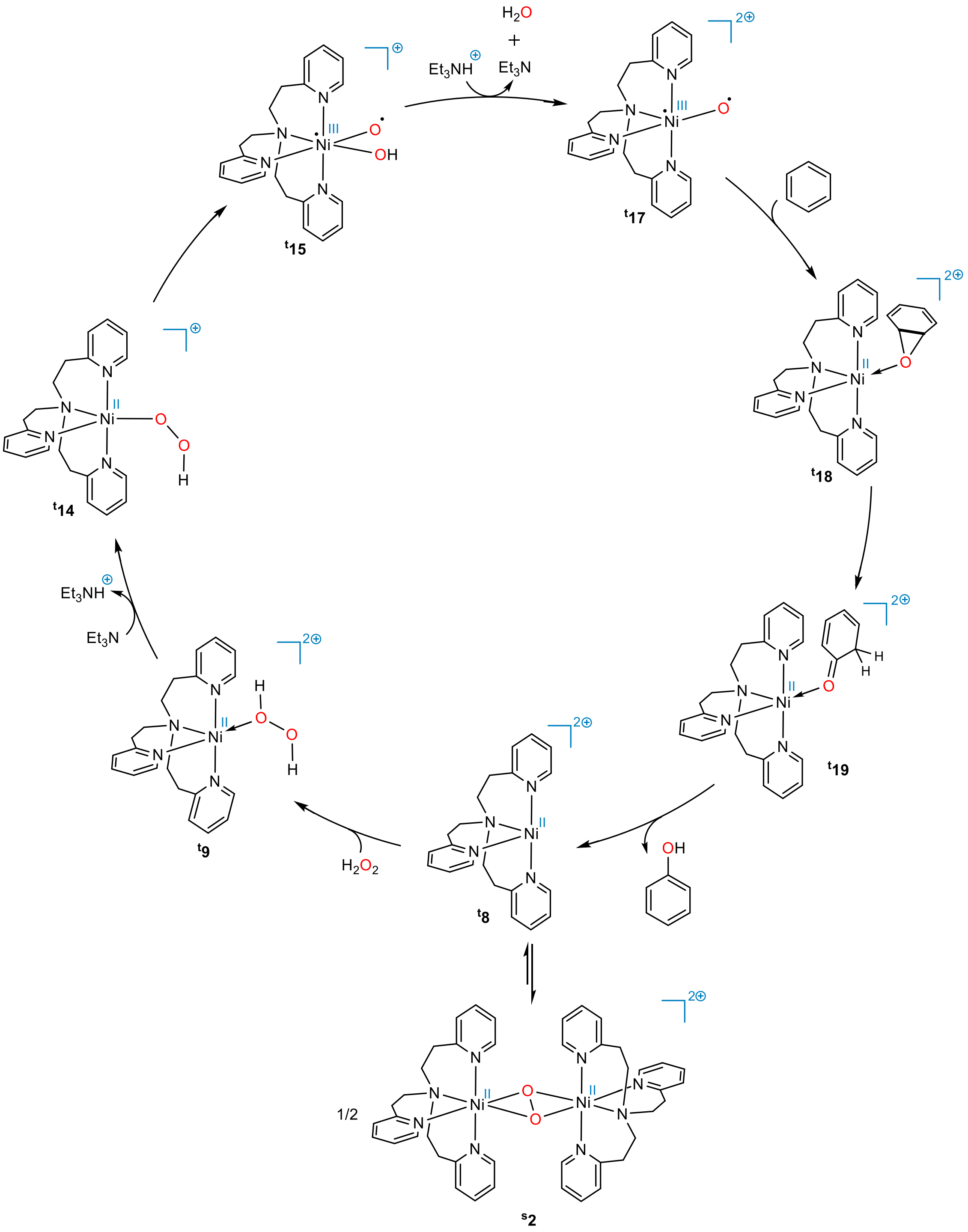

Table S1. Total potential (E), and Gibbs free energies (G) of all structures optimized at the SMD/B3LYP-D3/BS1 level of theory along with the total potential energies calculated by SMD/B3LYP-D3/BS2//SMD/ B3LYP-D3/BS1 and Cartesian coordinates for all of the calculated structures, as well as eigenvalues of imaginary frequencies of transition states.

^{s}**2**

E (SMD/B3LYP-D3/BS1) = -2559.785949 au
G (SMD/B3LYP-D3/BS1) = -2559.013923 au
E (SMD/B3LYP-D3/BS2//SMD/B3LYP-D3/BS1) = -5235.45509036 au
charge = 0 spin multiplicity = 1

Ni	1.81928200	-0.02092700	0.25851500
N	3.11755700	0.21027000	2.03924300
C	3.13476700	-1.02347600	2.88927300
H	3.99168200	-1.63378600	2.60049000
C	4.50390000	0.55739800	1.62317500
H	5.15466300	0.57844900	2.50910500
H	4.49336800	1.56108000	1.19851300
C	2.66925800	2.74396400	2.36295100
C	1.91266100	-2.80019700	1.58977600
C	1.92594400	-4.19370600	1.70352400
C	2.02316400	-4.97380100	0.55216200
H	1.86122400	-4.65177700	2.68535000
C	2.08381400	-2.95165000	-0.72432700
C	2.10345400	-4.34282900	-0.69009700
H	2.03123100	-6.05765900	0.62319900
H	2.13738100	-2.40470100	-1.65976000
H	2.17092100	-4.90955400	-1.61265600
C	4.73364500	-0.10284300	-0.86233900
C	5.71922600	-0.01496900	-1.85285800
C	3.07376400	0.21570500	-2.46512200
C	5.35246800	0.18956100	-3.18105100
H	6.76359700	-0.11316800	-1.57453800
C	3.99814300	0.29954200	-3.49926800
H	2.00735500	0.28665100	-2.65124600
H	6.11128600	0.25725500	-3.95532900
H	3.66134700	0.44699100	-4.51995500
C	2.16063600	3.16204200	1.00255700
C	2.17273700	4.52843500	0.67911300
C	1.66054600	4.95498700	-0.53950400
H	2.57276700	5.23891000	1.39647300
C	1.14951000	2.67089400	-1.02522700
C	1.12386300	4.00325900	-1.41152100
H	1.66145600	6.00973500	-0.79924900
H	0.71007600	1.88844300	-1.63078500
H	0.68579300	4.28550600	-2.36311800
N	1.67506200	2.25036200	0.14418300
N	1.99655600	-2.20267300	0.38470300

N	3.42837500	0.02893200	-1.17986700
C	2.47199700	1.30381900	2.83436000
H	2.86094200	1.26828900	3.86159200
H	1.40408100	1.06969100	2.87933600
H	3.72228800	3.03496900	2.45226900
H	2.14669200	3.36300900	3.10418000
C	5.10348900	-0.39580800	0.57282600
H	4.82154500	-1.43284500	0.79047500
H	6.19262400	-0.34866900	0.66277600
H	3.29823800	-0.72667100	3.93281800
C	1.86503100	-1.88939100	2.78885400
H	0.97510900	-1.25274100	2.72387500
H	1.78132500	-2.49013400	3.69933000
O	-0.19194900	-0.23888300	0.72855500
Ni	-1.91147900	-0.09932100	-0.29326600
N	-3.15875200	0.07116100	-2.01769400
C	-3.12737300	-1.15826500	-2.87361600
H	-3.92158000	-1.83017200	-2.54630600
C	-4.56514300	0.36213100	-1.62189800
H	-5.20089400	0.36330300	-2.51886100
H	-4.60055100	1.36282600	-1.19158400
C	-2.81918500	2.61312700	-2.32865400
C	-1.70384300	-2.81663800	-1.62524300
C	-1.46445100	-4.19050400	-1.72068800
C	-1.41200200	-4.95867900	-0.55878200
H	-1.31888100	-4.64239600	-2.69676800
C	-1.83970200	-2.96287900	0.68804500
C	-1.60440400	-4.33415600	0.67420400
H	-1.21918700	-6.02616200	-0.61437300
H	-1.98282300	-2.42413700	1.61775500
H	-1.56462900	-4.88932200	1.60531800
C	-4.75329500	-0.35815800	0.84697600
C	-5.71728300	-0.35730600	1.86253900
C	-3.07177100	-0.03232000	2.42135800
C	-5.32806100	-0.19212500	3.18934300
H	-6.76244400	-0.49232900	1.60327600
C	-3.97205200	-0.03518200	3.47932800
H	-2.00574200	0.07378600	2.58794900
H	-6.06937800	-0.19171900	3.98329400
H	-3.61522900	0.08241900	4.49708700
C	-2.34784500	3.00704700	-0.94914300
C	-2.40523400	4.36346000	-0.59302100
C	-1.92270900	4.77648000	0.64180700
H	-2.81489800	5.07815900	-1.30066500
C	-1.34980400	2.49623500	1.07612300
C	-1.36776300	3.81871400	1.49453600
H	-1.95822000	5.82398400	0.92703500

H	-0.88678800	1.71842700	1.66772800
H	-0.94625900	4.08843700	2.45721500
N	-1.85126900	2.08290100	-0.10764200
N	-1.89382100	-2.22422100	-0.43016200
N	-3.44727500	-0.17790600	1.13586500
C	-2.56301300	1.19001900	-2.81619300
H	-2.95416600	1.14078700	-3.84185000
H	-1.48607200	1.00532100	-2.86345400
H	-3.87959400	2.87097800	-2.43073700
H	-2.30285100	3.26560400	-3.04505700
C	-5.14721000	-0.61492500	-0.58762300
H	-4.87427500	-1.64833800	-0.83320800
H	-6.23759400	-0.56253000	-0.66012700
H	-3.36314600	-0.87000400	-3.90585200
C	-1.79788400	-1.92802600	-2.83712200
H	-0.95117500	-1.23220800	-2.83578900
H	-1.72398000	-2.53754700	-3.74269000
O	0.06293900	-0.19526900	-0.77068900

^{q}3

E (SMD/B3LYP-D3/BS1) = -1512.12073459 au
G (SMD/B3LYP-D3/BS1) = -1511.654576 au
E (SMD/B3LYP-D3/BS2//SMD/B3LYP-D3/BS1) = -2850.04852413 au
charge = 1 spin multiplicity = 4

Ni	0.17872300	0.01435400	-0.11724600
N	0.50438400	0.02955600	2.01549600
C	0.27712700	-1.33715300	2.60406800
H	1.24403400	-1.82802800	2.72203000
C	1.87707600	0.49286100	2.36512700
H	2.03347800	0.35585400	3.44422500
H	1.94144800	1.56077000	2.15609300
C	-0.44734100	2.42914900	2.27826900
C	0.10609400	-2.90107600	0.63683900
C	0.17245000	-4.28673100	0.46844800
C	0.91040800	-4.81372100	-0.59118000
H	-0.34858000	-4.93696000	1.16408800
C	1.46621900	-2.57452100	-1.22181000
C	1.57543500	-3.94225400	-1.45504500
H	0.96877700	-5.88851100	-0.73767800
H	1.95710700	-1.84865600	-1.86276400
H	2.16341400	-4.30731500	-2.29069600
C	3.25133900	0.34030900	0.20883400
C	4.55418300	0.61707900	-0.22094300
C	2.41190500	0.99784400	-1.85781800
C	4.77378800	1.09106500	-1.51158500
H	5.38298900	0.45577500	0.46089800
C	3.67792300	1.28365200	-2.35351100

H	1.52981800	1.12472600	-2.47450900
H	5.78185100	1.30615600	-1.85395400
H	3.79388600	1.64560900	-3.36951900
C	-0.68011200	2.89796300	0.86130200
C	-1.12323800	4.21266400	0.65130500
C	-1.31875800	4.68324000	-0.64157700
H	-1.31167200	4.85026100	1.50951300
C	-0.65539100	2.53030400	-1.43175300
C	-1.08038500	3.82019700	-1.71368000
H	-1.65997000	5.70030400	-0.81147400
H	-0.48492700	1.81136500	-2.22545200
H	-1.22887600	4.13106300	-2.74230300
N	-0.44544500	2.07531100	-0.17833000
N	0.75085700	-2.06933900	-0.20618800
N	2.19542000	0.54148500	-0.60884700
C	-0.52808800	0.93685300	2.60748300
H	-0.46563500	0.85883400	3.70227900
H	-1.49907600	0.53845100	2.30450300
H	0.50680700	2.84559700	2.62303600
H	-1.20445700	2.90810600	2.90998600
C	2.99694500	-0.21435200	1.58791200
H	2.78714500	-1.28657600	1.49711900
H	3.91866100	-0.12824400	2.17031300
H	-0.13796700	-1.21324200	3.61096600
C	-0.63842500	-2.25400200	1.77657300
H	-1.48874200	-1.68553600	1.37996200
H	-1.03819200	-3.02953600	2.43688000
O	-1.39441500	-0.62477700	-0.99070300
C	-3.50566400	-1.04795700	-1.98914700
C	-2.67731500	-0.06173500	-1.20061300
C	-3.34142200	0.31224600	0.10221600
C	-4.48260900	-0.29183600	0.55951300
C	-5.15475200	-1.27093800	-0.21944300
C	-4.64559500	-1.62454900	-1.49923000
H	-3.10997600	-1.33434300	-2.96149700
H	-2.58378700	0.86439300	-1.80573000
H	-2.84179600	1.07251000	0.69341800
H	-4.89065400	-0.01791000	1.53108000
H	-6.05613000	-1.74474900	0.15869300
H	-5.17516800	-2.37056600	-2.08925300

[q]4

E (SMD/B3LYP-D3/BS1) = -1279.85203632 au

G (SMD/B3LYP-D3/BS1) = -1279.480097 au

E (SMD/B3LYP-D3/BS2//SMD/B3LYP-D3/BS1) = -2617.69109016 au

charge = 1 spin multiplicity = 4

Ni	-0.02019300	-0.36969900	-0.58155800

N	0.08337300	-1.29735500	1.38125400
C	1.36063300	-2.07467700	1.55110200
H	2.07222900	-1.45916200	2.10295000
C	-0.02993700	-0.30229500	2.48375100
H	0.15942400	-0.80485500	3.44244100
H	-1.05272500	0.07303000	2.50534400
C	-2.47267100	-1.75333100	1.46221700
C	2.81149400	-1.40724100	-0.39428400
C	4.18892900	-1.48168600	-0.61748900
C	4.85472400	-0.38451200	-1.16263700
H	4.72444700	-2.39037700	-0.36186300
C	2.76126800	0.77172700	-1.21646100
C	4.12937000	0.76855500	-1.46762200
H	5.92466000	-0.42817300	-1.34472600
H	2.14329000	1.63685000	-1.43497100
H	4.60667100	1.64562800	-1.89176300
C	0.40081400	1.97610900	1.40864100
C	0.42774200	3.32219800	1.78992700
C	-0.51495300	2.55948300	-0.64886100
C	-0.02834500	4.30182300	0.91199700
H	0.80683500	3.58760200	2.77162800
C	-0.51082900	3.91356700	-0.33839400
H	-0.87425300	2.20788500	-1.60918900
H	-0.00928700	5.34905700	1.19920200
H	-0.87599600	4.63664000	-1.05983300
C	-3.02740700	-1.05031800	0.24722200
C	-4.41946000	-0.98408800	0.08272300
C	-4.95876200	-0.35003100	-1.02966400
H	-5.06190500	-1.43867500	0.83068800
C	-2.72508700	0.09096100	-1.75303800
C	-4.08972300	0.19462900	-1.97840900
H	-6.03509600	-0.29276600	-1.16367500
H	-2.00532400	0.47731400	-2.46531700
H	-4.45655400	0.68411700	-2.87440500
N	-2.19801100	-0.49879200	-0.65722200
N	2.12267600	-0.28690700	-0.69300000
N	-0.07327700	1.60910800	0.19832300
C	-1.04041900	-2.28798500	1.42092300
H	-0.91771600	-2.91138000	2.31770300
H	-0.91387200	-2.93573500	0.54772000
H	-2.62598900	-1.11393100	2.33951200
H	-3.10489400	-2.63009800	1.64593100
C	0.91424600	0.90046400	2.33212600
H	1.90026700	0.57697000	1.97796600
H	1.07016100	1.33781500	3.32247200
H	1.15167400	-2.95058400	2.17591100
C	2.02511000	-2.52588200	0.23957700

H	1.26504300	-2.89399500	-0.46060700
H	2.69555100	-3.36131900	0.46171600
O	0.01187800	-1.01468500	-2.27959900

^{q}TS$_{4\text{-}3}$

E (SMD/B3LYP-D3/BS1) = -1512.09654184 au
G (SMD/B3LYP-D3/BS1) = -1511.631021 au
E (SMD/B3LYP-D3/BS2//SMD/B3LYP-D3/BS1) = -2850.02493713 au
negative eigenvalue of the frequency: 537i
charge = 1 spin multiplicity = 4

Ni	-0.14567300	-0.00522400	-0.15918300
N	-0.36656500	0.02845900	1.98391700
C	-0.16032400	1.41382400	2.53333800
H	-1.13726600	1.87064400	2.69675400
C	-1.69598500	-0.48209100	2.41963600
H	-1.79599500	-0.34006300	3.50496900
H	-1.73208600	-1.55380900	2.22316600
C	0.66886400	-2.33131500	2.27018300
C	-0.15866900	2.94490800	0.53334000
C	-0.28901500	4.32403400	0.34851700
C	-1.10176200	4.80457300	-0.67800000
H	0.24167400	5.00555300	1.00595700
C	-1.60135800	2.53497200	-1.24416000
C	-1.77592900	3.89341900	-1.49244900
H	-1.21002600	5.87376800	-0.83695600
H	-2.09771200	1.78031100	-1.84648400
H	-2.42085700	4.22132100	-2.30113200
C	-3.19664500	-0.40269100	0.34403100
C	-4.51498900	-0.71052300	-0.01196500
C	-2.45826300	-1.05479300	-1.76035500
C	-4.79529200	-1.19696600	-1.28592300
H	-5.30851700	-0.56203500	0.71336900
C	-3.74299000	-1.36965200	-2.18560300
H	-1.61029200	-1.16402400	-2.42618900
H	-5.81567000	-1.43570600	-1.57144100
H	-3.90604600	-1.73798700	-3.19286500
C	0.74492300	-2.86016800	0.85730000
C	1.10514100	-4.20301800	0.66450600
C	1.19292200	-4.72288700	-0.62061800
H	1.31987000	-4.82110500	1.53093000
C	0.58410700	-2.56420900	-1.43914000
C	0.93116800	-3.88030100	-1.70406500
H	1.47160100	-5.76087800	-0.77746200
H	0.39601800	-1.86313200	-2.24422000
H	1.00014100	-4.22849800	-2.72914000
N	0.47710800	-2.05983000	-0.19114900
N	-0.81466700	2.07417600	-0.26065000

N	-2.18130500	-0.58787100	-0.52712900
C	0.73928200	-0.82431400	2.52116300
H	0.77493600	-0.69843700	3.61285800
H	1.66747500	-0.41867700	2.11201500
H	-0.21871800	-2.75920600	2.75112500
H	1.51555700	-2.75784300	2.82132900
C	-2.88425600	0.17406800	1.70192100
H	-2.72051300	1.25225600	1.58852100
H	-3.76772100	0.06173700	2.33703100
H	0.31437400	1.32669500	3.51762900
C	0.67214000	2.34780100	1.64007700
H	1.51699000	1.80290200	1.20238700
H	1.07817600	3.15035800	2.26345900
O	1.29501600	0.71498900	-1.08735300
C	3.68839700	0.91950900	-2.06921200
C	2.97111600	-0.12401900	-1.39505800
C	3.38913000	-0.45428400	-0.06739800
C	4.27966300	0.36189000	0.61935800
C	4.86552000	1.46431500	-0.02214700
C	4.57877300	1.72164800	-1.37922900
H	3.47132600	1.11144300	-3.11668400
H	2.52958400	-0.90738200	-1.99929900
H	2.96089100	-1.32449100	0.41555300
H	4.54220500	0.13250800	1.64949500
H	5.56657100	2.09841400	0.51423500
H	5.07673900	2.54481300	-1.88617400

q5

E (SMD/B3LYP-D3/BS1) = -1587.26009201 au

G (SMD/B3LYP-D3/BS1) = -1586.792959 au

E (SMD/B3LYP-D3/BS2//SMD/B3LYP-D3/BS1) = -2925.22177051 au

charge = 1 spin multiplicity = 4

O	1.91348800	1.54526600	-0.89077800
Ni	-0.38134300	-0.04178700	-0.22580500
N	-1.83745000	-0.60027200	-1.64597300
C	-1.45345700	-1.84824500	-2.38772000
H	-1.85817100	-2.70633000	-1.85122600
C	-3.16607700	-0.79510300	-0.99747600
H	-3.88277100	-1.13265900	-1.75925400
H	-3.50535800	0.17235600	-0.62731100
C	-2.41253500	1.87757700	-2.15343200
C	0.73739300	-2.62143100	-1.34980100
C	1.55848100	-3.75159000	-1.40782700
C	2.16939600	-4.22152000	-0.24571900
H	1.71336400	-4.25136900	-2.35874100
C	1.09931500	-2.44667300	0.94416200
C	1.93430100	-3.55883600	0.95944400

H	2.81495400	-5.09440600	-0.28035500
H	0.88318600	-1.88985000	1.85020700
H	2.38460700	-3.88915400	1.88952000
C	-2.68879900	-1.24148900	1.49964900
C	-3.39593800	-1.50298100	2.67944500
C	-1.05404900	-0.11734600	2.71185000
C	-2.89948700	-1.05584300	3.90101600
H	-4.32648000	-2.05914500	2.62854800
C	-1.69507400	-0.35147300	3.92156700
H	-0.11204100	0.41969100	2.67303900
H	-3.44126700	-1.25630400	4.82079300
H	-1.25941500	0.01076300	4.84654400
C	-1.64782600	2.65858400	-1.10855500
C	-1.70651900	4.05939000	-1.13683900
C	-1.06499000	4.80489700	-0.15321000
H	-2.25900600	4.55012800	-1.93203200
C	-0.33064600	2.74504000	0.80281000
C	-0.36004800	4.13225700	0.84577800
H	-1.10893900	5.89010800	-0.16915800
H	0.22740800	2.18195600	1.54156600
H	0.16596200	4.66296200	1.63230300
N	-0.96567300	2.01895000	-0.13817200
N	0.52286000	-1.98711400	-0.17780000
N	-1.53574200	-0.54051600	1.52752000
C	-1.87795300	0.52460200	-2.63507900
H	-2.51509500	0.21731000	-3.47639600
H	-0.85672100	0.64870800	-3.00015200
H	-3.45085300	1.78037500	-1.81279200
H	-2.47012000	2.50163800	-3.05208800
C	-3.17681100	-1.78380000	0.17860000
H	-2.58960800	-2.67935800	-0.05742300
H	-4.20783200	-2.12571300	0.30826700
H	-1.94340800	-1.82519800	-3.36899200
C	0.05667600	-2.06164000	-2.57471100
H	0.54746400	-1.12008900	-2.84364600
H	0.19772200	-2.75619600	-3.40853500
O	1.08381900	0.49684500	-1.47661000
C	3.32327900	2.11920400	0.92383900
C	2.64171100	0.98198500	0.22363400
C	3.57665800	-0.11578400	-0.19450400
C	4.91134200	-0.08467900	0.10412000
C	5.48161400	1.00584000	0.81432300
C	4.66310500	2.10033700	1.20128800
H	2.69788100	2.96117100	1.20785500
H	1.87607900	0.55508600	0.90857200
H	3.13460900	-0.93209500	-0.75505000
H	5.55662900	-0.90037700	-0.21630300

H	6.54367400	1.01456400	1.04025100
H	5.11635000	2.93957400	1.72470500

^{d}**6**

E (SMD/B3LYP-D3/BS1) = -1204.66841693 au

G (SMD/B3LYP-D3/BS1) = -1204.300702 au

E (SMD/B3LYP-D3/BS2//SMD/B3LYP-D3/BS1) = -2542.48175388 au

charge = 1 spin multiplicity = 2

Ni	0.00392000	0.00214300	-0.21426800
N	0.00094700	0.00877100	1.88542000
C	-0.46932900	-1.31249800	2.39691800
H	0.36836600	-2.01196900	2.35104300
C	1.38047800	0.26461900	2.39462300
H	1.42591900	-0.02336800	3.45469800
H	1.56599400	1.33991700	2.34467100
C	-0.83346800	2.39650000	1.60170500
C	-1.25600100	-2.62877000	0.35189400
C	-1.60702500	-3.96180700	0.12307500
C	-1.23462600	-4.58345500	-1.06844400
H	-2.17336100	-4.49956000	0.87709400
C	-0.17918200	-2.53377300	-1.70981800
C	-0.50654600	-3.85095000	-2.00825100
H	-1.50601700	-5.61779200	-1.25852700
H	0.39840600	-1.92495600	-2.39985600
H	-0.19127200	-4.28779900	-2.95030100
C	2.91143600	0.23222200	0.34672600
C	4.24024800	0.59742100	0.11558300
C	2.28888400	1.09724300	-1.72300200
C	4.59094400	1.22168900	-1.08110700
H	4.98941300	0.38482400	0.87200200
C	3.59218200	1.47392400	-2.02390600
H	1.47286400	1.28387400	-2.41563000
H	5.62148400	1.50577400	-1.27312200
H	3.81179500	1.95752600	-2.97021200
C	-1.65764500	2.40239900	0.33947400
C	-2.64543700	3.36306000	0.10747800
C	-3.36915500	3.34131800	-1.08439500
H	-2.83571600	4.12245600	0.85957100
C	-2.10331800	1.41244000	-1.71987100
C	-3.08867700	2.34471300	-2.02150500
H	-4.13597300	4.08593900	-1.27709100
H	-1.85681700	0.60846500	-2.40788600
H	-3.62325300	2.28277400	-2.96387000
N	-1.39655600	1.43180300	-0.56940600
N	-0.54176400	-1.92563800	-0.56006900
N	1.94471300	0.48843300	-0.56773800
C	-0.90944800	1.07768000	2.39255600

H	-0.67788500	1.26794000	3.45031000
H	-1.93337000	0.69960100	2.35015100
H	0.21105300	2.61123300	1.34051300
H	-1.17313100	3.20774400	2.25267200
C	2.48738000	-0.46663600	1.61352300
H	2.15280000	-1.48104400	1.35982000
H	3.35675000	-0.57232200	2.26948700
H	-0.74397800	-1.20514900	3.45605900
C	-1.65452200	-1.90560000	1.61330100
H	-2.36133400	-1.10735000	1.35159800
H	-2.18689200	-2.60077500	2.26952800

^{qnt}7

E (SMD/B3LYP-D3/BS1) = -2792.00033919 au
G (SMD/B3LYP-D3/BS1) = -2791.133698 au
E (SMD/B3LYP-D3/BS2//SMD/B3LYP-D3/BS1) = -5467.75658563 au
charge = 2 spin multiplicity = 3

Ni	2.06689300	0.37460500	-0.28673900
N	3.86045300	0.85674000	-1.39421200
C	4.06081600	2.33253600	-1.56966300
H	4.63375200	2.71175000	-0.72316200
C	5.04670400	0.26698500	-0.71087600
H	5.96004100	0.60497100	-1.21914200
H	4.99449600	-0.81724300	-0.80735400
C	3.78142500	-1.21621400	-2.93607800
C	2.25061900	3.43481600	-0.27984800
C	2.15642900	4.73533900	0.22113300
C	1.85076700	4.92806200	1.56784300
H	2.34510200	5.57669600	-0.43768000
C	1.70872200	2.55104200	1.80394400
C	1.63703600	3.81395200	2.38072100
H	1.78530400	5.93193000	1.97716000
H	1.50892900	1.65369900	2.37691700
H	1.39422400	3.91519600	3.43272000
C	4.27299300	-0.22829000	1.70231700
C	4.81247600	-0.81452600	2.85204500
C	2.14921800	-1.02923700	2.26915700
C	3.98583100	-1.51945000	3.72370500
H	5.87306400	-0.70715700	3.05537000
C	2.62548700	-1.62122700	3.43250200
H	1.09502600	-1.06525900	1.99738900
H	4.39721600	-1.97739900	4.61864800
H	1.93876400	-2.15020800	4.08278700
C	2.84045200	-2.11879900	-2.17877700
C	2.79860200	-3.47032200	-2.55187300
C	1.93803200	-4.34103200	-1.89983700
H	3.44209000	-3.81400000	-3.35617100

C	1.19453000	-2.48206400	-0.58138000
C	1.11399900	-3.83168200	-0.89526100
H	1.89649800	-5.39054800	-2.17694600
H	0.52189000	-2.03952100	0.15044900
H	0.40978900	-4.46155500	-0.36820300
N	2.05737000	-1.63715800	-1.19424200
N	2.01388200	2.36715800	0.50910800
N	2.95811900	-0.35914000	1.42101500
C	3.65025200	0.28985600	-2.76661500
H	4.38051500	0.74392900	-3.44885500
H	2.65568000	0.61323300	-3.09058700
H	4.81169100	-1.53771400	-2.74399000
H	3.62530000	-1.40571100	-4.00561600
C	5.13671700	0.60658900	0.78630600
H	4.89436400	1.66209700	0.95964700
H	6.17931300	0.47924400	1.09092400
H	4.67077200	2.49615200	-2.46575400
C	2.74523400	3.12754700	-1.66681700
H	1.99582100	2.55485000	-2.22569900
H	2.92347200	4.05697700	-2.21395500
O	0.14200800	0.39677000	0.03179100
Ni	-1.89966000	0.01318300	0.02281300
N	-4.08205000	-0.37213900	-0.04104000
C	-4.83187900	0.08193700	1.17672600
H	-5.25992300	1.06542200	0.98369000
C	-4.70878800	0.22654500	-1.25309900
H	-5.77691900	-0.03638700	-1.26515900
H	-4.24809400	-0.22696300	-2.13101900
C	-3.85713300	-2.58189700	-1.38814700
C	-3.15970100	1.42038800	2.47995800
C	-3.38032900	2.43442400	3.41776400
C	-2.64655800	3.61611900	3.33847000
H	-4.13107100	2.29313500	4.18884400
C	-1.52408200	2.69013800	1.43908400
C	-1.70086400	3.75048600	2.32270600
H	-2.81195400	4.41694600	4.05360900
H	-0.78722700	2.73483500	0.64847500
H	-1.10069200	4.64681500	2.21722000
C	-3.26813800	2.25519700	-1.94122000
C	-3.27803800	3.30611500	-2.87009600
C	-0.95592600	2.23991900	-1.95824400
C	-2.07845200	3.83342200	-3.33748400
H	-4.22872000	3.70401200	-3.21072100
C	-0.88368500	3.29145100	-2.86310000
H	-0.05832700	1.78605500	-1.55594000
H	-2.07638500	4.65090900	-4.05296600
H	0.08304100	3.66763300	-3.18088100

C	-2.54777900	-2.33532200	-2.09931100
C	-2.24275800	-3.16716000	-3.18938300
C	-1.12507200	-2.90987600	-3.97221600
H	-2.90414800	-3.99722700	-3.41890900
C	-0.66571100	-1.06356800	-2.52907600
C	-0.32540700	-1.81374600	-3.64526300
H	-0.88521200	-3.54464800	-4.82042100
H	-0.04370400	-0.24658300	-2.19852100
H	0.55885100	-1.55901000	-4.21928600
N	-1.74516200	-1.30944700	-1.75723900
N	-2.23565800	1.55421700	1.50562700
N	-2.11127900	1.71968400	-1.50128600
C	-4.20002900	-1.86390500	-0.08507800
H	-5.24223900	-2.13851300	0.13324900
H	-3.59077800	-2.24734700	0.72691500
H	-4.65208500	-2.40239700	-2.12334000
H	-3.92348200	-3.65312300	-1.16025200
C	-4.57138100	1.74938400	-1.37677400
H	-4.73068500	2.23957600	-0.40887300
H	-5.38277900	2.10014000	-2.02195900
H	-5.67644300	-0.59841800	1.34047100
C	-3.99111700	0.17001200	2.45708800
H	-3.34192800	-0.70210700	2.53933800
H	-4.67080600	0.16581700	3.31465300
O	-1.17725700	-1.32252000	1.37738700
C	-0.90896300	-2.49743300	3.46123000
C	-1.57479800	-2.44389500	2.10621600
C	-1.45185800	-3.75813200	1.37170200
C	-0.91122700	-4.88244000	1.93864700
C	-0.38034400	-4.85993600	3.25550100
C	-0.37715500	-3.64367700	3.98981800
H	-2.66599200	-2.36614100	2.35224800
H	-1.82712700	-3.77501500	0.35209900
H	-0.85969400	-5.80724300	1.36604400
H	0.04947900	-5.75936300	3.68661400
H	0.07514700	-3.62657400	4.98002600
H	-0.88846600	-1.56067300	4.01386600

^{t}8

E (SMD/B3LYP-D3/BS1) = -1204.55632694 au
G (SMD/B3LYP-D3/BS1) = -1204.182532 au
E (SMD/B3LYP-D3/BS2//SMD/B3LYP-D3/BS1) = -2542.36032864 au
charge = 2 spin multiplicity = 3

Ni	-0.06750200	-0.27767700	-0.28429600
N	0.04048400	-0.82158900	1.70862300
C	1.17866800	-1.78628600	1.91720000
H	2.05821800	-1.21825500	2.22399800

C	0.24670900	0.37235600	2.58963400
H	0.57442000	0.01702900	3.57379000
H	-0.71242200	0.87235000	2.72529800
C	-2.52935100	-0.75960400	1.73555900
C	2.37686500	-1.82910500	-0.30070200
C	3.66500300	-2.20938100	-0.67647100
C	4.39559500	-1.39587600	-1.54234600
H	4.08475800	-3.13057700	-0.28674100
C	2.54127000	0.11326400	-1.60039600
C	3.82693900	-0.21064100	-2.01294400
H	5.39973600	-1.68106800	-1.84155700
H	2.04254800	1.02031500	-1.92585300
H	4.36335200	0.45099700	-2.68409600
C	0.69776300	2.29150400	0.97188500
C	0.82274900	3.68063700	1.05033200
C	-0.45934600	2.49573300	-1.05146500
C	0.29514300	4.48256000	0.04065200
H	1.33261400	4.11900100	1.90165200
C	-0.36038100	3.88014100	-1.03436700
H	-0.95991400	1.97128400	-1.85867500
H	0.39140600	5.56282800	0.09392800
H	-0.78833400	4.46362500	-1.84205300
C	-2.95960600	-0.80357700	0.29303200
C	-4.28759100	-1.04889200	-0.06689100
C	-4.65373000	-1.04533100	-1.41067600
H	-5.02085900	-1.23861800	0.70969700
C	-2.37858400	-0.57480800	-1.96235300
C	-3.68019300	-0.80394700	-2.38240400
H	-5.68443600	-1.23203800	-1.69688200
H	-1.57654800	-0.38426000	-2.66951100
H	-3.91845500	-0.79548700	-3.44021300
N	-2.02848500	-0.56985900	-0.65697100
N	1.83861600	-0.67943300	-0.76853400
N	0.05821800	1.72354900	-0.07493700
C	-1.23741300	-1.51893900	2.07119300
H	-1.21575200	-1.71775100	3.15005100
H	-1.23507000	-2.48156800	1.55292800
H	-2.45116300	0.29110100	2.03942600
H	-3.32641100	-1.19188400	2.34700200
C	1.26180300	1.38243900	2.03423300
H	2.13797500	0.85959900	1.63073000
H	1.61884300	1.99647100	2.86538800
H	0.91412800	-2.44941100	2.74744300
C	1.53585000	-2.61596400	0.67292900
H	0.62121200	-2.97299600	0.17866000
H	2.08940300	-3.50211100	0.99481000

^{t}9

E (SMD/B3LYP-D3/BS1) = -1356.11360075 au

G (SMD/B3LYP-D3/BS1) = -1355.714878 au

E (SMD/B3LYP-D3/BS2//SMD/B3LYP-D3/BS1) = -2693.99578829 au

charge = 2 spin multiplicity = 3

Ni	-0.03379800	-0.24803900	-0.21948300
N	0.05046000	-0.61165100	1.83445900
C	1.35715300	-1.23554900	2.25202700
H	2.03230200	-0.44114000	2.57010800
C	-0.13554500	0.66314100	2.59438400
H	0.04368300	0.45814900	3.65730200
H	-1.17264800	0.97865300	2.48671300
C	-2.48488600	-1.13725100	1.90722000
C	2.73617200	-1.20698200	0.13019600
C	4.10185200	-1.31421200	-0.13932900
C	4.68524400	-0.47978800	-1.09108500
H	4.69348500	-2.04799300	0.39800800
C	2.54145200	0.52043500	-1.43255000
C	3.89131900	0.45845000	-1.75270100
H	5.74613200	-0.55742800	-1.30986700
H	1.87519500	1.23409700	-1.90568000
H	4.30340200	1.13067000	-2.49734900
C	0.25646800	2.54777600	0.91517400
C	0.21825000	3.94479800	0.87955300
C	-0.59947100	2.45417500	-1.25189800
C	-0.23598900	4.59811100	-0.26309200
H	0.54597400	4.50547400	1.74873500
C	-0.65354300	3.83729200	-1.35577600
H	-0.91170300	1.81877900	-2.07214800
H	-0.26660000	5.68298700	-0.29933400
H	-1.01529300	4.29779300	-2.26855700
C	-2.94571200	-0.92657000	0.48632100
C	-4.30501100	-1.07195100	0.17944400
C	-4.75263400	-0.87327300	-1.12106100
H	-4.99627600	-1.34655200	0.96954100
C	-2.49507200	-0.41880400	-1.74606400
C	-3.82453800	-0.54488900	-2.11247200
H	-5.80552800	-0.98411100	-1.36244200
H	-1.73220500	-0.18500100	-2.48109800
H	-4.11868400	-0.39564100	-3.14551200
N	-2.06110800	-0.58767300	-0.47473700
N	1.97989800	-0.29276900	-0.51824400
N	-0.15655400	1.82158900	-0.14673800
C	-1.04364800	-1.58756500	2.15442200
H	-0.96527700	-1.84490200	3.21854200
H	-0.83585000	-2.49536200	1.57978400
H	-2.71706800	-0.23440400	2.48386000

H	-3.11640900	-1.91897300	2.34377500
C	0.76945400	1.81158600	2.12552200
H	1.78240400	1.45048600	1.91089700
H	0.86494100	2.52252500	2.95062400
H	1.16565200	-1.86434100	3.12797800
C	2.05144400	-2.06325500	1.16493200
H	1.33504500	-2.73485300	0.68195100
H	2.79820500	-2.69712000	1.65109400
O	-0.09212600	-2.19199000	-1.37726800
O	1.04022900	-3.03687500	-1.69310200
H	1.10790900	-2.87897600	-2.65791800
H	-0.76468600	-2.83732200	-1.07272400

$^{t}TS_{9\text{-}10}$
E (SMD/B3LYP-D3/BS1) = -1356.07244117 au
G (SMD/B3LYP-D3/BS1) = -1355.673983 au
E (SMD/B3LYP-D3/BS2//SMD/B3LYP-D3/BS1) = -2693.95187452 au
negative eigenvalue of the frequency: 906i
charge = 2 spin multiplicity = 3

Ni	0.07086900	-0.31145400	-0.40420100
N	0.00968000	-1.25605300	1.38702300
C	1.36620600	-1.73619800	1.82064400
H	1.90065400	-0.89791200	2.26282600
C	-0.57603400	-0.35537800	2.42868000
H	-0.59154900	-0.91289500	3.37298300
H	-1.60428200	-0.13760900	2.14457100
C	-2.35382700	-2.25179600	1.08729300
C	2.93361200	-1.24095900	-0.06157200
C	4.31422800	-1.25585000	-0.27264500
C	4.92407100	-0.18201000	-0.91592800
H	4.89353300	-2.10396700	0.07665200
C	2.77090900	0.84449800	-1.08605900
C	4.13787000	0.89641800	-1.32364700
H	5.99628300	-0.18132600	-1.08709500
H	2.12484500	1.66008800	-1.38889100
H	4.56647500	1.76270500	-1.81539600
C	-0.05679000	2.00938200	1.56399200
C	-0.18895200	3.35783000	1.91498400
C	-0.20100900	2.57606400	-0.69096800
C	-0.32554800	4.32659200	0.92644500
H	-0.17884500	3.63215100	2.96459600
C	-0.32551900	3.92824800	-0.41079400
H	-0.18583800	2.22421300	-1.71659600
H	-0.42826800	5.37392700	1.19374200
H	-0.42181800	4.64059400	-1.22265000
C	-2.90169500	-1.27765200	0.07501300
C	-4.28961700	-1.23863000	-0.11713300

C	-4.84207500	-0.34781000	-1.02635900
H	-4.91533300	-1.91674500	0.45449400
C	-2.62429100	0.40248700	-1.50324400
C	-3.98579900	0.49316900	-1.74070700
H	-5.91609200	-0.31144900	-1.18160400
H	-1.93553800	1.02762800	-2.05293000
H	-4.35827400	1.20437400	-2.46980500
N	-2.08008800	-0.45558200	-0.60978200
N	2.17855100	-0.20249000	-0.47809500
N	-0.07670800	1.63408200	0.26745700
C	-0.85011600	-2.46719200	1.14701900
H	-0.65680800	-3.17459600	1.96074600
H	-0.49924200	-2.93905900	0.22534900
H	-2.74334200	-1.98001900	2.07510400
H	-2.78477700	-3.23703300	0.87231600
C	0.15565500	0.97087700	2.63528400
H	1.23347800	0.82179100	2.76584700
H	-0.20278600	1.38030800	3.58400000
H	1.20698000	-2.47968700	2.60825800
C	2.22882400	-2.32927000	0.69983100
H	1.63710100	-2.95049300	0.01892100
H	2.97228300	-2.98630300	1.15796100
O	0.09041500	-0.10583100	-2.38548800
O	0.22299200	-1.82123000	-1.80487300
H	-0.69054700	-2.04199400	-2.09564600
H	1.00946100	-0.11612700	-2.73424100

^{s}**10**

E (SMD/B3LYP-D3/BS1) = -1356.09190265 au

G (SMD/B3LYP-D3/BS1) = -1355.687979 au

E (SMD/B3LYP-D3/BS2//SMD/B3LYP-D3/BS1) = -2693.98020860 au

charge = 2 spin multiplicity = 1

Ni	-0.00487600	-0.32120700	-0.48144400
N	-0.14233000	-1.24471800	1.36271900
C	1.19840000	-1.67528700	1.87238100
H	1.73977000	-0.79438100	2.20980900
C	-0.79834200	-0.34901500	2.36753600
H	-0.94043200	-0.94257500	3.27816900
H	-1.78107500	-0.07374300	1.98818200
C	-2.43625800	-2.25171100	0.86079700
C	2.71936900	-1.41384200	-0.06474100
C	4.08872000	-1.50280700	-0.32972100
C	4.72188500	-0.53569500	-1.09995700
H	4.63966000	-2.33726400	0.09041900
C	2.60553300	0.55841500	-1.31666200
C	3.96372600	0.52645700	-1.58862000
H	5.78542600	-0.60384400	-1.30651800

H	1.99122300	1.34564100	-1.71652500
H	4.40026300	1.32229200	-2.18150300
C	0.10024300	1.93412800	1.62597500
C	0.24202100	3.27320700	2.01607300
C	0.19271600	2.59057500	-0.60509100
C	0.36709400	4.28125100	1.07244300
H	0.24435900	3.49935800	3.07728300
C	0.33228100	3.92722100	-0.27519200
H	0.15241100	2.27922800	-1.63965700
H	0.47671200	5.31681000	1.37908600
H	0.40802400	4.66254200	-1.06847700
C	-2.83522700	-1.18458800	-0.12568400
C	-4.19441500	-1.13904200	-0.47177500
C	-4.70535400	-0.09055700	-1.21878100
H	-4.83704300	-1.93619900	-0.11274800
C	-2.50048900	0.82682900	-1.27140600
C	-3.83799200	0.93213900	-1.60395200
H	-5.75778300	-0.06157800	-1.48301600
H	-1.79941200	1.57548700	-1.59509800
H	-4.17467600	1.79466800	-2.16801500
N	-2.00083100	-0.21706000	-0.56565000
N	1.99506200	-0.38812100	-0.57274900
N	0.09073600	1.59998500	0.31400300
C	-0.97358400	-2.48131200	1.19257000
H	-0.92975700	-3.02706200	2.14134400
H	-0.50613600	-3.08517000	0.42443700
H	-2.99270800	-2.02067500	1.77792800
H	-2.83665900	-3.20885900	0.51068700
C	-0.03497900	0.91102900	2.71872300
H	0.95768800	0.69426700	3.12716600
H	-0.58576400	1.37960000	3.54048400
H	1.02063300	-2.31498100	2.74277800
C	2.05798800	-2.40202300	0.84586500
H	1.48951500	-3.13422300	0.26853600
H	2.83491000	-2.94891100	1.38504500
O	-0.07680900	-1.96128700	-1.28502500
O	0.05155100	0.35241200	-2.20049800
H	-0.52584000	-0.24221000	-2.71588900
H	0.62680500	-1.93624300	-1.96224100

^{t}TS$_{9\text{-}11}$

E (SMD/B3LYP-D3/BS1) = -1588.35224231 au

G (SMD/B3LYP-D3/BS1) = -1587.860626 au

E (SMD/B3LYP-D3/BS2//SMD/B3LYP-D3/BS1) = -2926.31661574 au

negative eigenvalue of the frequency: 423i

charge = 2 spin multiplicity = 3

Ni	0.51458700	0.24481900	-0.38336500

N	1.76841300	1.97097800	-0.39430100
C	1.09168700	3.18637300	-0.97505400
H	0.72318600	3.80174900	-0.15362700
C	2.22621900	2.29120600	0.99012100
H	2.78651600	3.23500600	0.96563600
H	2.90983300	1.50813900	1.31676500
C	3.88667600	0.53270200	-0.82484300
C	-1.32767200	2.49591600	-1.18889700
C	-2.51600800	3.22452200	-1.28727900
C	-3.62787200	2.83895900	-0.53969200
H	-2.55760700	4.08770200	-1.94359900
C	-2.31464400	1.05720800	0.36095800
C	-3.52678900	1.73444400	0.30673000
H	-4.55693300	3.39660800	-0.61260600
H	-2.17712000	0.19137000	0.99779200
H	-4.36279600	1.39790300	0.91001100
C	0.65613700	1.06151500	2.58779200
C	0.48388200	0.89140100	3.96541600
C	0.05407300	-1.15723200	2.22284400
C	0.07608200	-0.34179500	4.46761400
H	0.67369600	1.72726800	4.63077400
C	-0.14227400	-1.39349200	3.57712100
H	-0.10354000	-1.93807800	1.49148400
H	-0.06240600	-0.48113300	5.53573100
H	-0.45576700	-2.37509500	3.91588300
C	3.40985500	-0.89806800	-0.74474100
C	4.35529300	-1.92927700	-0.84707500
C	3.95205400	-3.25680100	-0.77253100
H	5.40040800	-1.67355300	-0.99062400
C	1.71153700	-2.47310600	-0.52295100
C	2.59304600	-3.53854200	-0.61421800
H	4.68071500	-4.05839800	-0.84994900
H	0.64569900	-2.63603300	-0.42579600
H	2.22079600	-4.55629000	-0.56818000
N	2.10381300	-1.18000800	-0.56744200
N	-1.24737700	1.42031100	-0.37257100
N	0.43690600	0.03790300	1.73364200
C	2.92026500	1.62493000	-1.28885200
H	3.52340600	2.53061700	-1.43558200
H	2.48813000	1.35260300	-2.25639200
H	4.34208100	0.80064500	0.13577500
H	4.71561300	0.55738900	-1.54152800
C	1.08506500	2.38873500	2.01618500
H	0.20802900	2.88131600	1.58004800
H	1.42053600	3.02832700	2.83724200
H	1.84876900	3.78004500	-1.49877800
C	-0.07677000	2.89841800	-1.92795000

H	0.20100700	2.11818300	-2.64312800
H	-0.27815900	3.81173400	-2.49545100
O	0.12317500	-0.10072800	-2.35700200
C	-3.62507000	-2.18166700	-1.75911500
C	-2.41720300	-2.83973300	-1.33792400
C	-2.20646900	-3.03933300	0.06816900
C	-3.08544800	-2.50906900	0.99343000
C	-4.22612900	-1.81708000	0.55023500
C	-4.49142800	-1.65113900	-0.82288900
H	-3.81676500	-2.07505400	-2.82239000
H	-1.92946900	-3.51553400	-2.03148500
H	-1.33290900	-3.59884000	0.38647300
H	-2.90341900	-2.62989100	2.05633700
H	-4.91470100	-1.40019000	1.27942400
H	-5.38206800	-1.11793400	-1.13915300
H	0.73559700	-0.79036000	-2.67743000
O	-1.15602300	-1.45655400	-1.67217500
H	-1.77685600	-0.70116500	-1.69696800

11

E (SMD/B3LYP-D3/BS1) = -307.851134765 au
G (SMD/B3LYP-D3/BS1) = -307.766028 au
E (SMD/B3LYP-D3/BS2//SMD/B3LYP-D3/BS1) = -307.979142371 au
charge = 1 spin multiplicity = 1

C	0.20268000	-1.25778200	0.04733500
C	0.95537900	-0.00000100	0.24180000
C	0.20268300	1.25778100	0.04733700
C	-1.16658500	1.23975400	-0.03529700
C	-1.83919000	0.00000200	-0.07967200
C	-1.16658800	-1.23975300	-0.03529800
H	0.77110900	-2.18283400	0.05910900
H	0.95127500	-0.00000400	1.37184800
H	0.77111400	2.18283200	0.05911400
H	-1.73163300	2.16248000	-0.10847200
H	-2.92196500	0.00000300	-0.17120600
H	-1.73163800	-2.16247700	-0.10847200
O	2.30331500	-0.00000400	-0.13828700
H	2.33494800	0.00002400	-1.11285600

^{t}12

E (SMD/B3LYP-D3/BS1) = -1280.51983217 au
G (SMD/B3LYP-D3/BS1) = -1280.138808 au
E (SMD/B3LYP-D3/BS2//SMD/B3LYP-D3/BS1) = -2618.36599333 au
charge = 1 spin multiplicity = 3

Ni	0.00343100	-0.37682500	-0.56220900
N	0.06869900	-1.28404900	1.38198300
C	1.36451300	-2.01683900	1.59585700

H	2.04391800	-1.36707600	2.14872400
C	-0.11939800	-0.28472500	2.46989700
H	0.03311700	-0.77677000	3.44065000
H	-1.15007900	0.06807500	2.43643600
C	-2.47294100	-1.82212800	1.41423000
C	2.84205700	-1.35297100	-0.33514600
C	4.21888000	-1.42412000	-0.56514600
C	4.87797100	-0.33094600	-1.12621600
H	4.75984800	-2.32756900	-0.30204500
C	2.77898400	0.81472600	-1.17738200
C	4.14577500	0.81543000	-1.43853900
H	5.94720900	-0.37227000	-1.31344100
H	2.15938400	1.67772900	-1.40018900
H	4.61644300	1.69054300	-1.87422200
C	0.32785500	2.00135500	1.40157500
C	0.32147700	3.34992100	1.77591300
C	-0.45606000	2.56723700	-0.71319300
C	-0.08285900	4.32178500	0.86462200
H	0.63559500	3.62375300	2.77805900
C	-0.47809500	3.92351900	-0.41293600
H	-0.74647000	2.20801700	-1.69381500
H	-0.08916000	5.37070500	1.14621700
H	-0.79670800	4.64021500	-1.16223800
C	-3.02082300	-1.06344900	0.22930000
C	-4.41242400	-0.98977100	0.06148000
C	-4.94618500	-0.30595600	-1.02363600
H	-5.05870200	-1.47786700	0.78471400
C	-2.70934200	0.16640000	-1.71382900
C	-4.07243200	0.28200400	-1.94220900
H	-6.02180600	-0.24260800	-1.16060600
H	-1.98924800	0.58656400	-2.40707000
H	-4.43458400	0.81249600	-2.81653600
N	-2.18628200	-0.47262800	-0.64490700
N	2.14493300	-0.23987600	-0.64210400
N	-0.06822600	1.62361200	0.16693900
C	-1.02376700	-2.30991800	1.38447500
H	-0.89729800	-2.94623500	2.27152400
H	-0.86171700	-2.93713400	0.50272900
H	-2.66856200	-1.24247600	2.32413500
H	-3.08085500	-2.72779100	1.52847600
C	0.80452700	0.93813900	2.35921700
H	1.81559900	0.63439100	2.06250300
H	0.89669200	1.38318300	3.35416800
H	1.16855600	-2.88487500	2.23590700
C	2.06616600	-2.47482500	0.30605800
H	1.32883400	-2.85860000	-0.40861700
H	2.74855900	-3.29276400	0.55581200

O	0.11104900	-1.40006600	-2.15292200
H	-0.78017400	-1.70863500	-2.38577700

$^{qnt}TS_{9-13}$

E (SMD/B3LYP-D3/BS1) = -2560.65610907 au
G (SMD/B3LYP-D3/BS1) = -2559.851949 au
E (SMD/B3LYP-D3/BS2//SMD/B3LYP-D3/BS1) = -5236.33525414 au
negative eigenvalue of the frequency: 539i
charge = 4 spin multiplicity = 5

Ni	-2.43010100	-0.33592300	-0.26783500
N	-3.92082200	-1.69504700	-0.87703900
C	-3.44449800	-3.12696000	-0.92245800
H	-3.77181400	-3.62954800	-0.01191200
C	-5.08895700	-1.61089100	0.05489700
H	-5.80015200	-2.40187300	-0.21369400
H	-5.58415200	-0.65282200	-0.09925500
C	-4.95210700	0.07548200	-2.47314200
C	-1.19115000	-2.99925500	0.21559700
C	-0.44366200	-3.96674700	0.89377600
C	0.20852300	-3.63247500	2.07966000
H	-0.37601500	-4.96847500	0.48356100
C	-0.68511600	-1.42510400	1.86463300
C	0.09300700	-2.33167000	2.57403200
H	0.79933900	-4.37452900	2.60796400
H	-0.83618400	-0.41095000	2.21388300
H	0.58463100	-2.02341200	3.49017200
C	-4.25850400	-0.43564400	2.16418900
C	-4.75811400	-0.00580500	3.39757000
C	-2.91914600	1.46844200	2.02730700
C	-4.30852300	1.19115000	3.94972800
H	-5.49680300	-0.61320200	3.91020500
C	-3.36698400	1.94765000	3.25023100
H	-2.18581500	2.01524600	1.44806900
H	-4.69090600	1.53083000	4.90757600
H	-2.98547700	2.88673300	3.63567800
C	-4.10733400	1.30642300	-2.26161300
C	-4.40065400	2.48140400	-2.96733700
C	-3.63885200	3.62654200	-2.76465800
H	-5.22476700	2.47978500	-3.67351400
C	-2.33425500	2.38878200	-1.19969100
C	-2.57392700	3.58159500	-1.86029700
H	-3.86382800	4.53800400	-3.31034300
H	-1.51277300	2.28573800	-0.50077600
H	-1.94154900	4.44246900	-1.67375700
N	-3.08562200	1.28032100	-1.38297300
N	-1.30663500	-1.74794800	0.71540200
N	-3.34785600	0.30520100	1.49656300

C	-4.31429700	-1.30230900	-2.27184100
H	-5.03679200	-2.04129900	-2.64083500
H	-3.41135200	-1.38877500	-2.88479800
H	-5.86048700	0.17409600	-1.86758800
H	-5.30271400	0.08944900	-3.51066600
C	-4.71481800	-1.72955800	1.54085500
H	-3.93475700	-2.48614900	1.68857000
H	-5.59460900	-2.08563400	2.08350200
H	-3.94578700	-3.62513400	-1.75828900
C	-1.92820200	-3.30950000	-1.06081600
H	-1.54834100	-2.68978400	-1.87897600
H	-1.74346700	-4.35292900	-1.33140600
O	-0.98813400	-0.48150000	-1.93767700
O	0.75224000	-0.04475500	-1.43682300
H	1.04256600	-0.68094900	-2.11849500
H	-1.08156600	0.34443100	-2.46170600
Ni	2.34450500	0.23568000	-0.30442600
N	3.49890500	1.59060600	-1.45337800
C	2.82608300	2.92994400	-1.64385400
H	3.29775800	3.64946600	-0.97423800
C	4.83200500	1.81345000	-0.81617600
H	5.34638800	2.60947500	-1.36716100
H	5.42264900	0.90415000	-0.91514500
C	4.43036900	-0.35496500	-2.86451600
C	0.99193400	2.78787500	0.06626200
C	0.34493800	3.79364200	0.78917000
C	0.08413200	3.62111900	2.14611400
H	0.05413600	4.70279900	0.27420300
C	1.11766500	1.46995000	2.00130000
C	0.47141100	2.43145900	2.76432000
H	-0.41728300	4.40082200	2.71090800
H	1.44676300	0.53712200	2.43890500
H	0.28078200	2.24291600	3.81469800
C	4.57030400	0.96333900	1.55196700
C	5.33550300	0.80171600	2.71101900
C	3.47733800	-1.05790600	1.99057700
C	5.15212600	-0.31365300	3.52186700
H	6.06830600	1.56052100	2.96285600
C	4.20100900	-1.26596400	3.15397400
H	2.71724800	-1.76112600	1.68073600
H	5.74189000	-0.43894400	4.42466600
H	4.01419200	-2.15292100	3.74897700
C	3.84399100	-1.56302500	-2.18594900
C	4.22641400	-2.84328400	-2.60888800
C	3.66119600	-3.97411800	-2.03465900
H	4.96102900	-2.92733300	-3.40314200
C	2.34821800	-2.53297000	-0.66345400

C	2.68352400	-3.81658100	-1.04795200
H	3.95898100	-4.96417800	-2.36562900
H	1.58190000	-2.34959400	0.07796700
H	2.18139100	-4.66016000	-0.58838200
N	2.93917100	-1.43531300	-1.19385900
N	1.37505700	1.64830200	0.68933500
N	3.65591500	0.02856200	1.20759500
C	3.65244000	0.95698200	-2.80339400
H	4.17600900	1.66854700	-3.45352400
H	2.64401700	0.82231800	-3.20485900
H	5.46028600	-0.22197400	-2.51626300
H	4.52285900	-0.59894100	-3.92854600
C	4.74259800	2.16880800	0.66999200
H	3.92730100	2.87539900	0.86579400
H	5.66588300	2.67916500	0.95569100
H	3.02454300	3.26484900	-2.66570000
C	1.31726300	2.94514700	-1.39265700
H	0.82096600	2.16572000	-1.97547300
H	0.93528600	3.90952900	-1.73684500

[d]13

E (SMD/B3LYP-D3/BS1) = -1280.33021541 au

G (SMD/B3LYP-D3/BS1) = -1279.939910 au

E (SMD/B3LYP-D3/BS2//SMD/B3LYP-D3/BS1) = -2618.17772787 au

charge = 2 spin multiplicity = 2

Ni	-0.02787300	-0.34945800	-0.47990800
N	0.13670300	-1.31135600	1.42789500
C	1.43096900	-2.06998800	1.57814400
H	2.11805700	-1.46378800	2.16819400
C	0.02845800	-0.34126800	2.55534000
H	0.25952200	-0.87174900	3.48703600
H	-0.99845300	0.01420100	2.62046000
C	-2.39762700	-1.71842600	1.43441900
C	2.70803500	-1.25984500	-0.43090200
C	4.06449600	-1.19826000	-0.75856200
C	4.58053500	-0.07762300	-1.40327700
H	4.70111200	-2.03867800	-0.50381400
C	2.38885700	0.87478100	-1.35499900
C	3.72492700	0.97972300	-1.71149800
H	5.63384000	-0.03072400	-1.66214600
H	1.68651100	1.66790300	-1.57484400
H	4.07437400	1.87292200	-2.21714700
C	0.38437300	1.90481300	1.46092700
C	0.36759700	3.25373700	1.82821100
C	-0.59595800	2.45068500	-0.58667100
C	-0.14170000	4.21260100	0.96003700
H	0.76010900	3.53346500	2.79995700

C	-0.63281900	3.80109300	-0.27868300
H	-0.94871000	2.09787100	-1.54579700
H	-0.15439000	5.26042400	1.24345800
H	-1.03637500	4.50292300	-0.99978000
C	-2.84943900	-0.97246000	0.20771000
C	-4.22368900	-0.86757100	-0.04771400
C	-4.68399700	-0.24911400	-1.20119400
H	-4.91542100	-1.29623400	0.66992400
C	-2.40899500	0.11468500	-1.82158500
C	-3.75233200	0.23589800	-2.12249500
H	-5.74912000	-0.16944900	-1.39498300
H	-1.64698800	0.44286200	-2.51883100
H	-4.05183000	0.69123800	-3.05957700
N	-1.96853100	-0.43607600	-0.66278000
N	1.90145500	-0.21226500	-0.72326400
N	-0.10650400	1.51720000	0.26031400
C	-0.98723000	-2.29709400	1.45029800
H	-0.89559900	-2.89447300	2.36580400
H	-0.83845800	-2.96546400	0.59867800
H	-2.58402800	-1.09537600	2.31555700
H	-3.07184300	-2.57434900	1.54835100
C	0.94807400	0.86891800	2.39012600
H	1.94136700	0.57093300	2.03371400
H	1.09586600	1.32544400	3.37187000
H	1.22145700	-2.97464600	2.15708700
C	2.11944800	-2.45266600	0.26980700
H	1.41824100	-2.95843000	-0.39778000
H	2.92521100	-3.15091500	0.51143800
O	0.05969000	-1.70469300	-1.68351800
H	-0.81529200	-2.13176500	-1.73103600

^{t}14

E (SMD/B3LYP-D3/BS1) = -1355.65281524 au
G (SMD/B3LYP-D3/BS1) = -1355.268626 au
E (SMD/B3LYP-D3/BS2//SMD/B3LYP-D3/BS1) = -2693.53354912 au
charge = 1 spin multiplicity = 3

Ni	-0.05013400	-0.37817600	-0.38328800
N	0.02515400	-0.88763700	1.71311900
C	1.29370100	-1.62731500	2.04428600
H	1.99467200	-0.92725000	2.50152400
C	-0.08198300	0.31572400	2.58524500
H	0.09942300	0.01933700	3.62763900
H	-1.10138600	0.69608500	2.52459400
C	-2.53768100	-1.28882000	1.83665600
C	2.76404600	-1.30234800	0.02438400
C	4.14543300	-1.39918900	-0.15997900
C	4.80837100	-0.42423300	-0.90439600

H	4.68666200	-2.23209700	0.27743200
C	2.70367000	0.67002200	-1.20854800
C	4.07486500	0.63449400	-1.44174200
H	5.88144600	-0.48881700	-1.06052200
H	2.08268400	1.46913500	-1.60065500
H	4.54784600	1.41632500	-2.02663800
C	0.36607900	2.33234900	1.07882800
C	0.39810600	3.72662500	1.19321400
C	-0.54801800	2.51051300	-1.05265200
C	-0.05409700	4.51980100	0.14183200
H	0.77845200	4.17603000	2.10484100
C	-0.53779500	3.89935900	-1.01053800
H	-0.90718800	1.98084300	-1.92772000
H	-0.03068400	5.60275100	0.22120900
H	-0.89852900	4.47104100	-1.85896500
C	-3.06564100	-0.88934600	0.47970200
C	-4.45030000	-0.92269500	0.25481600
C	-4.96235400	-0.55495900	-0.98372400
H	-5.10884300	-1.24337500	1.05616200
C	-2.71641700	-0.17355100	-1.70143100
C	-4.07299600	-0.17668900	-1.99228800
H	-6.03306100	-0.57543100	-1.16543500
H	-1.98160400	0.08199600	-2.45581000
H	-4.41726500	0.10234200	-2.98257800
N	-2.21822300	-0.50060300	-0.49017800
N	2.06737200	-0.27132300	-0.49540600
N	-0.11059300	1.74058500	-0.03776300
C	-1.11179000	-1.83414700	1.94838300
H	-1.01362000	-2.24064900	2.96480700
H	-0.97474700	-2.66147400	1.24552000
H	-2.68860200	-0.45375000	2.53091100
H	-3.18622600	-2.08678800	2.21644800
C	0.87502600	1.45267500	2.19343600
H	1.85332300	1.05140000	1.90381200
H	1.04456500	2.07506600	3.07673500
H	1.06435200	-2.38301200	2.80408600
C	1.98243100	-2.29562600	0.84389500
H	1.24185900	-2.78970100	0.20517400
H	2.66015600	-3.06638100	1.22349800
O	-0.19014300	-1.73769900	-1.72312300
O	1.06096200	-2.23928000	-2.30131500
H	0.86161500	-3.19190600	-2.32317200

^{t}TS$_{14\text{-}15}$

E (SMD/B3LYP-D3/BS1) = -1355.61935294 au

G (SMD/B3LYP-D3/BS1) = -1355.233372 au

E (SMD/B3LYP-D3/BS2//SMD/B3LYP-D3/BS1) = -2693.49912972 au

negative eigenvalue of the frequency: 549i

charge = 1 spin multiplicity = 3

Ni	0.04120300	-0.29291000	-0.47472800
N	-0.01158100	-1.30726600	1.33534000
C	1.32622300	-1.86681500	1.72241700
H	1.89824700	-1.08132200	2.21409100
C	-0.51305500	-0.41569300	2.42160000
H	-0.52011700	-0.98083400	3.36339100
H	-1.54171100	-0.14523800	2.18425100
C	-2.43631800	-2.18562900	1.08965100
C	2.92533200	-1.31812100	-0.14256600
C	4.29822000	-1.41175400	-0.38891500
C	4.96234400	-0.34747200	-0.99565000
H	4.83207100	-2.31113300	-0.09873400
C	2.87494700	0.81731100	-1.05592600
C	4.23808200	0.79700700	-1.33149700
H	6.02848800	-0.40693600	-1.19469900
H	2.26850000	1.68208200	-1.30220300
H	4.71119100	1.65558300	-1.79622600
C	0.05643900	1.95798600	1.59109200
C	-0.03284300	3.29757700	1.98845700
C	-0.12101900	2.59806500	-0.63980200
C	-0.16518900	4.30126400	1.03399000
H	0.00816000	3.53831300	3.04587700
C	-0.20226100	3.94476600	-0.31438800
H	-0.13417900	2.26222300	-1.67179600
H	-0.23451400	5.34200700	1.33638900
H	-0.29426600	4.68626500	-1.10071400
C	-2.98775200	-1.22475000	0.06310400
C	-4.36090700	-1.27787600	-0.22135100
C	-4.92185400	-0.37166000	-1.11228600
H	-4.97209500	-2.03174100	0.26562200
C	-2.73943600	0.56162900	-1.38914400
C	-4.08963600	0.57650000	-1.71068100
H	-5.98375300	-0.40442300	-1.33846300
H	-2.05731600	1.26206200	-1.85034000
H	-4.47100800	1.30736800	-2.41613800
N	-2.18899200	-0.31169900	-0.52160400
N	2.22990800	-0.21371300	-0.48355100
N	-0.00428400	1.62328500	0.28509600
C	-0.93695800	-2.47080400	1.12399700
H	-0.77499600	-3.18724100	1.93934400
H	-0.63487900	-2.94924600	0.18989600
H	-2.78333300	-1.86145700	2.07907200
H	-2.91586400	-3.15865000	0.92981400
C	0.27851300	0.88013200	2.62170400
H	1.35572100	0.68559100	2.68341500

H	-0.00616000	1.27985300	3.59961200
H	1.15926400	-2.65707600	2.46399300
C	2.16202400	-2.41226000	0.55562000
H	1.52561700	-2.93043000	-0.16935900
H	2.86859400	-3.14696300	0.95229000
O	0.01643900	0.07120100	-2.37659400
O	0.07392700	-1.73343800	-1.63347600
H	0.92696900	-0.05995700	-2.69895300

^{t}15

E (SMD/B3LYP-D3/BS1) = -1355.62993494 au
G (SMD/B3LYP-D3/BS1) = -1355.243553 au
E (SMD/B3LYP-D3/BS2//SMD/B3LYP-D3/BS1) = -2693.51204212 au
charge = 1 spin multiplicity = 3

Ni	0.03638200	-0.33043400	-0.55103300
N	0.10518600	-1.33644200	1.28590100
C	1.41261800	-2.07748400	1.50879400
H	2.04996800	-1.45768200	2.13911500
C	-0.07190300	-0.39451500	2.43070800
H	0.07867700	-0.95650800	3.36073900
H	-1.09830800	-0.03111500	2.42244600
C	-2.43122400	-1.89562200	1.40504900
C	2.93470600	-1.28835500	-0.31929100
C	4.31415400	-1.27974200	-0.54633500
C	4.91910900	-0.12349300	-1.03511300
H	4.89621900	-2.17048800	-0.33292600
C	2.76406800	0.91717400	-1.04310400
C	4.13092500	1.00228400	-1.28241700
H	5.98983300	-0.09947500	-1.21626800
H	2.09490300	1.74598600	-1.23851400
H	4.55967500	1.92526300	-1.65828900
C	0.33886500	1.90238000	1.49593700
C	0.28462800	3.22547700	1.94473000
C	-0.60725900	2.53119900	-0.53290600
C	-0.23614000	4.21896700	1.12229200
H	0.64876700	3.45686700	2.94024100
C	-0.69765400	3.86054100	-0.14342000
H	-0.91422400	2.21005600	-1.51713500
H	-0.28432100	5.24874300	1.46358200
H	-1.11700200	4.59059200	-0.82736700
C	-3.02535500	-1.09560000	0.27371100
C	-4.42314300	-1.01712000	0.17349800
C	-5.00593800	-0.31267700	-0.87232000
H	-5.03399900	-1.52120400	0.91638000
C	-2.79886800	0.17581000	-1.65251300
C	-4.17221700	0.28918600	-1.81747000
H	-6.08672200	-0.24674700	-0.95892300

H	-2.09433200	0.60284400	-2.35542400
H	-4.57202400	0.83254900	-2.66738700
N	-2.23004400	-0.48022700	-0.61928100
N	2.18561700	-0.19815100	-0.57048800
N	-0.10237000	1.56623600	0.26328000
C	-0.98685500	-2.37306800	1.29035400
H	-0.81212900	-3.02660100	2.15283900
H	-0.84836500	-2.95405600	0.37839100
H	-2.59818500	-1.36023700	2.34706200
H	-3.02582700	-2.81245000	1.50069400
C	0.86581700	0.81442400	2.38596000
H	1.87144600	0.53114400	2.05650200
H	0.96877400	1.20751900	3.40065300
H	1.17890700	-2.97618900	2.08536900
C	2.21564700	-2.47218000	0.26678600
H	1.55828200	-2.92930800	-0.48126300
H	2.94634000	-3.22774600	0.57111900
O	0.05409200	0.47102500	-2.21355700
O	-0.00227900	-1.91047700	-1.42536000
H	0.15784300	-0.31939800	-2.77485600

^{t}TS$_{15}$

E (SMD/B3LYP-D3/BS1) = -1587.87729845 au

G (SMD/B3LYP-D3/BS1) = -1587.399876 au

E (SMD/B3LYP-D3/BS2//SMD/B3LYP-D3/BS1) = -2925.84785750 au

negative eigenvalue of the frequency: 357i

charge = 1 spin multiplicity = 3

Ni	-0.06468600	-0.14876100	-0.41058300
N	-0.95429600	-2.12806100	-1.00266200
C	0.05550100	-3.07100000	-1.60437800
H	0.39824800	-3.75301300	-0.82595600
C	-1.58880800	-2.79084600	0.16881100
H	-1.96805900	-3.77259000	-0.14729000
H	-2.44294300	-2.19036700	0.48106400
C	-3.30480000	-1.20210300	-1.63472200
C	2.32433500	-2.01756700	-1.24724100
C	3.62991600	-2.52148100	-1.25553500
C	4.52302100	-2.13153400	-0.25844100
H	3.93189300	-3.21283200	-2.03584700
C	2.78242000	-0.78500700	0.67760900
C	4.09278000	-1.24929200	0.73484700
H	5.53951300	-2.51463600	-0.25419000
H	2.38516500	-0.08089000	1.40008300
H	4.75283600	-0.92299700	1.53177900
C	-0.62578900	-1.77368000	2.29774900
C	-0.82683700	-1.93330900	3.67393500
C	-0.34261300	0.51630600	2.58708200

C	-0.78335000	-0.82611900	4.51623000
H	-1.01460200	-2.92680000	4.06793600
C	-0.53472800	0.42859700	3.96038100
H	-0.11958900	1.45802500	2.09961100
H	-0.94050700	-0.94112100	5.58481600
H	-0.48738100	1.32484900	4.56985400
C	-3.37170900	0.16676500	-0.99334900
C	-4.61622100	0.81746300	-0.94901300
C	-4.72174500	2.07360700	-0.36425900
H	-5.48558300	0.32900700	-1.37925700
C	-2.37510100	1.96975000	0.07719200
C	-3.57097300	2.67038400	0.15804700
H	-5.68001400	2.58382800	-0.32497300
H	-1.43940800	2.38288500	0.43655300
H	-3.59566500	3.65594400	0.61165500
N	-2.27568400	0.74191800	-0.47099400
N	1.92951500	-1.16025400	-0.28663800
N	-0.39115200	-0.55367600	1.77141100
C	-1.96138000	-1.79572100	-2.06412500
H	-2.20538800	-2.72325600	-2.59822600
H	-1.45078000	-1.11925000	-2.75195600
H	-3.85102000	-1.90796500	-0.99695000
H	-3.89641700	-1.15244900	-2.55759500
C	-0.65485600	-2.96329400	1.37714900
H	0.37043700	-3.17722800	1.05596800
H	-0.98877600	-3.83513400	1.94614700
H	-0.46591400	-3.68519000	-2.34682500
C	1.28868500	-2.43129700	-2.26130100
H	0.98764000	-1.56351400	-2.85360600
H	1.72669500	-3.17275700	-2.93668600
O	0.59995800	1.44364300	0.13981600
O	0.13943200	0.32226700	-2.22847800
H	-0.33893300	1.16706000	-2.29382100
C	1.14317800	3.46263900	-1.42209100
C	1.97714400	2.39640700	-0.98518300
C	2.96430300	2.66278700	0.00381600
C	3.02928700	3.90835200	0.60928200
C	2.15095700	4.93199300	0.20998500
C	1.21537400	4.70792700	-0.81169000
H	0.42512800	3.27982700	-2.21576000
H	2.03331600	1.49723000	-1.58734900
H	3.64215700	1.86801100	0.29879100
H	3.76527600	4.10151000	1.38517300
H	2.20697300	5.90677700	0.68724100
H	0.55516000	5.51122000	-1.12730200

$^{S}TS_{15}$

E (SMD/B3LYP-D3/BS1) = -1587.85680787 au
G (SMD/B3LYP-D3/BS1) = -1587.375403 au
E (SMD/B3LYP-D3/BS2//SMD/B3LYP-D3/BS1) = -2925.83323727 au
negative eigenvalue of the frequency: 398i
charge = 1 spin multiplicity = 1

Ni	-0.27182600	0.11568900	-0.32673400
N	0.04258500	0.22492200	1.70402300
C	-0.00805500	1.62606800	2.23419700
H	-1.04826200	1.90476700	2.39511700
C	-0.91708600	-0.62797100	2.47978100
H	-0.61965800	-0.57040500	3.53489600
H	-0.78465300	-1.65813200	2.15058800
C	1.68223100	-1.74087400	1.78359000
C	-0.33318900	2.99514700	0.21221600
C	-0.69318300	4.31712700	-0.06481300
C	-1.70072800	4.58658800	-0.98504200
H	-0.18630800	5.11803700	0.46305100
C	-1.92799000	2.22495500	-1.30570600
C	-2.34794500	3.51458500	-1.59949300
H	-1.98755200	5.61081300	-1.20450700
H	-2.36982100	1.36720100	-1.78559300
H	-3.15847500	3.66349200	-2.30474100
C	-3.09386500	-0.80528100	1.13115400
C	-4.37934100	-1.35673000	1.21513800
C	-3.07151400	-1.12412100	-1.16151200
C	-5.01555000	-1.79796700	0.05821300
H	-4.86528900	-1.43316100	2.18292000
C	-4.34841000	-1.67572200	-1.16144200
H	-2.51130700	-0.99247700	-2.08027100
H	-6.01186300	-2.22808200	0.10734800
H	-4.80058100	-1.99914500	-2.09340200
C	1.25323300	-2.41172500	0.50576900
C	1.73829000	-3.71037700	0.28401600
C	1.33652700	-4.43547300	-0.82703100
H	2.43112600	-4.13171900	1.00573300
C	0.01194800	-2.54378900	-1.45030300
C	0.44073300	-3.83494600	-1.71222700
H	1.71108000	-5.44014100	-0.99985700
H	-0.62817600	-2.01079900	-2.13535900
H	0.08312500	-4.34518800	-2.60040600
N	0.40138800	-1.83194300	-0.36674300
N	-0.93399400	1.96953400	-0.43088200
N	-2.45030900	-0.70140800	-0.04567900
C	1.44749900	-0.25202300	1.93792300
H	1.73135800	0.01703600	2.96245500
H	2.06924200	0.28648700	1.22799300
H	1.23071400	-2.29693000	2.61453500

H	2.76247500	-1.88924400	1.89072300
C	-2.40554500	-0.28176600	2.36538900
H	-2.57655800	0.79875500	2.42822700
H	-2.89754400	-0.70876300	3.24495700
H	0.48603900	1.63269000	3.21178600
C	0.63367300	2.65749100	1.30501100
H	1.55971100	2.27417100	0.87195100
H	0.85928300	3.55850200	1.88191900
O	1.48579900	0.68425700	-0.79232800
O	-0.53754700	0.03835900	-2.13819600
H	0.36218600	0.34300300	-2.37470900
C	3.42655700	1.63575300	-1.16587200
C	3.28757700	0.29877400	-1.62145400
C	3.95399400	-0.74028200	-0.92488400
C	4.65606600	-0.46232000	0.23566600
C	4.75604600	0.86566300	0.70865500
C	4.15898100	1.90419100	0.00457700
H	3.01675300	2.44912700	-1.75258500
H	2.87647200	0.10012800	-2.60339400
H	3.89364000	-1.75771000	-1.30110200
H	5.15109900	-1.26403600	0.77791400
H	5.32134500	1.07405300	1.61336800
H	4.26208700	2.92986200	0.34907100

^{t}16

E (SMD/B3LYP-D3/BS1) = -1648.56786085 au
G (SMD/B3LYP-D3/BS1) = -1647.966578 au
E (SMD/B3LYP-D3/BS2//SMD/B3LYP-D3/BS1) = -2986.55909052 au
charge = 2 spin multiplicity = 3

Ni	-0.51657200	-0.07081100	-0.17760600
N	-1.41672000	-0.84086500	-1.88213200
C	-0.40120000	-1.43769400	-2.85468600
H	-0.48096000	-2.52320400	-2.78807600
C	-2.39840900	-1.92801800	-1.56815300
H	-2.66406600	-2.41822600	-2.51132400
H	-3.30373500	-1.47769700	-1.16583600
C	-3.26351800	0.98663300	-1.88526700
C	1.68921300	-1.74434900	-1.49390300
C	2.86026900	-2.50324500	-1.58487900
C	3.34412600	-3.15524100	-0.45241500
H	3.37307700	-2.57980700	-2.53805400
C	1.48451600	-2.26760600	0.76139700
C	2.63496900	-3.04700400	0.74540200
H	4.25358300	-3.74619000	-0.50550700
H	0.90238500	-2.15566800	1.66985100
H	2.96385300	-3.54602600	1.65046100
C	-2.02467500	-2.44575700	0.84173800

C	-2.64524000	-3.21633100	1.82897500
C	-1.86906600	-0.65060800	2.32172700
C	-2.87507300	-2.67925500	3.09117500
H	-2.95387900	-4.22751900	1.58594400
C	-2.49629100	-1.35908500	3.33535900
H	-1.54600400	0.36917100	2.47181700
H	-3.35699700	-3.27197500	3.86273900
H	-2.67672400	-0.87842800	4.29064800
C	-2.88740000	1.99354700	-0.83210900
C	-3.58743700	3.20393300	-0.74293100
C	-3.26113600	4.12292400	0.24974300
H	-4.37925100	3.41186500	-1.45541000
C	-1.57030500	2.59802200	0.98222100
C	-2.22663200	3.81362300	1.13268800
H	-3.79737300	5.06438600	0.32612700
H	-0.74081400	2.30838400	1.61436300
H	-1.92152800	4.49765200	1.91764500
N	-1.89783400	1.69936200	0.03245000
N	1.02833900	-1.62351800	-0.32585600
N	-1.61514000	-1.18379400	1.10631100
C	-2.11982100	0.27443100	-2.61221900
H	-2.53666100	-0.16401200	-3.52613900
H	-1.34649000	0.99059600	-2.88923400
H	-3.96670700	0.26911600	-1.44739800
H	-3.83171500	1.50609600	-2.66346500
C	-1.88041300	-2.95844700	-0.56274200
H	-0.84201300	-3.23329100	-0.76949300
H	-2.47642000	-3.86875000	-0.66602400
H	-0.72464400	-1.15656700	-3.85966400
C	1.07240800	-1.06453800	-2.68357100
H	1.18962900	0.02342400	-2.60214400
H	1.59387000	-1.37642000	-3.59387300
O	0.48965100	0.62027200	1.25403300
O	0.34613200	1.11258000	-1.21855500
H	0.57291800	-0.07835700	1.92444600
N	3.02021100	1.37706900	0.56914900
C	2.92623100	2.73412600	1.22390600
H	2.50660800	2.55743100	2.21476600
H	3.94285900	3.11610000	1.34385200
C	3.38793400	1.42924600	-0.89572600
H	3.38453900	0.39188100	-1.23474000
H	2.56769100	1.93772100	-1.39822200
C	3.93616600	0.43807300	1.31963500
H	3.97589400	-0.47555000	0.72256100
H	4.92866600	0.89295800	1.32534600
C	4.72497500	2.09450900	-1.18974000
H	5.56492100	1.57355000	-0.72072000

H	4.88169200	2.07026900	-2.27362200
H	4.74177600	3.14242000	-0.87506100
C	2.03901700	3.70196200	0.45094900
H	1.85125700	4.57821700	1.08041700
H	2.51000600	4.04648900	-0.47432600
H	1.07795800	3.24115200	0.20529300
C	3.48835100	0.11445900	2.73887600
H	4.19137700	-0.61721800	3.15144700
H	3.49634100	0.98919200	3.39495500
H	2.49277400	-0.33621000	2.76183200
H	2.03871200	0.98530900	0.64082600

$^{t}TS_{16-17}$
E (SMD/B3LYP-D3/BS1) = -1648.54865875 au
G (SMD/B3LYP-D3/BS1) = -1647.950473 au
E (SMD/B3LYP-D3/BS2//SMD/B3LYP-D3/BS1) = -2986.54513525 au
negative eigenvalue of the frequency: 137i
charge = 2 spin multiplicity = 3

Ni	-0.63131500	0.02644400	-0.21812400
N	-2.01601700	-0.89101300	-1.67602800
C	-1.30366800	-1.81025300	-2.63888000
H	-1.53991400	-2.84029100	-2.36559800
C	-3.09627000	-1.67602700	-1.01270000
H	-3.58541600	-2.30289200	-1.76779500
H	-3.85017700	-0.99476200	-0.62198300
C	-3.33338100	1.29834500	-1.63626000
C	0.85356700	-2.18861800	-1.43592500
C	1.74131700	-3.26787900	-1.45659500
C	2.25292200	-3.77422200	-0.26599100
H	2.01703400	-3.69968300	-2.41247900
C	0.96285700	-2.13019000	0.89822700
C	1.84582000	-3.19919000	0.93822000
H	2.94765900	-4.60852100	-0.27628000
H	0.61104700	-1.66826300	1.80763600
H	2.19781300	-3.56464200	1.89641100
C	-2.38843900	-1.74158200	1.39941200
C	-2.99155300	-2.15160600	2.59224700
C	-1.54271600	0.15282900	2.47992700
C	-2.86061100	-1.38742700	3.74669800
H	-3.56743200	-3.07063100	2.59523900
C	-2.12624700	-0.20395100	3.68554900
H	-0.93924000	1.04373600	2.39832900
H	-3.32894400	-1.70561000	4.67308500
H	-1.99717700	0.43735500	4.55034200
C	-2.49741600	2.27963100	-0.86452400
C	-2.87210800	3.62809100	-0.84911000
C	-2.13467900	4.56070900	-0.12843000

H	-3.74794700	3.92906800	-1.41409000
C	-0.67546400	2.78045300	0.52277700
C	-1.01038600	4.12520100	0.56998700
H	-2.42733500	5.60632200	-0.12203800
H	0.19170400	2.36883600	1.03457900
H	-0.38737400	4.80724400	1.13835700
N	-1.41396100	1.87479100	-0.16235200
N	0.48702300	-1.62837400	-0.25925300
N	-1.66023000	-0.59806700	1.36128500
C	-2.59152700	0.24302600	-2.45615500
H	-3.29677100	-0.17245800	-3.18807700
H	-1.76476600	0.70567500	-2.99836100
H	-4.04714900	0.83747100	-0.94456000
H	-3.94248200	1.86949500	-2.34247400
C	-2.58126400	-2.54067900	0.13943700
H	-1.65162500	-3.04967800	-0.13516800
H	-3.31794700	-3.32280700	0.33857500
H	-1.71997300	-1.64102300	-3.63678500
C	0.21888900	-1.67378700	-2.69491900
H	0.50306200	-0.63196500	-2.87726100
H	0.57706300	-2.26422700	-3.54299000
O	1.19281900	0.81213600	1.37431100
O	0.31673800	0.79249100	-1.49782800
H	1.22385900	0.24294000	2.15878800
N	3.51008500	0.82419000	0.35623000
C	3.81184100	2.27142500	0.11667000
H	3.63043900	2.78151500	1.06457000
H	4.87638400	2.37222700	-0.12033300
C	3.50445800	-0.01384800	-0.88613000
H	3.28973700	-1.03196900	-0.55659100
H	2.65057600	0.31862400	-1.47868500
C	4.38278700	0.22327700	1.41845900
H	4.05074500	-0.81461400	1.51917700
H	5.41754400	0.21697600	1.06336400
C	4.79139500	0.01345200	-1.70345800
H	5.65520300	-0.33265100	-1.12687000
H	4.67069400	-0.65991500	-2.55957700
H	5.01234700	1.01195000	-2.09335700
C	2.93825500	2.89721200	-0.96581400
H	3.08903300	3.98218600	-0.95183700
H	3.19226900	2.53628900	-1.96701200
H	1.87955000	2.69020300	-0.78696900
C	4.28029700	0.93362100	2.76576200
H	4.76370000	0.31164400	3.52678400
H	4.78233800	1.90556700	2.76332800
H	3.23537700	1.08304400	3.05697200
H	2.42651900	0.78201700	0.78718000

^{t}17

E (SMD/B3LYP-D3/BS1) = -1279.65924913 au
G (SMD/B3LYP-D3/BS1) = -1279.281214 au
E (SMD/B3LYP-D3/BS2//SMD/B3LYP-D3/BS1) = -2617.50026489 au
charge = 2 spin multiplicity = 3

Ni	-0.07102900	-0.33188600	-0.45187800
N	0.11024400	-1.29276600	1.45316300
C	1.34999000	-2.14738100	1.54772500
H	2.09674500	-1.60599800	2.12895200
C	0.13379500	-0.30533600	2.57083300
H	0.38535300	-0.84162900	3.49366900
H	-0.86223800	0.11683200	2.69246700
C	-2.44950500	-1.50546100	1.52513400
C	2.61056200	-1.39340600	-0.49766500
C	3.96754900	-1.38312600	-0.82634500
C	4.51976500	-0.28955500	-1.48744700
H	4.57408300	-2.24230100	-0.56146500
C	2.36056500	0.73708900	-1.46163900
C	3.69880000	0.78876300	-1.81871900
H	5.57418600	-0.28087700	-1.74530700
H	1.68272100	1.54633500	-1.69809700
H	4.07775800	1.65910300	-2.34248500
C	0.56792800	1.89873200	1.42614400
C	0.65410500	3.24990500	1.77377700
C	-0.51728400	2.47901800	-0.56065600
C	0.14367100	4.22708500	0.92655400
H	1.12579800	3.51664800	2.71342200
C	-0.45530700	3.83218500	-0.26947800
H	-0.96057800	2.13657300	-1.48579600
H	0.21074600	5.27695600	1.19450600
H	-0.86848700	4.54891000	-0.97017200
C	-2.90284400	-0.86433500	0.24209800
C	-4.27211500	-0.80408600	-0.04601200
C	-4.72108700	-0.24290200	-1.23434800
H	-4.97054000	-1.21580900	0.67487600
C	-2.44275700	0.15298700	-1.82245700
C	-3.78325300	0.23524500	-2.15167800
H	-5.78331400	-0.19950200	-1.45376300
H	-1.67443200	0.47613700	-2.51474900
H	-4.07415500	0.65190100	-3.10919500
N	-2.01777800	-0.34836100	-0.63701400
N	1.84158400	-0.32031900	-0.80178500
N	-0.02319500	1.52946100	0.26558900
C	-1.08482100	-2.18898300	1.54090200
H	-1.01344900	-2.74850200	2.48191100
H	-1.00691400	-2.89959900	0.71460600

H	-2.53880600	-0.77110900	2.33308100
H	-3.17830400	-2.28473800	1.76904100
C	1.12165000	0.83736800	2.33298400
H	2.06764300	0.46680700	1.92029400
H	1.36080500	1.29340300	3.29667200
H	1.09079100	-3.04697400	2.11361300
C	1.97329200	-2.55494100	0.21394800
H	1.22404000	-3.02581100	-0.42857700
H	2.74321300	-3.30166600	0.42477000
O	-0.18825700	-1.68269500	-1.56141200

^{s}17

E (SMD/B3LYP-D3/BS1) = -1279.62672080 au

G (SMD/B3LYP-D3/BS1) = -1279.246330 au

E (SMD/B3LYP-D3/BS2//SMD/B3LYP-D3/BS1) = -2617.47069658 au

charge = 2 spin multiplicity = 1

Ni	-0.04516900	-0.40640600	-0.44325400
N	0.10073300	-1.25000800	1.43102100
C	1.33561300	-2.12653500	1.50140700
H	2.09720400	-1.58528000	2.06200200
C	0.14362700	-0.26240700	2.55144800
H	0.41282700	-0.81876300	3.45614900
H	-0.85315200	0.14930400	2.69208600
C	-2.46245600	-1.46580800	1.57369500
C	2.61931700	-1.40283400	-0.49682200
C	3.97132400	-1.42110100	-0.83608400
C	4.55671300	-0.29175500	-1.40434700
H	4.54596900	-2.32223900	-0.65192300
C	2.43550000	0.80935100	-1.25627800
C	3.77463200	0.84286000	-1.61995600
H	5.60745800	-0.29797200	-1.67677700
H	1.78594300	1.66006900	-1.41264800
H	4.18289000	1.74450400	-2.06245200
C	0.54081500	1.91108300	1.38605400
C	0.63640100	3.27563500	1.67064800
C	-0.60652500	2.39254000	-0.59056300
C	0.09704200	4.21032500	0.79389000
H	1.13858800	3.58409700	2.58123900
C	-0.53795400	3.75826300	-0.36295100
H	-1.07101400	2.00797400	-1.48804200
H	0.17043200	5.27191000	1.00874900
H	-0.97340700	4.44130300	-1.08350700
C	-2.92133600	-0.85224500	0.28252900
C	-4.29476600	-0.75301300	0.02206500
C	-4.74451300	-0.23916600	-1.18577900
H	-4.99104600	-1.10034800	0.77821600
C	-2.45911800	0.01702400	-1.83452000

C	-3.80170200	0.14219400	-2.14288300
H	-5.80783600	-0.15912600	-1.38937600
H	-1.69413000	0.27095300	-2.55610500
H	-4.09188700	0.52112800	-3.11641900
N	-2.02697800	-0.43341100	-0.63258900
N	1.88823500	-0.28453600	-0.69484400
N	-0.08725200	1.48673200	0.26693100
C	-1.10274900	-2.14560400	1.55165100
H	-0.98881000	-2.68592300	2.49813600
H	-1.04045700	-2.86803700	0.73451900
H	-2.53632000	-0.72649400	2.37777300
H	-3.18109700	-2.24800900	1.83906400
C	1.11891200	0.88512600	2.31641200
H	2.08006800	0.53034000	1.92671200
H	1.32808100	1.35747200	3.27919400
H	1.05879100	-3.01179600	2.07794800
C	1.92305300	-2.55915100	0.15791800
H	1.13907500	-2.96494300	-0.49155400
H	2.64259900	-3.35909300	0.34908300
O	-0.01877300	-1.37397900	-1.79747400

$^{t}TS_{17\text{-}18}$

E (SMD/B3LYP-D3/BS1) = -1511.91979193 au
G (SMD/B3LYP-D3/BS1) = -1511.450402 au
E (SMD/B3LYP-D3/BS2//SMD/B3LYP-D3/BS1) = -2849.84705248 au
negative eigenvalue of the frequency: 337i
charge = 2 spin multiplicity = 3

Ni	0.22736300	0.03959000	-0.17991400
N	0.26245300	-0.13762600	1.94904000
C	-0.08087900	-1.53500400	2.39794400
H	0.84322900	-2.04358200	2.67491300
C	1.57887800	0.23638700	2.54526600
H	1.56224500	-0.02554300	3.61054600
H	1.70304200	1.31586700	2.47231000
C	-0.64836300	2.26240900	2.14400000
C	0.10039800	-2.81130800	0.24452700
C	0.35513600	-4.15264700	-0.04656200
C	1.22880600	-4.48807200	-1.07821300
H	-0.13910000	-4.91995600	0.53951400
C	1.56319200	-2.15327500	-1.47298300
C	1.84489900	-3.46985300	-1.80738700
H	1.42528200	-5.53017800	-1.31111900
H	2.00687600	-1.32226700	-2.00701600
H	2.52935100	-3.68195200	-2.62114400
C	3.17893600	0.27718100	0.58978600
C	4.52194500	0.56844500	0.33206200
C	2.56435500	1.27839500	-1.42237800

C	4.88062000	1.22656800	-0.84017500
H	5.27202500	0.27486800	1.05875300
C	3.87833300	1.59197600	-1.73891600
H	1.75962400	1.53396400	-2.09968200
H	5.92212500	1.45368800	-1.04677800
H	4.10030800	2.10750900	-2.66685800
C	-0.75509600	2.72470200	0.71475700
C	-1.20393000	4.02742000	0.45597400
C	-1.34234500	4.47801000	-0.85004600
H	-1.45092200	4.66830500	1.29593200
C	-0.60814800	2.32865100	-1.58449800
C	-1.04443100	3.60394800	-1.89794400
H	-1.69254400	5.48605000	-1.04976600
H	-0.39752400	1.60240900	-2.35990000
H	-1.15649700	3.89281200	-2.93696600
N	-0.44407500	1.90461800	-0.30881600
N	0.72387900	-1.84243500	-0.46644200
N	2.21494900	0.63639600	-0.28790300
C	-0.81949300	0.77324400	2.43438900
H	-0.89343200	0.66342500	3.52403300
H	-1.75209000	0.41129100	1.99682800
H	0.28634400	2.64618000	2.56775100
H	-1.44066200	2.76918200	2.70496800
C	2.77047700	-0.43067800	1.85268700
H	2.56062300	-1.48317800	1.62639900
H	3.61717500	-0.42734600	2.54443100
H	-0.69020600	-1.46146600	3.30437500
C	-0.81451000	-2.38747700	1.35976700
H	-1.67064700	-1.84263500	0.95221300
H	-1.19398800	-3.27918000	1.86640800
O	-1.25862600	-0.56035800	-0.96876800
C	-3.63547800	-1.10198100	-1.82905000
C	-3.18383500	0.17925300	-1.41584400
C	-3.50364400	0.64269600	-0.11541900
C	-4.16868900	-0.19163100	0.77723000
C	-4.55691500	-1.47590900	0.37377200
C	-4.29636800	-1.92681300	-0.93232500
H	-3.42721100	-1.43659300	-2.84064100
H	-2.79811900	0.87277600	-2.15248500
H	-3.21514100	1.64550000	0.17877300
H	-4.39448900	0.15374200	1.78177500
H	-5.07478000	-2.12647500	1.07314400
H	-4.62159200	-2.91739300	-1.23664400

^{S}TS$_{17}$
E (SMD/B3LYP-D3/BS1) = -1511.89485386 au
G (SMD/B3LYP-D3/BS1) = -1511.421041 au

E (SMD/B3LYP-D3/BS2//SMD/B3LYP-D3/BS1) = -2849.82510305 au
negative eigenvalue of the frequency: 168i
charge = 2 spin multiplicity = 1

Ni	-0.20393500	-0.04989800	-0.27531100
N	-0.20457200	0.18541200	1.95955600
C	0.32340800	1.54923300	2.30104000
H	-0.50448300	2.16276700	2.66003600
C	-1.53131000	0.01141600	2.60724000
H	-1.48595800	0.42624900	3.62187200
H	-1.75214100	-1.05066600	2.70321900
C	0.42474900	-2.28250300	2.11777700
C	0.00974400	2.74950600	0.12810200
C	-0.23691000	4.09963200	-0.12703600
C	-1.20396800	4.46302800	-1.06066300
H	0.33555500	4.84870800	0.40927300
C	-1.63144500	2.13777700	-1.43215600
C	-1.91401000	3.46333400	-1.72769900
H	-1.39875800	5.51082300	-1.26788000
H	-2.14479700	1.32290500	-1.92632600
H	-2.67170100	3.69652900	-2.46735900
C	-3.07263700	-0.15543400	0.63070100
C	-4.41928900	-0.47843100	0.43391800
C	-2.50484100	-1.37058000	-1.28388600
C	-4.80578900	-1.25812500	-0.65031800
H	-5.15144000	-0.10768200	1.14307700
C	-3.82470600	-1.71667300	-1.52856500
H	-1.72163200	-1.69798400	-1.95299000
H	-5.85157300	-1.50493800	-0.80568300
H	-4.06515400	-2.32778300	-2.39139900
C	0.67346500	-2.74510800	0.70895100
C	1.17224800	-4.03883200	0.50221400
C	1.39270300	-4.51465900	-0.78328200
H	1.38617700	-4.65635300	1.36798500
C	0.64611900	-2.39795600	-1.59832500
C	1.11829100	-3.67227700	-1.86126500
H	1.77792800	-5.51730600	-0.94176500
H	0.46131500	-1.69556100	-2.40006200
H	1.27462600	-3.98283900	-2.88833200
N	0.41621800	-1.94045500	-0.34505000
N	-0.70328900	1.80047000	-0.51676400
N	-2.13093000	-0.61022900	-0.23094600
C	0.76959100	-0.83103300	2.44106700
H	0.85347700	-0.73427500	3.53252100
H	1.73808600	-0.57411200	2.00723100
H	-0.61521800	-2.51104800	2.37608600
H	1.03153400	-2.90553100	2.78117200
C	-2.65754600	0.67342300	1.81422400

H	-2.36740300	1.67784700	1.48662200
H	-3.52227500	0.79432700	2.47134700
H	1.03407800	1.44567500	3.12772500
C	1.00813000	2.27938300	1.14159400
H	1.73976200	1.62270500	0.66800000
H	1.54973500	3.13707700	1.54851800
O	0.98828700	0.29035100	-1.44895800
C	3.42137800	1.31402800	-1.65965000
C	3.10899300	-0.05247700	-1.42575300
C	3.45877400	-0.64207600	-0.18281300
C	4.08296600	0.11852900	0.79530700
C	4.38569300	1.47018400	0.54859600
C	4.04810600	2.06656200	-0.67484900
H	3.16759300	1.75657000	-2.61727700
H	2.85052700	-0.69448500	-2.25656300
H	3.24934300	-1.69358000	-0.01611000
H	4.35192700	-0.32967900	1.74724000
H	4.88101100	2.05774600	1.31638500
H	4.28033400	3.11262800	-0.85058000

^{t}18

E (SMD/B3LYP-D3/BS1) = -1511.9920421 au

G (SMD/B3LYP-D3/BS1) = -1511.519590 au

E (SMD/B3LYP-D3/BS2//SMD/B3LYP-D3/BS1) = -2849.91137118 au

charge = 2 spin multiplicity = 3

Ni	-0.04925400	-0.02945300	0.06294500
N	0.04185400	-0.30043800	2.11468400
C	-1.30467600	-0.35830000	2.76610800
H	-1.71653300	-1.35628100	2.62197300
C	0.81266300	-1.54042800	2.42066900
H	0.84444200	-1.67862500	3.50944800
H	1.83443200	-1.39683600	2.06981100
C	2.23842800	1.03359700	2.40146000
C	-2.95677900	0.21549900	0.94137100
C	-4.34454900	0.21189400	0.77828800
C	-4.89883800	-0.23053500	-0.42029100
H	-4.97539800	0.55372800	1.59210500
C	-2.67982400	-0.64827800	-1.20345200
C	-4.04863500	-0.67811600	-1.43243600
H	-5.97574900	-0.23362400	-0.55966300
H	-1.98209700	-0.97563900	-1.96380700
H	-4.43044600	-1.04122700	-2.38034200
C	0.50461500	-3.00105600	0.28993000
C	0.87287700	-4.26245300	-0.19353700
C	0.43436000	-2.18433700	-1.89400800
C	1.01450900	-4.47530300	-1.56122200
H	1.03894000	-5.06953500	0.51240000

C	0.78039100	-3.41362100	-2.43533300
H	0.24642900	-1.32771000	-2.53207800
H	1.29926500	-5.45304800	-1.93816100
H	0.86764700	-3.52599400	-3.51041900
C	2.79234100	0.92999400	1.00125800
C	4.12946200	1.30047200	0.79680200
C	4.70640200	1.17187700	-0.45886600
H	4.69999500	1.68502300	1.63634800
C	2.61287000	0.32270500	-1.23217400
C	3.92816000	0.66220700	-1.50109800
H	5.74024300	1.45998600	-0.62404800
H	1.97791500	-0.05708600	-2.01998100
H	4.32428400	0.53451900	-2.50262300
N	2.03985600	0.45613500	-0.01369200
N	-2.14080000	-0.20261900	-0.05190700
N	0.30583500	-1.97276100	-0.56639700
C	0.73297100	0.93866600	2.61432300
H	0.55352000	1.02543300	3.69320800
H	0.24510000	1.78971400	2.12902300
H	2.75899900	0.29550400	3.02388100
H	2.53585900	2.00755100	2.80792500
C	0.26117300	-2.81544800	1.76989700
H	-0.81659300	-2.91403900	1.94555800
H	0.72231700	-3.65853400	2.29213400
H	-1.16609500	-0.21197900	3.84424400
C	-2.31289800	0.66486400	2.22556400
H	-1.83725500	1.64034800	2.07339900
H	-3.08929900	0.81024200	2.98133400
O	-0.21283500	1.00257400	-1.83301300
C	-1.15320800	2.06330700	-2.23261900
C	0.32936100	2.31145900	-2.23795900
C	0.88631900	3.15829800	-1.17245100
C	0.10072200	3.57787000	-0.15337000
C	-1.33526600	3.34727900	-0.15368100
C	-1.94805100	2.69152900	-1.16673100
H	-1.65282500	1.80731700	-3.16226500
H	0.87885700	2.23152900	-3.17114400
H	1.93067400	3.44461600	-1.24291900
H	0.52311600	4.17845400	0.64735100
H	-1.92524200	3.78732900	0.64516200
H	-3.02933400	2.62887700	-1.23367100

^{t}TS$_{18\text{-}19}$

E (SMD/B3LYP-D3/BS1) = -1511.96783935 au

G (SMD/B3LYP-D3/BS1) = -1511.497004 au

E (SMD/B3LYP-D3/BS2//SMD/B3LYP-D3/BS1) = -2849.88673099 au

negative eigenvalue of the frequency: -111i

charge = 2 spin multiplicity = 3

Ni	-0.05429800	-0.03458200	0.00360500
N	-0.35980500	0.11549100	2.09858300
C	0.89150600	0.44273400	2.85362700
H	1.06034000	1.51794500	2.80089500
C	-1.39542400	1.14868300	2.38800200
H	-1.50208600	1.25373500	3.47631100
H	-2.34770800	0.79732800	1.99118600
C	-2.23364700	-1.65980300	2.25427600
C	2.74137400	0.43881200	1.14500800
C	4.09514600	0.78408600	1.08539700
C	4.57666900	1.49026500	-0.01495000
H	4.75245200	0.50491700	1.90235500
C	2.36027700	1.45446300	-0.91470700
C	3.68930000	1.84491400	-1.03222600
H	5.62563300	1.76523700	-0.07434900
H	1.63363900	1.67586500	-1.68618600
H	4.01518800	2.40225500	-1.90388800
C	-1.42774300	2.67309600	0.30314500
C	-2.14440800	3.78646400	-0.14983300
C	-1.19936400	1.91436200	-1.89001500
C	-2.38192200	3.95621100	-1.51106200
H	-2.50576000	4.51228700	0.57126700
C	-1.89093300	3.00344900	-2.40372100
H	-0.78998300	1.14333800	-2.53247300
H	-2.93705900	4.81883400	-1.86783500
H	-2.03983100	3.09359800	-3.47439900
C	-2.71764700	-1.67788800	0.82533600
C	-3.98477700	-2.22216500	0.56677200
C	-4.46918400	-2.27061500	-0.73312400
H	-4.57123400	-2.60421700	1.39688000
C	-2.42115100	-1.25694900	-1.43692900
C	-3.66263200	-1.78005400	-1.76320900
H	-5.44981200	-2.68814200	-0.94229300
H	-1.74344800	-0.88533500	-2.19402400
H	-3.98414100	-1.80207900	-2.79914200
N	-1.94984000	-1.18985500	-0.16924500
N	1.89872400	0.76294400	0.14279900
N	-0.97770600	1.74421000	-0.57002100
C	-0.79162300	-1.25712500	2.53190400
H	-0.64288700	-1.34411700	3.61603700
H	-0.11477100	-1.96972900	2.05093600
H	-2.92618800	-1.05543000	2.85137900
H	-2.33759800	-2.67823300	2.64978100
C	-1.10343000	2.52425900	1.76979500
H	-0.05317000	2.80330400	1.91335900
H	-1.68964700	3.26180700	2.32527000

H	0.73002000	0.19169100	3.90919600
C	2.14690800	-0.27057400	2.33374200
H	1.92051700	-1.31016100	2.07031800
H	2.88721700	-0.30455500	3.13785000
O	0.38345600	-0.62648900	-1.87735300
C	2.39869500	-1.44698700	-2.35346400
C	0.99686900	-1.88851400	-2.12308800
C	0.83162100	-2.81576800	-0.95583000
C	1.86425800	-3.10591000	-0.11792600
C	3.15729100	-2.56134800	-0.38219100
C	3.42711800	-1.74052800	-1.48213900
H	2.55078700	-0.78148200	-3.19698300
H	-0.16475400	-3.20695300	-0.77754000
H	1.72529600	-3.73509700	0.75481500
H	3.96015600	-2.78082900	0.31674300

^{t}19

E (SMD/B3LYP-D3/BS1) = -1512.04167438 au
G (SMD/B3LYP-D3/BS1) = -1511.573572 au
E (SMD/B3LYP-D3/BS2//SMD/B3LYP-D3/BS1) = -2849.96391708 au
charge = 2 spin multiplicity = 3

Ni	-0.30402100	0.04177500	-0.11719900
N	-1.22065600	-0.28209800	-1.98178200
C	-0.52096900	-1.32835300	-2.81199600
H	-1.02601300	-2.28267200	-2.66189900
C	-2.64330200	-0.70096200	-1.78801500
H	-3.06133100	-0.96738600	-2.76726300
H	-3.20506100	0.15162100	-1.40803800
C	-1.88311300	2.22168400	-2.14168200
C	1.22057100	-2.28031900	-1.23697200
C	1.98505400	-3.44953000	-1.21477300
C	2.16464600	-4.13625200	-0.01543700
H	2.42900400	-3.81152800	-2.13624200
C	0.81355500	-2.47962900	1.05274200
C	1.56362900	-3.64522200	1.14416800
H	2.76002200	-5.04407300	0.01179000
H	0.32715400	-2.05063500	1.92184300
H	1.67120800	-4.14749200	2.09941000
C	-2.84226300	-1.47102900	0.64408600
C	-3.81968500	-1.95803900	1.51751700
C	-1.84938600	-0.28796800	2.38979000
C	-3.79163400	-1.59621100	2.86178100
H	-4.59189900	-2.61674900	1.13402100
C	-2.78312800	-0.74262100	3.31111300
H	-1.04405400	0.37339300	2.68900700
H	-4.54535000	-1.97238000	3.54714200
H	-2.71670000	-0.43225300	4.34818000

C	-1.37242600	2.84115600	-0.86413000
C	-1.53287100	4.21836700	-0.65754500
C	-1.08506300	4.80413000	0.52074300
H	-2.00527600	4.81605000	-1.43046800
C	-0.32990100	2.64705900	1.20793300
C	-0.46130000	4.00025200	1.47761400
H	-1.20834800	5.87030600	0.68642300
H	0.16981200	1.98552900	1.90697000
H	-0.07877700	4.40815500	2.40689300
N	-0.78750400	2.07169900	0.07490400
N	0.65072700	-1.81063400	-0.10373100
N	-1.87426000	-0.64068400	1.08891200
C	-1.13683600	1.01812700	-2.72607300
H	-1.53730200	0.85567800	-3.73515800
H	-0.07326200	1.25346200	-2.82156600
H	-2.94861300	1.99643100	-2.01686300
H	-1.84749100	2.99340100	-2.91818200
C	-2.82716000	-1.87098500	-0.80898400
H	-2.04690300	-2.62837100	-0.95163500
H	-3.77634400	-2.36042000	-1.04405600
H	-0.64993400	-1.06383500	-3.86721100
C	0.97139300	-1.51637600	-2.51321300
H	1.47463400	-0.54563400	-2.47090400
H	1.40896200	-2.07209300	-3.34747000
O	1.61538100	0.86182400	-0.71497700
C	2.65744700	0.46249200	1.37634300
C	2.64978500	0.87157500	-0.00485900
C	3.94512900	1.33148500	-0.62636800
C	5.15034900	1.31473300	0.25315500
C	5.08254300	0.90809800	1.53796700
C	3.81701000	0.48250700	2.09523300
H	1.71981200	0.14358900	1.82136900
H	4.12537500	0.71180100	-1.51885500
H	3.78958400	2.34420300	-1.02962800
H	6.08778600	1.64580500	-0.18526200
H	5.96191500	0.89675700	2.17410500
H	3.79633100	0.16806000	3.13598100

^{t}TS$_{19\text{-}8}$

E (SMD/B3LYP-D3/BS1) = -1741.33343821 au
G (SMD/B3LYP-D3/BS1) = -1740.798022 au
E (SMD/B3LYP-D3/BS2//SMD/B3LYP-D3/BS1) = -3079.39924736 au
negative eigenvalue of the frequency: 1171i
charge = 2 spin multiplicity = 3

O	-1.42950600	0.24471000	-0.58033500
C	-3.16708900	1.83944500	-0.78112600
C	-2.15163900	1.17365800	-0.03696000

C	-2.04030600	1.49246200	1.37039900
C	-2.72726100	2.64601500	1.88718700
C	-3.66465600	3.29980900	1.12451200
C	-3.88359400	2.87374300	-0.21155500
H	-3.32246300	1.55057300	-1.81642000
H	-2.92473800	0.53794300	1.77686900
H	-1.13912100	1.15045500	1.88193400
H	-2.54084600	2.94162400	2.91606700
H	-4.22911200	4.13533800	1.52582600
H	-4.63031200	3.38802100	-0.81193300
O	-3.76329200	-0.37399800	2.17112300
H	-3.31147600	-0.92772800	2.83827100
H	-4.00230000	-0.99646500	1.38493500
O	-4.40672600	-1.95547000	0.23394200
H	-4.13167600	-2.85766100	0.47435900
H	-3.91616500	-1.76402600	-0.61680800
O	-2.96348300	-1.45948100	-1.98205400
H	-2.45373600	-2.27550200	-2.13048700
H	-2.31709300	-0.82840800	-1.57223500
Ni	0.58790500	-0.17397300	-0.09267600
N	1.54859400	-0.53333500	-1.95029400
C	1.54244500	0.67971000	-2.84744300
H	2.49580300	1.19652500	-2.73344000
C	2.96675700	-0.95230100	-1.72517000
H	3.46819000	-1.00767900	-2.69993500
H	2.96535500	-1.95381200	-1.29625200
C	0.69872500	-2.98472900	-1.95414300
C	0.65952200	2.53240900	-1.36767900
C	0.66629000	3.92803200	-1.42677600
C	0.92988300	4.66751600	-0.27498400
H	0.46741700	4.42135600	-2.37251800
C	1.17981300	2.60548000	0.90642900
C	1.19902500	3.99462000	0.91737200
H	0.93158300	5.75300500	-0.30973400
H	1.37560400	2.02878500	1.80396600
H	1.41575700	4.52740600	1.83698600
C	3.55105900	-0.30752500	0.67917400
C	4.63546800	-0.39756100	1.55766400
C	2.07224000	-0.67983800	2.44075000
C	4.41352100	-0.62806100	2.91279900
H	5.64253000	-0.28451600	1.16987400
C	3.10200800	-0.77142900	3.36758000
H	1.03675100	-0.77721200	2.74601000
H	5.24976600	-0.69643800	3.60230200
H	2.87589800	-0.95020400	4.41320300
C	-0.16779300	-3.11111700	-0.72499800
C	-0.92795900	-4.27164700	-0.52225800

C	-1.69561800	-4.40883100	0.63055500
H	-0.90581400	-5.05644000	-1.27138300
C	-0.95029400	-2.23748100	1.28633000
C	-1.70584500	-3.36786700	1.56228700
H	-2.28122000	-5.30798900	0.79701700
H	-0.94805700	-1.38404700	1.95659000
H	-2.29214800	-3.42157600	2.47369800
N	-0.18854000	-2.11318700	0.18133400
N	0.90827500	1.89346200	-0.20193100
N	2.28594500	-0.45423500	1.12898100
C	0.78593100	-1.61876700	-2.64856600
H	1.25555800	-1.78660600	-3.62696700
H	-0.22084000	-1.23122500	-2.82836500
H	1.69932200	-3.36072800	-1.71106600
H	0.30807900	-3.67633300	-2.70742800
C	3.75750400	-0.02797400	-0.78727300
H	3.51356500	1.02350000	-0.97940800
H	4.82016900	-0.14399700	-1.01703400
H	1.49141900	0.33288900	-3.88505000
C	0.41014200	1.68088000	-2.58733800
H	-0.54301800	1.15403700	-2.48207900
H	0.33050800	2.33143800	-3.46306200

^{qnt}20

E (SMD/B3LYP-D3/BS1) = -2560.24771003 au
G (SMD/B3LYP-D3/BS1) = -2559.459710 au
E (SMD/B3LYP-D3/BS2//SMD/B3LYP-D3/BS1) = -5235.91778564 au
charge = 3 spin multiplicity = 5

Ni	2.11555800	0.21801500	0.37856900
N	3.70806400	1.16259600	1.32324400
C	4.34100400	0.29942300	2.37197600
H	5.11039000	-0.31245700	1.90089800
C	4.73587100	1.58736300	0.32889600
H	5.58128500	2.03955900	0.86535900
H	4.29525400	2.35617500	-0.30589600
C	2.64732900	3.51440600	1.14170500
C	3.03905700	-1.87452100	2.32704600
C	3.22677700	-3.15120000	2.86346100
C	2.93393900	-4.27553900	2.09302900
H	3.60314000	-3.25268900	3.87624900
C	2.30838400	-2.80045900	0.31917200
C	2.46488100	-4.09881100	0.79163600
H	3.07415200	-5.27225500	2.50103900
H	1.94330700	-2.61205400	-0.68347800
H	2.22060900	-4.94000500	0.15250300
C	4.35546700	0.05179600	-1.71776500
C	4.88311800	-0.15278700	-2.99895100

C	2.26204300	-0.63905800	-2.46096700
C	4.06474100	-0.61828000	-4.02425700
H	5.93443700	0.04907600	-3.17666300
C	2.72206300	-0.88001100	-3.74763200
H	1.22857500	-0.83282600	-2.19530600
H	4.46874800	-0.77928500	-5.01940300
H	2.04308300	-1.25909500	-4.50389600
C	1.63769800	3.28597700	0.04180700
C	1.02301300	4.40843100	-0.53478600
C	0.09718500	4.25050800	-1.55760300
H	1.27932600	5.39587700	-0.16377000
C	0.42515600	1.89556600	-1.36200900
C	-0.21278500	2.95667600	-1.98330700
H	-0.38318700	5.11470700	-2.00634400
H	0.19012400	0.87466600	-1.63329200
H	-0.94063600	2.76855700	-2.76499900
N	1.34078700	2.04300700	-0.38182800
N	2.58333000	-1.71489300	1.06403200
N	3.04801500	-0.17320600	-1.46829100
C	3.09439700	2.34315600	2.01457400
H	3.82536700	2.74696200	2.72763700
H	2.24357300	1.96771200	2.58745800
H	3.51808500	4.01868600	0.70601500
H	2.21628500	4.24831200	1.83370900
C	5.25759300	0.46596400	-0.58040300
H	5.49703400	-0.43259100	0.00116900
H	6.20584400	0.80972600	-1.00386200
H	4.84699800	0.95212100	3.09377100
C	3.36577400	-0.62962100	3.11089300
H	2.43781300	-0.10096100	3.35317900
H	3.82852500	-0.91926200	4.05867500
O	0.67011700	0.22932500	1.79467600
O	-0.14439400	-0.42033300	0.73541700
H	-0.08230600	-1.35764900	0.99903800
Ni	-2.15953900	-0.08228200	-0.17877600
N	-3.72524700	0.01093700	-1.61399000
C	-3.35428900	-0.73421400	-2.87001100
H	-3.75837900	-1.74564700	-2.80530000
C	-4.98655500	-0.57131200	-1.05717700
H	-5.68450100	-0.73934600	-1.88677100
H	-5.44138900	0.16060600	-0.39137500
C	-4.33048600	2.40864600	-0.90889800
C	-1.28220100	-1.96626800	-2.30831500
C	-0.65945300	-3.07208400	-2.89053600
C	-0.33883100	-4.17415000	-2.09971500
H	-0.45281200	-3.06629100	-3.95551600
C	-1.26817600	-3.01259100	-0.22649400

C	-0.67242800	-4.15523500	-0.74440300
H	0.14547400	-5.04156100	-2.53768300
H	-1.54712300	-2.95134500	0.82112800
H	-0.46618400	-4.99799100	-0.09479400
C	-4.29792100	-1.68889600	1.13996300
C	-4.95457900	-2.29059800	2.21757100
C	-2.71768600	-0.77303100	2.60103500
C	-4.46560600	-2.12440700	3.51105000
H	-5.84224100	-2.88570200	2.03066000
C	-3.32104500	-1.35076200	3.70929100
H	-1.81899700	-0.17369400	2.69578400
H	-4.97002200	-2.59186700	4.35137400
H	-2.89993200	-1.19595700	4.69670000
C	-3.26664200	2.80231400	0.07949300
C	-3.30256400	4.09797200	0.60972100
C	-2.36692900	4.49726300	1.55609500
H	-4.07338900	4.78131700	0.26831100
C	-1.40013000	2.31537300	1.37635100
C	-1.39267600	3.58073900	1.94615700
H	-2.39222900	5.50121100	1.96942900
H	-0.62984400	1.59820300	1.63991400
H	-0.62557100	3.83300000	2.67060000
N	-2.31785100	1.91576600	0.46296700
N	-1.54373600	-1.93167200	-0.98283600
N	-3.19430800	-0.93493700	1.34971500
C	-3.94317300	1.43586300	-2.01595000
H	-4.74106400	1.45568900	-2.77014800
H	-3.02119600	1.77316400	-2.49754900
H	-5.20288200	2.04601500	-0.35299400
H	-4.67209600	3.31986400	-1.41055900
C	-4.78867600	-1.87702700	-0.27502100
H	-4.10179300	-2.54392900	-0.80831100
H	-5.75307400	-2.39054600	-0.23672700
H	-3.84612100	-0.24554000	-3.71733900
C	-1.83818700	-0.82292500	-3.11329400
H	-1.35472900	0.12546300	-2.84893300
H	-1.65026000	-0.99367900	-4.17628000

^{t}2

E (SMD/B3LYP-D3/BS1) = -2559.76084642 au
G (SMD/B3LYP-D3/BS1) = -2558.990650 au
E (SMD/B3LYP-D3/BS2//SMD/B3LYP-D3/BS1) = -5235.43335161 au
charge = 2 spin multiplicity = 3

Ni	1.82215500	-0.00154300	0.25192400
N	3.09949900	0.25202500	2.04218300
C	3.12226100	-0.97594100	2.90090000
H	3.98894700	-1.57825500	2.62477900

C	4.48547400	0.60882500	1.63250000
H	5.13000900	0.64275900	2.52252600
H	4.46813100	1.60896600	1.19978200
C	2.62724900	2.78400900	2.34765400
C	1.93633500	-2.77537800	1.60131800
C	1.97339400	-4.16764300	1.72462500
C	2.09600900	-4.95324600	0.57942600
H	1.90735700	-4.62033000	2.70885600
C	2.13266200	-2.93856000	-0.70987100
C	2.17661500	-4.32896100	-0.66621300
H	2.12324800	-6.03628700	0.65775500
H	2.18613100	-2.39670700	-1.64830100
H	2.26338200	-4.90030400	-1.58430900
C	4.73815700	-0.06665600	-0.84629600
C	5.72944500	0.01951800	-1.83119700
C	3.08661000	0.23037500	-2.46211400
C	5.37004000	0.21193700	-3.16317500
H	6.77255100	-0.07052700	-1.54554000
C	4.01718500	0.31185900	-3.49081000
H	2.02107300	0.29367200	-2.65574900
H	6.13340600	0.27818800	-3.93308900
H	3.68608500	0.44987000	-4.51466500
C	2.11672600	3.18943600	0.98432400
C	2.10883400	4.55460300	0.65592100
C	1.59273300	4.96898200	-0.56524000
H	2.49610000	5.27371100	1.37166600
C	1.11860900	2.67549600	-1.04418500
C	1.07283600	4.00601000	-1.43502100
H	1.57771000	6.02272700	-0.82859200
H	0.69310300	1.88429400	-1.64833500
H	0.63244000	4.27805700	-2.38853600
N	1.64749300	2.26719300	0.12809500
N	2.02180900	-2.18416900	0.39334600
N	3.43412100	0.05516800	-1.17319700
C	2.44089900	1.34519500	2.82731400
H	2.82620800	1.31979100	3.85617200
H	1.37497200	1.10170100	2.87005000
H	3.67771000	3.08417000	2.43668400
H	2.09877100	3.40307500	3.08460500
C	5.10070000	-0.34654600	0.59332400
H	4.82645200	-1.38450800	0.81635600
H	6.18873000	-0.28904600	0.69028100
H	3.27180800	-0.67037800	3.94391000
C	1.86351200	-1.85696400	2.79332400
H	0.96728700	-1.23075900	2.71414700
H	1.77628500	-2.45198300	3.70724500
O	-0.20458800	-0.24566200	0.69348600

Ni	-1.91234800	-0.11656500	-0.31094800
N	-3.17818600	0.04246800	-2.00868100
C	-3.14544700	-1.18625600	-2.86539600
H	-3.92211400	-1.87018300	-2.52151300
C	-4.58261000	0.31992300	-1.59559600
H	-5.22735900	0.31793400	-2.48614400
H	-4.62120800	1.31942100	-1.16283900
C	-2.86740500	2.58750800	-2.32521500
C	-1.67525400	-2.82239500	-1.64663400
C	-1.40320400	-4.18997800	-1.74455300
C	-1.32425800	-4.95679300	-0.58321500
H	-1.25429600	-4.63806600	-2.72189200
C	-1.78998600	-2.97183500	0.66763900
C	-1.52350700	-4.33736600	0.65132400
H	-1.10676000	-6.01942000	-0.64047200
H	-1.93821200	-2.43635400	1.59826400
H	-1.46478000	-4.89178300	1.58185100
C	-4.74312600	-0.40579100	0.87394600
C	-5.69822200	-0.41361600	1.89806700
C	-3.05000400	-0.06843400	2.43162700
C	-5.29759600	-0.24675900	3.22137200
H	-6.74475600	-0.55657400	1.64848400
C	-3.94010200	-0.07943600	3.49856400
H	-1.98324600	0.04631900	2.58829900
H	-6.03122300	-0.25299500	4.02243300
H	-3.57471200	0.03993100	4.51314400
C	-2.38991400	2.98136500	-0.94806200
C	-2.46708500	4.33431100	-0.58278200
C	-1.98245100	4.74717500	0.65123900
H	-2.89418300	5.04649400	-1.28261900
C	-1.36844200	2.47384700	1.06612400
C	-1.40600700	3.79299100	1.49366700
H	-2.03345600	5.79198000	0.94392400
H	-0.88899600	1.70017300	1.64980200
H	-0.98300300	4.06315300	2.45555900
N	-1.87100000	2.06033300	-0.11692500
N	-1.86956300	-2.23512800	-0.45013000
N	-3.43636300	-0.21580200	1.15022000
C	-2.60455100	1.16702700	-2.81589400
H	-3.01028000	1.11325800	-3.83566300
H	-1.52691300	0.99244500	-2.87920200
H	-3.93032800	2.83792700	-2.41915100
H	-2.36067300	3.24480700	-3.04411000
C	-5.14716500	-0.66383300	-0.55782100
H	-4.86783400	-1.69450900	-0.80755200
H	-6.23853900	-0.62032600	-0.62136800
H	-3.40708600	-0.90119300	-3.89227900

C	-1.80381100	-1.93517500	-2.85614800
H	-0.96757200	-1.22685600	-2.87022000
H	-1.73777400	-2.54298400	-3.76348200
O	0.08475200	-0.20421900	-0.78913800

^{qnt}2

E (SMD/B3LYP-D3/BS1) = -2559.78127608 au
G (SMD/B3LYP-D3/BS1) = -2559.009589 au
E (SMD/B3LYP-D3/BS2//SMD/B3LYP-D3/BS1) = -5235.44916793 au
charge = 2 spin multiplicity = 5

Ni	1.77344600	-0.12469700	0.29934400
N	3.06537300	0.32530900	2.01575400
C	3.06284800	-0.79121900	3.01572500
H	3.88541000	-1.46766500	2.78048500
C	4.46017400	0.60289600	1.57387700
H	5.09815400	0.73626600	2.45943700
H	4.46300600	1.54232200	1.02097500
C	2.63402200	2.87828000	1.98518300
C	1.71241000	-2.67711500	2.00298600
C	1.46371100	-4.02085400	2.29725000
C	1.44737400	-4.95601000	1.26219000
H	1.28528800	-4.32176400	3.32488700
C	1.93310800	-3.17345900	-0.25714600
C	1.69081600	-4.52786400	-0.04364500
H	1.24899400	-6.00317400	1.47255600
H	2.11435500	-2.77846400	-1.25103800
H	1.68507200	-5.21937100	-0.87956900
C	4.69654500	-0.41155200	-0.79282200
C	5.68146000	-0.50167400	-1.78419300
C	3.03845000	-0.34672900	-2.42546200
C	5.31497000	-0.51835000	-3.12777700
H	6.72499100	-0.56573500	-1.49308000
C	3.96111500	-0.44996300	-3.45930700
H	1.97275000	-0.29957700	-2.62108600
H	6.07302500	-0.59011200	-3.90243800
H	3.62323900	-0.47465000	-4.48989400
C	2.20992400	3.06981100	0.54749500
C	2.36226900	4.34217200	-0.02712000
C	1.94771200	4.56741600	-1.33357000
H	2.80007900	5.14003600	0.56523900
C	1.24319800	2.28634800	-1.40905700
C	1.36338000	3.51396200	-2.04293600
H	2.06407600	5.54734400	-1.78750400
H	0.77324800	1.43762100	-1.88767500
H	1.00246900	3.63912200	-3.05831600
N	1.67292300	2.05555400	-0.15144000
N	1.94354700	-2.27660300	0.73801500

N	3.39258000	-0.31582200	-1.12697700
C	2.44060300	1.52188200	2.66323300
H	2.84782600	1.62818000	3.67874100
H	1.37459000	1.30408100	2.76025500
H	3.67369100	3.20951300	2.08573900
H	2.05255900	3.58908800	2.58727000
C	5.06950500	-0.48170200	0.66929000
H	4.80544800	-1.48076200	1.03609200
H	6.15788800	-0.40519600	0.74870600
H	3.27405500	-0.36872100	4.00634200
C	1.75993200	-1.60863700	3.06643200
H	0.89310200	-0.94811200	2.94469900
H	1.67983200	-2.07509600	4.05298200
O	-0.27932000	-0.60692900	0.73494000
Ni	-1.88797000	-0.01046000	-0.29515400
N	-3.03738400	0.60475600	-1.98304200
C	-3.15228200	-0.48028200	-3.00981500
H	-4.02467600	-1.08961700	-2.77313800
C	-4.39910400	1.04666700	-1.57164300
H	-4.98535900	1.28567900	-2.47085600
H	-4.29833600	1.96333000	-0.99014100
C	-2.27101800	3.07290100	-1.88215300
C	-1.94260200	-2.48179300	-2.05172600
C	-1.74170500	-3.83071000	-2.35925200
C	-1.77404000	-4.77774000	-1.33627300
H	-1.56175500	-4.12532400	-3.38846300
C	-2.21040900	-2.99372200	0.19627300
C	-2.01817800	-4.35365900	-0.02931100
H	-1.61314300	-5.82938400	-1.55614500
H	-2.38616300	-2.60744700	1.19361200
H	-2.04983900	-5.05311800	0.79941700
C	-4.79980000	-0.02923400	0.74192500
C	-5.79358300	-0.05261600	1.72842200
C	-3.15707700	-0.27275800	2.36930400
C	-5.43986600	-0.19433000	3.06770600
H	-6.83521000	0.03259100	1.43578700
C	-4.08982500	-0.31962900	3.39776100
H	-2.09619900	-0.37912300	2.56595200
H	-6.20467000	-0.21443800	3.83881500
H	-3.76182600	-0.44998400	4.42364500
C	-1.79082900	3.15424400	-0.45247100
C	-1.61194300	4.42950600	0.10808300
C	-1.16824100	4.55766900	1.41721800
H	-1.81969200	5.30564800	-0.49872400
C	-1.07347300	2.17282000	1.51844400
C	-0.88967100	3.39650400	2.14316500
H	-1.02835400	5.53982400	1.85951700

H	-0.82028300	1.24523400	2.01116700
H	-0.52169700	3.43467400	3.16287700
N	-1.53225700	2.04070000	0.25537400
N	-2.17472800	-2.08251500	-0.78654700
N	-3.49484600	-0.11666800	1.07465500
C	-2.27229800	1.72863200	-2.60704600
H	-2.68251500	1.92053900	-3.60908600
H	-1.24754500	1.37571600	-2.73747900
H	-3.26378300	3.53460800	-1.93768100
H	-1.62250500	3.72698900	-2.47869100
C	-5.17404200	0.03043800	-0.71947400
H	-5.07553100	-0.98054000	-1.13315300
H	-6.23585800	0.28402100	-0.79222700
H	-3.34426000	-0.01442000	-3.98515100
C	-1.92823000	-1.40444900	-3.10534500
H	-1.00284200	-0.82538700	-3.00598700
H	-1.91766000	-1.86643600	-4.09722800
O	0.08118600	-0.53606600	-0.73804400

^{s}2'
E (SMD/B3LYP-D3/BS1) = -2559.71265526 au
G (SMD/B3LYP-D3/BS1) = -2558.939216 au
E (SMD/B3LYP-D3/BS2//SMD/B3LYP-D3/BS1) = -5235.39779754 au
charge = 2 spin multiplicity = 1

Ni	2.12738600	0.11566200	0.02083800
N	3.30637500	-0.07407400	2.21948000
C	2.77641200	-1.23158100	2.97509400
H	3.55339500	-1.99945300	3.03578700
C	4.76176600	-0.19152300	2.06153300
H	5.23264000	-0.55969400	2.98714700
H	5.19767100	0.78760400	1.86057100
C	3.26659100	2.47809800	2.08464900
C	1.76741800	-2.56999400	1.06275100
C	1.78962100	-3.96780300	0.98813500
C	2.03578600	-4.60493100	-0.22419100
H	1.60273800	-4.54292100	1.88895100
C	2.23005500	-2.44392700	-1.22387200
C	2.24793800	-3.82517300	-1.36130200
H	2.04682200	-5.68922600	-0.28364300
H	2.40146700	-1.79419500	-2.07380300
H	2.42344900	-4.26864000	-2.33556100
C	5.06413600	-0.47545900	-0.46074000
C	6.21761800	-0.44837100	-1.25641300
C	3.89362800	0.63453500	-2.14053100
C	6.19463200	0.12792600	-2.52149400
H	7.12949200	-0.89037600	-0.86902000
C	4.99854200	0.67965600	-2.97810200

H	2.94767900	1.04867400	-2.46419300
H	7.08882800	0.14275300	-3.13759800
H	4.91272600	1.13550500	-3.95861400
C	2.49499500	2.93664200	0.86665100
C	2.29884000	4.31535100	0.69261100
C	1.64594300	4.80163400	-0.43415600
H	2.66959800	4.99653500	1.45172800
C	1.37013400	2.53731900	-1.13799500
C	1.16372900	3.88887700	-1.37119500
H	1.49992500	5.86945100	-0.56818500
H	0.97024300	1.78642500	-1.80568300
H	0.62190800	4.20622700	-2.25562000
N	2.04183300	2.06685900	-0.06266800
N	2.01325900	-1.82750900	-0.04469700
N	3.90908200	0.08069800	-0.90773000
C	2.85453800	1.19174500	2.82126900
H	3.23519500	1.27825800	3.85479100
H	1.76214500	1.14538100	2.87907300
H	4.32703800	2.43745400	1.81004000
H	3.19875000	3.28731000	2.81872200
C	5.12596400	-1.12941600	0.89709200
H	4.47292900	-2.00858300	0.91098000
H	6.14639500	-1.49430700	1.04672100
H	2.55553300	-0.93256400	4.01064700
C	1.51229800	-1.86943900	2.37049700
H	0.74728100	-1.10660700	2.21078100
H	1.13022900	-2.60154500	3.08960100
O	0.39673000	0.19050700	0.63702000
Ni	-2.10717700	0.07717300	-0.02023700
N	-3.45234800	-0.28937600	-2.18886500
C	-2.75396200	-1.31698500	-2.99170800
H	-3.39901500	-2.19734400	-3.07310300
C	-4.85208700	-0.67338500	-1.95903700
H	-5.26522100	-1.19883600	-2.83362100
H	-5.47060500	0.21455400	-1.81969600
C	-3.82371000	2.20326300	-1.86838600
C	-1.51892600	-2.52058400	-1.12223400
C	-1.36909500	-3.91162200	-1.07635600
C	-1.50903200	-4.59511400	0.12795900
H	-1.13505400	-4.44395500	-1.99234200
C	-1.94123500	-2.49326500	1.17741500
C	-1.79329200	-3.86920200	1.28496800
H	-1.38496100	-5.67325700	0.16537900
H	-2.17129400	-1.88245600	2.04257500
H	-1.89547100	-4.34991800	2.25188000
C	-4.95930000	-0.81604200	0.58747900
C	-6.08532100	-0.85069600	1.42162700

C	-3.87004800	0.54151500	2.13726000
C	-6.08986200	-0.17814300	2.63847200
H	-6.95416800	-1.41689400	1.10291000
C	-4.94869600	0.53170500	3.00952600
H	-2.96613300	1.08033800	2.39354900
H	-6.96259900	-0.21084900	3.28401700
H	-4.88591100	1.06814000	3.95021000
C	-2.89891500	2.80886000	-0.84047100
C	-2.81229400	4.20519800	-0.73408300
C	-2.02997000	4.78940100	0.25564000
H	-3.37426300	4.82045100	-1.42920700
C	-1.39684800	2.58849100	0.93502500
C	-1.30428300	3.96042000	1.11177900
H	-1.97381900	5.87024900	0.34633000
H	-0.79064800	1.89613900	1.50768400
H	-0.65437500	4.36112100	1.88142700
N	-2.19778400	2.02249100	0.00433900
N	-1.82209200	-1.83621500	0.00748100
N	-3.86109300	-0.10295400	0.94864300
C	-3.28820900	1.05798600	-2.75675900
H	-3.79598000	1.12753400	-3.73500500
H	-2.21855600	1.20833600	-2.93704500
H	-4.72990700	1.88145900	-1.34312500
H	-4.14265600	3.01245300	-2.53157800
C	-4.99398200	-1.57134500	-0.71830900
H	-4.21015800	-2.33494300	-0.73169000
H	-5.95095200	-2.09883200	-0.77524400
H	-2.59665300	-0.95067600	-4.01696900
C	-1.39591900	-1.76980900	-2.42215800
H	-0.74498500	-0.90727500	-2.26498600
H	-0.92819000	-2.42867400	-3.16179700
O	-0.37830000	0.29849000	-0.59708500

^{t}1

E (SMD/B3LYP-D3/BS1) = -2559.77074927 au
G (SMD/B3LYP-D3/BS1) = -2558.994695 au
E (SMD/B3LYP-D3/BS2//SMD/B3LYP-D3/BS1) = -5235.44386254 au
charge = 2 spin multiplicity = 3

N	-2.50156000	0.00915300	2.11378700
C	-2.47652900	1.31451000	2.87307500
H	-3.39799000	1.85659000	2.65962700
C	-3.92572300	-0.36745700	1.87927400
H	-4.46377400	-0.32293500	2.83553200
H	-3.95781400	-1.39752300	1.52731300
C	-2.13665400	-2.48781200	2.72645300
C	-1.51568300	2.98051000	1.28214200
C	-1.64022600	4.37298800	1.22747400

C	-1.90642400	4.98957200	0.00712400
H	-1.52782500	4.95513300	2.13658800
C	-1.88796100	2.82128300	-1.00205700
C	-2.03159600	4.19781300	-1.13583900
H	-2.00691200	6.06950600	-0.05286100
H	-1.95492400	2.16296300	-1.85896800
H	-2.22538000	4.63210800	-2.11110800
C	-4.35481700	0.14726900	-0.58769800
C	-5.40187600	0.05122000	-1.51184600
C	-2.81076900	-0.28432800	-2.27148400
C	-5.12932200	-0.21975800	-2.84915500
H	-6.42157800	0.19654000	-1.17046200
C	-3.80040800	-0.38256800	-3.23996300
H	-1.75818200	-0.37431800	-2.51337200
H	-5.93694500	-0.29563800	-3.57144200
H	-3.52768400	-0.58005700	-4.27127600
C	-1.93513300	-3.07000900	1.34856400
C	-2.10178800	-4.45350600	1.17515200
C	-1.88330500	-5.02193200	-0.07314400
H	-2.38884500	-5.06428600	2.02612800
C	-1.34529700	-2.83389800	-0.87344300
C	-1.48368100	-4.19200200	-1.12468900
H	-2.00443400	-6.09111500	-0.22256800
H	-0.99275100	-2.14783200	-1.63103100
H	-1.27097200	-4.58699700	-2.11267500
N	-1.58547900	-2.27277300	0.32700400
N	-1.64887700	2.22529400	0.17607100
N	-3.07415900	-0.04057300	-0.97166400
C	-1.80280200	-1.01757200	2.96832000
H	-2.06006400	-0.82227800	4.01599200
H	-0.73413900	-0.84099500	2.83728600
H	-3.16022400	-2.70621600	3.05402700
H	-1.49438500	-3.04904700	3.41830900
C	-4.64235000	0.50705800	0.84551100
H	-4.40005100	1.56711800	0.98676000
H	-5.71840500	0.41403400	1.01691200
H	-2.49414200	1.08079000	3.94203300
C	-1.29005900	2.23642900	2.56785100
H	-0.37253600	1.64609900	2.48514500
H	-1.18526100	2.94658500	3.39348900
N	2.50526100	0.04809700	-2.11211900
C	2.46017500	1.35349900	-2.86998500
H	3.36994300	1.91240800	-2.65013000
C	3.93514400	-0.30656400	-1.87799700
H	4.47206400	-0.25333900	-2.83445100
H	3.98329100	-1.33609300	-1.52636700
C	2.17911900	-2.45372300	-2.72670800

C	1.46051100	3.00182200	-1.28427800
C	1.54726300	4.39709900	-1.22883800
C	1.79763700	5.01997400	-0.00832400
H	1.41830400	4.97648700	-2.13752500
C	1.83899400	2.85144300	0.99961200
C	1.94609000	4.23123100	1.13393900
H	1.86839900	6.10222400	0.05226700
H	1.92447000	2.19466900	1.85606200
H	2.12954700	4.67006100	2.10917700
C	4.35599400	0.21491400	0.58899300
C	5.40395900	0.13433600	1.51358100
C	2.81766000	-0.23836900	2.27239600
C	5.13483000	-0.14009400	2.85089700
H	6.42162500	0.29414200	1.17257800
C	3.80827100	-0.32196000	3.24127000
H	1.76633700	-0.34374100	2.51379100
H	5.94323400	-0.20411700	3.57346900
H	3.53805300	-0.52304800	4.27254700
C	1.98616800	-3.03958700	-1.34905200
C	2.17269900	-4.42061400	-1.17635500
C	1.96290200	-4.99268700	0.07177900
H	2.46810700	-5.02683100	-2.02774200
C	1.39373800	-2.81299900	0.87330500
C	1.55184100	-4.16907500	1.12390500
H	2.09943100	-6.06009500	0.22062200
H	1.03154300	-2.13242700	1.63133500
H	1.34532900	-4.56756800	2.11179900
N	1.62534700	-2.24798800	-0.32698400
N	1.61410200	2.25003600	-0.17852500
N	3.07802800	0.00858600	0.97255300
C	1.82255500	-0.98862000	-2.96756400
H	2.07670100	-0.78821900	-4.01502300
H	0.75126100	-0.82898600	-2.83631000
H	3.20603900	-2.65620800	-3.05413400
H	1.54571000	-3.02437000	-3.41900500
C	4.63876900	0.57838300	-0.84422400
H	4.38131900	1.63474800	-0.98586300
H	5.71606700	0.50072400	-1.01538400
H	2.48878900	1.12131000	-3.93911400
C	1.25499700	2.25292600	-2.57068200
H	0.34793500	1.64605900	-2.49226300
H	1.14098000	2.96079200	-3.39703900
Ni	-1.42304500	0.04973900	0.29036100
O	-0.23634700	0.03404300	-1.12682600
Ni	1.42658100	0.07203600	-0.28997100
O	0.24029800	0.03843000	1.12705000

^{qnt}1

E (SMD/B3LYP-D3/BS1) = -2559.76444585 au

G (SMD/B3LYP-D3/BS1) = -2558.990991 au

E (SMD/B3LYP-D3/BS2//SMD/B3LYP-D3/BS1) = -5235.43229982 au

charge = 2 spin multiplicity = 5

Ni	1.47523400	-0.00448600	0.27532800
N	2.73131600	-0.00725500	2.15677700
C	2.74345600	-1.32518500	2.87169200
H	3.67031500	-1.84853200	2.63194800
C	4.12993300	0.41665300	1.88257100
H	4.71466100	0.36544400	2.81270100
H	4.12057900	1.45791700	1.56047100
C	2.20379600	2.45952200	2.73383100
C	1.75126200	-2.92905100	1.21293300
C	2.02150400	-4.29807700	1.10529500
C	2.25185400	-4.85880500	-0.14859900
H	2.04901200	-4.90728600	2.00298100
C	1.92466400	-2.68778100	-1.09405300
C	2.20164500	-4.03709400	-1.27619000
H	2.46151900	-5.92019500	-0.24537500
H	1.86499400	-2.00418000	-1.93209500
H	2.36349700	-4.42839400	-2.27500200
C	4.57296800	0.03175400	-0.62577100
C	5.64375900	0.26859500	-1.49729700
C	3.06176000	0.46326600	-2.33549700
C	5.39646700	0.60991500	-2.82466100
H	6.66018500	0.17620000	-1.12788600
C	4.07385700	0.69870500	-3.25910300
H	2.01490400	0.50070300	-2.61394900
H	6.22060200	0.79495000	-3.50768700
H	3.82551200	0.94440700	-4.28643100
C	1.90866100	3.02330600	1.36240300
C	2.01930000	4.41379600	1.19518200
C	1.73707900	4.99183800	-0.03447600
H	2.31936200	5.02344900	2.04216800
C	1.25474800	2.79968600	-0.84990600
C	1.33694400	4.16270700	-1.08632200
H	1.81521500	6.06650300	-0.17109800
H	0.92549600	2.11894000	-1.62414800
H	1.08507000	4.56018500	-2.06365600
N	1.54323100	2.22941900	0.34057700
N	1.70892800	-2.14496600	0.11687900
N	3.29637200	0.15142100	-1.04694300
C	2.00760300	0.97280200	3.02531100
H	2.32891200	0.83178200	4.06707000
H	0.94845800	0.70966200	2.96706000
H	3.21985000	2.76511100	3.01046200

H	1.55136700	2.98417000	3.44467900
C	4.82967700	-0.41384500	0.79281200
H	4.54646900	-1.47021700	0.87220900
H	5.90713900	-0.37091700	0.97616200
H	2.76461100	-1.13691400	3.95209800
C	1.56138900	-2.25173600	2.54253500
H	0.63307000	-1.67260000	2.53345100
H	1.49095400	-3.00926700	3.32904100
O	-0.32754300	-0.06561900	1.32483400
Ni	-1.55630300	-0.08487500	-0.29269500
N	-2.73426400	-0.19065400	-2.14713800
C	-2.58853200	-1.50294100	-2.86189900
H	-3.44165600	-2.13618200	-2.61464500
C	-4.18102300	0.07327200	-1.91598600
H	-4.72356800	-0.04308600	-2.86545000
H	-4.29675400	1.10967600	-1.59809100
C	-2.44748400	2.31874000	-2.69346200
C	-1.39416900	-2.96762500	-1.20672800
C	-1.39142700	-4.36143000	-1.08953800
C	-1.53030100	-4.94832300	0.16599500
H	-1.27771500	-4.96990300	-1.98073100
C	-1.66665800	-2.74867300	1.09337000
C	-1.67370800	-4.12476300	1.28363800
H	-1.52245400	-6.02928800	0.27092000
H	-1.75703800	-2.06122300	1.92392800
H	-1.77714400	-4.53292100	2.28342600
C	-4.62256400	-0.35978600	0.57303000
C	-5.72002200	-0.24242000	1.43524000
C	-3.18290300	0.23744700	2.28947600
C	-5.52334200	0.12708300	2.76310500
H	-6.71659600	-0.44764000	1.05741800
C	-4.22155800	0.36226200	3.20494300
H	-2.14978800	0.38055500	2.58150700
H	-6.36778000	0.22052300	3.44000700
H	-4.00796400	0.63478700	4.23333300
C	-2.16491400	2.86988100	-1.31515100
C	-2.35577700	4.24701600	-1.11574500
C	-2.08958200	4.81587100	0.12175800
H	-2.70467400	4.85481200	-1.94513200
C	-1.45664300	2.64200900	0.87875500
C	-1.61934800	3.99140200	1.14757900
H	-2.23121400	5.88060400	0.28294300
H	-1.06367000	1.96730100	1.62837200
H	-1.37248300	4.38273200	2.12856500
N	-1.73582000	2.07785900	-0.31573900
N	-1.53073200	-2.18211400	-0.11905900
N	-3.36816300	-0.09913100	0.99854000

C	-2.12978300	0.86170600	-3.02327100
H	-2.47666000	0.70538500	-4.05458800
H	-1.05200500	0.69546400	-2.99950700
H	-3.49221200	2.54430900	-2.93796700
H	-1.86041600	2.91434500	-3.40509900
C	-4.81867600	-0.82607600	-0.84667000
H	-4.43815200	-1.85137800	-0.92422900
H	-5.89229400	-0.88140800	-1.04826200
H	-2.64006500	-1.31852600	-3.94168400
C	-1.30357300	-2.28065200	-2.54168500
H	-0.44536900	-1.60090600	-2.55610100
H	-1.15416600	-3.03058800	-3.32412500
O	0.19653100	0.01210200	-1.27828800

^{sep}1

E (SMD/B3LYP-D3/BS1) = -2559.7715374 au
G (SMD/B3LYP-D3/BS1) = -2558.998080 au
E (SMD/B3LYP-D3/BS2//SMD/B3LYP-D3/BS1) = -5235.43743712 au
charge = 2 spin multiplicity = 7

Ni	1.72241200	0.13766000	-0.28255500
N	2.85998100	-0.30384700	-2.07395700
C	2.75059300	0.78669900	-3.09465600
H	3.57387100	1.48485600	-2.93956600
C	4.29290100	-0.54268500	-1.75017800
H	4.85609200	-0.68123500	-2.68408700
H	4.36392600	-1.47017500	-1.18123900
C	2.49148100	-2.86392500	-1.96551100
C	1.45094000	2.64409600	-2.00156100
C	1.16124600	3.98257900	-2.28254100
C	1.22331500	4.92587600	-1.25789900
H	0.89401200	4.27314300	-3.29368100
C	1.85407400	3.16118300	0.22801700
C	1.58194400	4.51041900	0.02523100
H	0.99584200	5.96905000	-1.45788000
H	2.12508800	2.77768700	1.20580600
H	1.64014400	5.20852100	0.85336300
C	4.68571300	0.52642500	0.56107700
C	5.73632300	0.66320200	1.47615800
C	3.15119000	0.44932900	2.30978100
C	5.46680700	0.69704200	2.84226300
H	6.75390300	0.74987100	1.10909700
C	4.14278300	0.59901500	3.27162200
H	2.10317800	0.37970400	2.57972100
H	6.27612300	0.80547500	3.55859600
H	3.87900900	0.63641500	4.32325300
C	2.18411000	-3.02035800	-0.49653500
C	2.38192300	-4.27185500	0.10846800

C	2.05315300	-4.45378700	1.44678000
H	2.78087700	-5.08963600	-0.48414400
C	1.34584700	-2.17529900	1.49440300
C	1.50878500	-3.38031100	2.15961900
H	2.20154300	-5.41882800	1.92268200
H	0.89750900	-1.29936300	1.94280600
H	1.21094000	-3.47534600	3.19845400
N	1.69257600	-1.98792900	0.20633400
N	1.79202900	2.25396700	-0.75705300
N	3.41253500	0.40060600	0.98982700
C	2.21012500	-1.52559200	-2.64653300
H	2.53302200	-1.64141600	-3.69047000
H	1.13300500	-1.33396700	-2.65567700
H	3.52595700	-3.17904100	-2.14243900
H	1.87677500	-3.59805900	-2.50366900
C	4.94609400	0.57587100	-0.92397300
H	4.62719400	1.55967600	-1.28860100
H	6.02644300	0.52490700	-1.08788700
H	2.89197000	0.34681700	-4.08994200
C	1.43064900	1.57102200	-3.05601500
H	0.58812400	0.89862900	-2.85469700
H	1.26391700	2.02309500	-4.03855600
O	-0.31199400	0.45833400	-0.96747100
Ni	-1.81497800	0.02020000	0.22858000
N	-2.88152900	-0.42752300	2.03588400
C	-2.97487900	0.73990900	2.97500200
H	-3.87561700	1.30483100	2.73518100
C	-4.24826900	-0.93509200	1.72690100
H	-4.80219000	-1.07104500	2.66697000
H	-4.13979400	-1.91638800	1.26301700
C	-2.05844500	-2.91620500	2.15681400
C	-1.90742100	2.75460300	1.89235500
C	-1.78878100	4.12185400	2.15919600
C	-1.93665500	5.04002800	1.12144400
H	-1.58773700	4.45333200	3.17307600
C	-2.30774800	3.19460800	-0.35236400
C	-2.20770400	4.56868600	-0.16344100
H	-1.84267200	6.10514900	1.31233000
H	-2.49856900	2.77468400	-1.33314600
H	-2.32707100	5.24426200	-1.00378900
C	-4.75531500	-0.20118300	-0.68794900
C	-5.78142300	-0.34752300	-1.62887400
C	-3.17282300	-0.18186700	-2.39175900
C	-5.47570100	-0.40731100	-2.98597600
H	-6.80997000	-0.40872600	-1.28830300
C	-4.14050000	-0.31242000	-3.37977700
H	-2.12066800	-0.08936400	-2.63759400

H	-6.26582800	-0.52133800	-3.72251300
H	-3.84948500	-0.33821300	-4.42457200
C	-1.61145000	-3.19671800	0.74110100
C	-1.36566200	-4.53920800	0.40378200
C	-0.92401800	-4.86809200	-0.86989900
H	-1.51243800	-5.30809400	1.15656700
C	-0.97504000	-2.53580200	-1.38128800
C	-0.71660700	-3.83589700	-1.78822100
H	-0.72926700	-5.90264300	-1.13742900
H	-0.78956400	-1.69547800	-2.03877100
H	-0.35400000	-4.02851900	-2.79266800
N	-1.43437000	-2.21267800	-0.15205100
N	-2.16429300	2.30897300	0.64627900
N	-3.46466800	-0.13903500	-1.07627700
C	-2.10881400	-1.50105300	2.74601900
H	-2.51552300	-1.61566200	3.76104200
H	-1.08609800	-1.13454600	2.82024600
H	-3.03045100	-3.40348900	2.29881000
H	-1.36022700	-3.46002400	2.80599200
C	-5.07455500	-0.05722900	0.77749900
H	-4.98469700	1.00348000	1.04109600
H	-6.12616000	-0.31943500	0.92672400
H	-3.11491500	0.33792400	3.98637500
C	-1.78860000	1.71441500	2.97412400
H	-0.83972400	1.17908200	2.85479500
H	-1.76439400	2.21671600	3.94619000
O	0.16663100	0.48293700	0.86138500

^{t}21

E (SMD/B3LYP-D3/BS1) = -1512.2138703 au
G (SMD/B3LYP-D3/BS1) = -1511.749403 au
E (SMD/B3LYP-D3/BS2//SMD/B3LYP-D3/BS1) = -2850.15670734 au
charge = 0 spin multiplicity = 3

Ni	-0.05329800	-0.17920500	0.39461900
N	-0.01721600	-2.18163100	1.25120000
C	-1.34336600	-2.51667000	1.86862700
H	-1.93687800	-3.06943700	1.13857800
C	0.32877200	-3.21484500	0.23848600
H	0.18289300	-4.21441800	0.67275100
H	1.38570500	-3.11449200	-0.00744900
C	2.46757700	-2.09527900	1.98897300
C	-2.90813200	-0.68362000	1.17733000
C	-4.29553800	-0.51894200	1.17638200
C	-4.92460000	-0.02207000	0.03512000
H	-4.86764300	-0.78784400	2.05878900
C	-2.77410700	0.10222200	-1.00599700
C	-4.15104100	0.28426900	-1.08570200

H	-6.00206100	0.11469700	0.01808800
H	-2.12158800	0.33534700	-1.84012300
H	-4.59776000	0.66202000	-1.99942900
C	0.06808900	-2.07343800	-2.03746100
C	0.29702000	-2.40747000	-3.37721000
C	0.74667300	0.11436500	-2.44731400
C	0.76109900	-1.43916700	-4.26425600
H	0.10622600	-3.42210300	-3.71184800
C	0.98679100	-0.14629000	-3.79124100
H	0.87739500	1.10824000	-2.03390600
H	0.94006100	-1.68912200	-5.30609100
H	1.33838100	0.64713300	-4.44234300
C	2.99058200	-0.87975300	1.26145200
C	4.37902600	-0.69482800	1.16730300
C	4.88307500	0.44052200	0.54465500
H	5.04377000	-1.44028900	1.59403500
C	2.62373400	1.13787000	0.17839600
C	3.98290200	1.38756200	0.04793300
H	5.95547100	0.59460300	0.46268500
H	1.86806000	1.84335400	-0.14749300
H	4.32331600	2.30274600	-0.42580100
N	2.13132700	0.01641500	0.74827500
N	-2.16647900	-0.35749700	0.09900100
N	0.30470800	-0.82253100	-1.58571400
C	0.98675600	-2.14460300	2.36165700
H	0.85483000	-3.04369500	2.98050000
H	0.73374800	-1.27822000	2.98100900
H	2.76309700	-2.99740500	1.44110900
H	3.00937200	-2.16083700	2.94137600
C	-0.47269400	-3.09498900	-1.06778800
H	-1.52048100	-2.85307900	-0.85586700
H	-0.47191900	-4.07314400	-1.55744100
H	-1.17444700	-3.19328300	2.71508400
C	-2.15838800	-1.29621000	2.33206900
H	-1.49507600	-0.55186600	2.79196700
H	-2.86813000	-1.61631800	3.10065900
O	-0.14154400	1.60434000	0.92676000
C	-0.79379700	3.05911700	-0.91716000
C	-1.05758200	2.66079800	0.51001100
C	-0.89070600	3.86576100	1.39311100
C	-0.04726800	4.90297500	1.04554500
C	0.51341300	5.02581600	-0.25299600
C	0.05104500	4.11015300	-1.23012200
H	-2.07620200	2.24238400	0.61580800
H	-1.33437200	3.83215700	2.39047700
H	0.15459500	5.68714700	1.78140600
H	1.12657700	5.88245700	-0.52508700

H	0.32848400	4.26882100	-2.27676800
H	-1.16311400	2.40465000	-1.70622300

^{t}22

E (SMD/B3LYP-D3/BS1) = -1279.65924913 au
G (SMD/B3LYP-D3/BS1) = -1279.281214 au
E (SMD/B3LYP-D3/BS2//SMD/B3LYP-D3/BS1) = -2617.50026489 au
charge = 2 spin multiplicity = 3

Ni	-0.06434900	-0.33253700	-0.45527100
N	0.11788100	-1.29543800	1.44852500
C	1.36629500	-2.13630900	1.54745200
H	2.10727600	-1.58410600	2.12572100
C	0.12458100	-0.31023100	2.56840300
H	0.37681300	-0.84580800	3.49147600
H	-0.87671500	0.10071800	2.68472500
C	-2.44003400	-1.53735900	1.51213500
C	2.62422100	-1.37690900	-0.49556600
C	3.98167200	-1.35922300	-0.82197100
C	4.53040300	-0.26042400	-1.47749200
H	4.59195700	-2.21634300	-0.55902400
C	2.36675500	0.75659300	-1.45028600
C	3.70563600	0.81617300	-1.80429300
H	5.58518900	-0.24627700	-1.73365600
H	1.68634300	1.56463700	-1.68334600
H	4.08176200	1.69122700	-2.32220500
C	0.53979100	1.90201500	1.43156900
C	0.60468300	3.25310700	1.78371200
C	-0.53043500	2.47371000	-0.56600200
C	0.09102100	4.22588500	0.93335200
H	1.06279200	3.52308000	2.72914200
C	-0.48879700	3.82670500	-0.27078900
H	-0.95795100	2.12713300	-1.49715200
H	0.14154000	5.27578900	1.20471500
H	-0.90243500	4.54019200	-0.97450900
C	-2.89441500	-0.88540400	0.23477800
C	-4.26470100	-0.82321200	-0.04997800
C	-4.71611700	-0.25564400	-1.23402000
H	-4.96142600	-1.23905500	0.67022000
C	-2.43904800	0.14281700	-1.82525600
C	-3.78000800	0.22652200	-2.15133700
H	-5.77880900	-0.21048000	-1.45080200
H	-1.67197900	0.46852100	-2.51777600
H	-4.07295200	0.64790600	-3.10617200
N	-2.01169100	-0.36426000	-0.64285200
N	1.85060900	-0.30637300	-0.79799600
N	-0.03407600	1.52816500	0.26389200
C	-1.06761900	-2.20496400	1.52729700

H	-0.99281300	-2.76988800	2.46473100
H	-0.97990700	-2.90929800	0.69672200
H	-2.54424800	-0.81571900	2.32969800
H	-3.16027800	-2.32933400	1.74059800
C	1.10047400	0.84471900	2.33880100
H	2.05257700	0.48673800	1.92897200
H	1.33001600	1.30038400	3.30499300
H	1.11659100	-3.03557900	2.11820000
C	1.99367500	-2.54274300	0.21524300
H	1.24875700	-3.02014800	-0.42739000
H	2.76875900	-3.28358600	0.42770800
O	-0.16954300	-1.68740700	-1.55918800

^{t}23

E (SMD/B3LYP-D3/BS1) = -1511.9894053 au
G (SMD/B3LYP-D3/BS1) = -1511.517938 au
E (SMD/B3LYP-D3/BS2//SMD/B3LYP-D3/BS1) = -2849.91085169 au
charge = 2 spin multiplicity = 3

Ni	0.37358000	0.01896800	-0.09631000
N	0.38537100	-0.00409000	1.97730800
C	0.12262400	-1.37466300	2.53048600
H	1.06066300	-1.92802300	2.53602900
C	1.70480000	0.49330200	2.47608200
H	1.69692400	0.44493000	3.57305000
H	1.79676500	1.54002200	2.18610100
C	-0.59084600	2.38837400	2.11554100
C	-0.38848900	-2.81959000	0.49514600
C	-0.60572700	-4.16722800	0.19588800
C	-0.11087800	-4.70292000	-0.99121200
H	-1.16014000	-4.78325200	0.89606900
C	0.79518900	-2.55125100	-1.49959200
C	0.60873300	-3.87993300	-1.85836100
H	-0.27943300	-5.74812300	-1.23280000
H	1.34636600	-1.86762100	-2.13560200
H	1.01778100	-4.25197100	-2.79130300
C	3.36619500	0.07932500	0.54335900
C	4.72362900	0.23599800	0.24073600
C	2.81990000	0.41241700	-1.69726900
C	5.12228200	0.48140700	-1.06974100
H	5.45459200	0.16023300	1.03909800
C	4.14866800	0.56586600	-2.06591900
H	2.02745300	0.46522800	-2.43539000
H	6.17426700	0.60303600	-1.31014100
H	4.40519800	0.74781900	-3.10381600
C	-0.57135900	2.84181600	0.67478600
C	-1.05190500	4.12197100	0.36648300
C	-0.99340200	4.59731600	-0.93821800

H	-1.46791100	4.73278100	1.16117600
C	-0.00632700	2.51646300	-1.56225400
C	-0.45466700	3.77535900	-1.93015500
H	-1.36587000	5.58839000	-1.17945600
H	0.39358500	1.83125600	-2.30126800
H	-0.39076800	4.09244000	-2.96523400
N	-0.05556400	2.05363400	-0.29275400
N	0.30584500	-2.03145500	-0.35729800
N	2.43078000	0.18169100	-0.42573100
C	-0.72246100	0.89827700	2.43601000
H	-0.79903400	0.81162700	3.52785500
H	-1.64683900	0.51431800	2.00244400
H	0.28974200	2.81180100	2.61325400
H	-1.44821800	2.86794700	2.59977900
C	2.93501300	-0.25566600	1.94927700
H	2.79668500	-1.34136700	2.01237200
H	3.76339300	-0.02266500	2.62449900
H	-0.19590600	-1.26157900	3.57358400
C	-0.91879100	-2.19935000	1.76448300
H	-1.80311600	-1.59631900	1.53434400
H	-1.25653700	-3.00160400	2.42667000
O	-1.82182600	-0.11927700	-0.34738600
C	-2.69088300	0.48854600	-1.35457100
C	-2.60299000	-1.00824700	-1.20902300
C	-3.72023400	-1.69997800	-0.54983300
C	-4.70374100	-0.98194600	0.04164900
C	-4.78521700	0.46478400	-0.09514800
C	-3.88336900	1.16350800	-0.82382400
H	-1.97519400	-1.56712900	-1.89726900
H	-3.73961400	-2.78520500	-0.57598700
H	-5.51440200	-1.49129400	0.55568700
H	-5.65214900	0.96784200	0.32473600
H	-4.02249000	2.21582800	-1.05267700
H	-2.13095500	0.97158100	-2.14954500

^{s}24

E (SMD/B3LYP-D3/BS1) = -1279.94652701 au

G (SMD/B3LYP-D3/BS1) = -1279.574191 au

E (SMD/B3LYP-D3/BS2//SMD/B3LYP-D3/BS1) = -2617.80264774 au

charge = 0 spin multiplicity = 1

Ni	0.16369500	-0.37416700	-0.76485900
N	0.13951500	-1.62770200	0.94051000
C	1.49208300	-2.14065800	1.31152400
H	1.97110100	-1.41597200	1.97004600
C	-0.50043800	-1.04911600	2.16257500
H	-0.48541000	-1.80860100	2.95826900
H	-1.54133400	-0.83111200	1.93049500

C	-2.15742800	-2.61443500	0.36118200
C	2.97846400	-1.06743900	-0.36899400
C	4.35688700	-0.83538700	-0.43014900
C	4.82864000	0.41750500	-0.81201400
H	5.04019800	-1.63862100	-0.17386500
C	2.54652900	1.11096700	-1.06832600
C	3.90168200	1.41265100	-1.12816700
H	5.89617500	0.61486100	-0.85649700
H	1.76563400	1.80170700	-1.35407400
H	4.21678300	2.40890400	-1.42200200
C	-0.20297700	1.48314000	1.86922600
C	0.80630100	2.34109000	1.41412300
C	-1.82940500	2.81644800	0.92764300
C	0.45861200	3.47036500	0.67524500
H	1.84628200	2.10851300	1.61910700
C	-0.89042100	3.71468700	0.41659400
H	-2.89180600	2.97634400	0.75376400
H	1.22766900	4.14067300	0.30090800
H	-1.21166900	4.57770200	-0.15843000
C	-2.68692200	-1.39056700	-0.34585200
C	-4.08063800	-1.24627800	-0.43443900
C	-4.62903100	-0.13561100	-1.06001100
H	-4.71373400	-2.01771800	-0.00599900
C	-2.39207900	0.60594000	-1.49603000
C	-3.75857200	0.81130700	-1.60637900
H	-5.70672100	-0.01176000	-1.12421000
H	-1.61769900	1.23445600	-1.93648700
H	-4.12918500	1.69924600	-2.10943000
N	-1.85225800	-0.46841200	-0.86666600
N	2.08913800	-0.10418900	-0.69195600
N	-1.50620000	1.72964400	1.64032800
C	-0.64897900	-2.80465400	0.44854100
H	-0.47416200	-3.66048800	1.11597900
H	-0.25136100	-3.06937000	-0.53424900
H	-2.59948900	-2.64336400	1.36375800
H	-2.55276300	-3.50131300	-0.15109700
C	0.12080800	0.25264400	2.68943200
H	1.20351800	0.16949100	2.81494000
H	-0.29547900	0.39593500	3.69588800
H	1.37293200	-3.07012200	1.88427800
C	2.41582600	-2.37359900	0.11092400
H	1.87817800	-2.87459300	-0.70254400
H	3.23135400	-3.03493200	0.41808700
O	0.17156500	0.56843200	-2.23199700

^{t}25

E (SMD/B3LYP-D3/BS1) = -1587.36150211 au

G (SMD/B3LYP-D3/BS1) = -1586.894797 au
E (SMD/B3LYP-D3/BS2//SMD/B3LYP-D3/BS1) = -2925.33656765 au
0 3

O	1.34966700	1.94262000	-1.17348900
Ni	-0.27891200	-0.12053700	-0.27147100
N	-1.67923300	-1.21934200	-1.45889600
C	-1.03255800	-2.37254400	-2.16583200
H	-1.11946800	-3.25881000	-1.53673000
C	-2.81306000	-1.71277400	-0.63179700
H	-3.47784400	-2.32318600	-1.26056300
H	-3.38349800	-0.84892600	-0.28955400
C	-3.03033900	0.90799100	-2.07090200
C	1.36004800	-2.44094200	-1.34724300
C	2.41170000	-3.35905500	-1.41827400
C	3.20567300	-3.58632000	-0.29409600
H	2.59694700	-3.88840700	-2.34770600
C	1.86158100	-1.99426600	0.87818200
C	2.92369100	-2.89314500	0.88306700
H	4.02812100	-4.29480600	-0.33694400
H	1.60464600	-1.42098400	1.76249600
H	3.51160000	-3.03563500	1.78381500
C	-1.97293600	-1.72451100	1.81005500
C	-2.49576500	-2.01744200	3.07583200
C	-0.59784400	-0.09770900	2.73657500
C	-2.04180400	-1.32169500	4.19330800
H	-3.24869900	-2.79319300	3.17305400
C	-1.06122100	-0.34396200	4.02327400
H	0.17340900	0.64289000	2.55339500
H	-2.44117100	-1.54333100	5.17882400
H	-0.66139000	0.21728000	4.86117200
C	-2.47201600	1.96587300	-1.14766700
C	-2.97529500	3.27176200	-1.24222600
C	-2.53600600	4.25417900	-0.36047700
H	-3.71227000	3.50182100	-2.00555500
C	-1.12245100	2.60190700	0.62464500
C	-1.58794700	3.91061800	0.60322100
H	-2.92276700	5.26719900	-0.42662100
H	-0.36994100	2.29841100	1.33943600
H	-1.20359100	4.63547700	1.31326600
N	-1.55169600	1.64358300	-0.21800200
N	1.10069900	-1.77086800	-0.20463300
N	-1.04521100	-0.75705700	1.65046100
C	-2.14225600	-0.25872100	-2.50951700
H	-2.71710900	-0.82000100	-3.26088100
H	-1.24047700	0.13462800	-2.98279400
H	-3.96519500	0.53415300	-1.63393000
H	-3.33452800	1.40590000	-2.99860900

C	-2.39705100	-2.52737300	0.60415000
H	-1.58209000	-3.21841200	0.35795000
H	-3.24881000	-3.15169200	0.88953200
H	-1.60207800	-2.57837400	-3.08127300
C	0.45035700	-2.16764700	-2.51708800
H	0.62467500	-1.14111500	-2.85815200
H	0.70593700	-2.84491400	-3.33779700
O	0.74223700	0.72143800	-1.72298800
C	2.43659000	2.82757800	0.78804300
C	2.14440500	1.57940400	0.04215400
C	3.38194600	0.81742000	-0.26805400
C	4.64585000	1.33134000	-0.05528500
C	4.86825500	2.58317400	0.56513800
C	3.72611700	3.29531300	0.98289300
H	1.58985500	3.41821900	1.13230900
H	1.38091600	0.92867700	0.58218000
H	3.25802100	-0.14723000	-0.75231400
H	5.50645000	0.73463700	-0.36736900
H	5.87198200	2.95963700	0.74492200
H	3.85475000	4.24775300	1.50243500

^{t}26

E (SMD/B3LYP-D3/BS1) = -1587.10143657 au
G (SMD/B3LYP-D3/BS1) = -1586.627833 au
E (SMD/B3LYP-D3/BS2//SMD/B3LYP-D3/BS1) = -2925.05644442 au
charge = 2 spin multiplicity = 3

O	1.52728200	1.86538100	-1.19689300
Ni	-0.35635100	-0.12817300	-0.22545400
N	-1.78320800	-0.97193600	-1.52056400
C	-1.25212400	-2.18492900	-2.22691600
H	-1.43991400	-3.05741600	-1.60169000
C	-3.02343300	-1.33245400	-0.77241500
H	-3.72302600	-1.81561600	-1.46833800
H	-3.48571700	-0.40969500	-0.42183000
C	-2.79848500	1.33877000	-2.10672500
C	1.12402700	-2.49672800	-1.38293800
C	2.15083700	-3.43967700	-1.48488100
C	2.93055800	-3.73570600	-0.36757800
H	2.32934800	-3.93247700	-2.43521300
C	1.61435400	-2.17214300	0.87109900
C	2.65453200	-3.09443500	0.84057100
H	3.73546200	-4.46160500	-0.43780400
H	1.35891800	-1.63974100	1.78134700
H	3.22757800	-3.29821300	1.73882000
C	-2.31598500	-1.56998100	1.70007700
C	-2.89911900	-1.86171300	2.93901900
C	-0.77136300	-0.17531600	2.74237000

C	-2.38814900	-1.28955600	4.10074900
H	-3.74531400	-2.53989100	2.97983300
C	-1.29151800	-0.43183500	4.00396900
H	0.08549500	0.47780700	2.61506800
H	-2.83511900	-1.51244800	5.06514300
H	-0.84761000	0.03063100	4.87892600
C	-2.14539500	2.27125400	-1.11263500
C	-2.49542100	3.62952400	-1.13760500
C	-1.95409600	4.50853300	-0.20689000
H	-3.19284100	3.98083700	-1.89159200
C	-0.74672000	2.66153100	0.69997500
C	-1.05449500	4.01335600	0.73907100
H	-2.22270100	5.56073900	-0.22222300
H	-0.03518200	2.23821800	1.39952400
H	-0.59435700	4.65459100	1.48334100
N	-1.28060000	1.80236400	-0.19156800
N	0.87637800	-1.87254100	-0.21111500
N	-1.27178800	-0.71862600	1.61498500
C	-2.06331100	0.07435300	-2.55703500
H	-2.67748900	-0.38142800	-3.34581500
H	-1.09881700	0.35004800	-2.98710600
H	-3.79824900	1.08843200	-1.73123200
H	-2.97968600	1.91086500	-3.02360900
C	-2.81089200	-2.24545700	0.44421000
H	-2.12223700	-3.06463700	0.20622400
H	-3.77223500	-2.71780400	0.66647000
H	-1.82823600	-2.32081300	-3.15034100
C	0.24697400	-2.14143400	-2.55818700
H	0.53168000	-1.15086400	-2.92881100
H	0.43571800	-2.85380700	-3.36675100
O	0.96390700	0.58315500	-1.60289400
C	2.95661900	2.83194700	0.46784000
C	2.47857600	1.62320600	-0.21492800
C	3.42731300	0.51058700	-0.38148600
C	4.61096800	0.51744900	0.32048900
C	4.97530300	1.65984000	1.05675300
C	4.15755400	2.80744300	1.13312600
H	2.29075900	3.68893600	0.49701900
H	1.96267200	1.16997700	0.70462900
H	3.10218700	-0.32742400	-0.98541900
H	5.28268100	-0.33314600	0.27400000
H	5.92598700	1.66250000	1.58243900
H	4.49046500	3.67059400	1.69924300

^{s}27

E (SMD/B3LYP-D3/BS1) = -1204.71657817 au

G (SMD/B3LYP-D3/BS1) = -1204.346742 au

E (SMD/B3LYP-D3/BS2//SMD/B3LYP-D3/BS1) = -2542.54299504 au
charge = 0 spin multiplicity = 1

Ni	-0.00108200	-0.00749800	-0.41834800
N	0.00968500	-0.01300200	2.00526200
C	0.20881700	1.37564500	2.44709300
H	-0.76341400	1.87670400	2.46798300
C	-1.29149800	-0.53783500	2.44609700
H	-1.49776900	-0.22698000	3.48444100
H	-1.24153000	-1.63066700	2.45180800
C	1.33262900	-2.09876800	1.51263900
C	0.47388300	2.77847600	0.32511800
C	0.35510400	4.16387100	0.18998800
C	-0.27863300	4.72140700	-0.92117000
H	0.77277800	4.80207500	0.96395000
C	-0.65174000	2.47897200	-1.69342500
C	-0.78974700	3.84453800	-1.88561900
H	-0.36614300	5.79794000	-1.03383400
H	-1.04571100	1.77126000	-2.41637900
H	-1.29184200	4.21034300	-2.77663600
C	-2.65415700	-0.97913100	0.32672700
C	-3.80270100	-1.76164900	0.18567100
C	-1.84555000	-1.78941200	-1.70346400
C	-3.97997000	-2.57505400	-0.93431500
H	-4.56163300	-1.72013000	0.96224000
C	-2.96748700	-2.57853400	-1.90140500
H	-1.03730200	-1.77686800	-2.42818900
H	-4.87477800	-3.17913500	-1.05116900
H	-3.04129200	-3.18579000	-2.79897300
C	2.18639000	-1.80161100	0.30851400
C	3.44937800	-2.38140100	0.16665800
C	4.24216300	-2.10350100	-0.94765000
H	3.80200700	-3.06049000	0.93816500
C	2.47196500	-0.67169600	-1.71009500
C	3.72693800	-1.22497000	-1.90860600
H	5.22119300	-2.55833600	-1.06543900
H	2.04735400	0.02104000	-2.43017800
H	4.28867600	-0.96749300	-2.80194000
N	1.68513600	-0.92934600	-0.62396400
N	-0.03855700	1.91684400	-0.61068100
N	-1.65443600	-0.99090000	-0.61222400
C	1.11519200	-0.88056900	2.43953300
H	0.94458800	-1.22989500	3.47204700
H	2.03504600	-0.28887400	2.45877200
H	0.35339600	-2.46858600	1.18036700
H	1.80633600	-2.89765200	2.09313900
C	-2.46282200	-0.10234900	1.53588800
H	-2.29099500	0.93211500	1.20990700

H	-3.38468000	-0.11088800	2.12712300
H	0.59572000	1.39710400	3.48004800
C	1.15658300	2.17715800	1.52504800
H	1.96490800	1.51496300	1.18753400
H	1.61416300	2.98147700	2.11085700

[t]8[i]

E (SMD/B3LYP-D3/BS1) = -1337.3344251 au
G (SMD/B3LYP-D3/BS1) = -1336.921738 au
E (SMD/B3LYP-D3/BS2//SMD/B3LYP-D3/BS1) = -2675.19221604 au
charge = 2 spin multiplicity = 3

Ni	-0.03876000	-0.18391700	-0.13200100
N	0.08012800	-0.09286800	1.97238800
C	1.31919200	-0.76078200	2.50990700
H	2.08769000	-0.00052700	2.64966500
C	0.06242200	1.32991700	2.43165600
H	0.23364500	1.34445600	3.51563700
H	-0.93007400	1.73862600	2.24562100
C	-2.49599800	-0.27595700	2.20329000
C	2.65334800	-1.34324300	0.43930000
C	3.99348600	-1.66381600	0.21075200
C	4.65483200	-1.12201900	-0.89006200
H	4.50556700	-2.33045900	0.89683700
C	2.63301700	0.02175000	-1.45505400
C	3.96266500	-0.26065200	-1.74245500
H	5.69656500	-1.36538500	-1.07703600
H	2.04609900	0.68657700	-2.07947200
H	4.43669000	0.18631900	-2.60951900
C	0.64874100	2.75017000	0.38701900
C	0.78163100	4.10257800	0.05726500
C	-0.23649700	2.30793500	-1.72357900
C	0.39626800	4.55161100	-1.20309600
H	1.18750600	4.78933700	0.79273400
C	-0.12159400	3.63407400	-2.11788700
H	-0.62808000	1.55552300	-2.39777900
H	0.49752300	5.60022100	-1.46661200
H	-0.43193500	3.93180800	-3.11351700
C	-3.00009300	-0.28727300	0.78078300
C	-4.38130600	-0.35482300	0.54877200
C	-4.87162200	-0.35949900	-0.75135100
H	-5.05622800	-0.40866200	1.39701700
C	-2.61326300	-0.25664400	-1.51516900
C	-3.96489300	-0.31645100	-1.81328700
H	-5.94054200	-0.41027800	-0.93610800
H	-1.86799200	-0.25120300	-2.30404500
H	-4.29220300	-0.33704600	-2.84701500
N	-2.13560500	-0.22183500	-0.25102800

N	1.99557700	-0.50580400	-0.39396900
N	0.13474400	1.87286600	-0.50258600
C	-1.10660700	-0.84590400	2.49826500
H	-1.01256300	-0.90556700	3.59025500
H	-1.03155200	-1.86199800	2.09972100
H	-2.59411700	0.74089500	2.60034900
H	-3.19059200	-0.87720600	2.80022000
C	1.09122600	2.22947900	1.73032700
H	2.04638300	1.70624600	1.60166800
H	1.29370900	3.08176100	2.38463900
H	1.08335200	-1.16315100	3.50103800
C	1.89317000	-1.87629400	1.62746900
H	1.09210700	-2.54484600	1.29440200
H	2.57126100	-2.47386100	2.24315900
C	-0.37990200	-3.37827900	-1.26224400
C	-0.54187600	-4.70339800	-1.83523700
H	0.37733900	-5.28004500	-1.69092400
H	-1.37497000	-5.21607200	-1.34396400
H	-0.75125000	-4.61529700	-2.90626200
N	-0.25000700	-2.32455800	-0.80151600

^{t}8^{ii}

E (SMD/B3LYP-D3/BS1) = -1337.33326828 au
G (SMD/B3LYP-D3/BS1) = -1336.921602 au
E (SMD/B3LYP-D3/BS2//SMD/B3LYP-D3/BS1) = -2675.19098858 au
charge = 2 spin multiplicity = 3

Ni	0.00187400	0.01618300	-0.41383200
N	-0.07615500	-2.09989900	-0.35804900
C	1.22285300	-2.73925100	-0.76862400
H	1.83237300	-2.89437900	0.12165400
C	-0.44017300	-2.55335600	1.01620600
H	-0.36476800	-3.64753700	1.05917800
H	-1.47936500	-2.28173300	1.20085300
C	-2.55971700	-2.22114500	-1.06888600
C	2.82702400	-0.84883700	-1.08595200
C	4.21928100	-0.76687000	-1.16003000
C	4.88886000	0.20454900	-0.41869900
H	4.76148300	-1.46583400	-1.78827400
C	2.76748500	0.93653300	0.40935600
C	4.14995400	1.07009500	0.39022000
H	5.97111500	0.28111900	-0.46626400
H	2.14355100	1.59114700	1.00733600
H	4.62855500	1.83600600	0.99085600
C	0.00311600	-0.54695300	2.55739200
C	-0.14528100	-0.24421500	3.91486300
C	-0.52790100	1.65418500	1.99919500
C	-0.48928800	1.04571400	4.30872500

H	0.01425400	-1.02585500	4.65007000
C	-0.68154600	2.01992500	3.32910900
H	-0.65802800	2.37883500	1.20526900
H	-0.60371200	1.28647500	5.36143000
H	-0.94423800	3.04149500	3.58124200
C	-3.01310300	-0.78648800	-0.97989700
C	-4.39190800	-0.53697500	-1.04975200
C	-4.87407900	0.76196000	-0.97742100
H	-5.06948300	-1.37723800	-1.16474300
C	-2.60674800	1.48792700	-0.78472400
C	-3.95377200	1.80389800	-0.84821900
H	-5.94043300	0.96069800	-1.02914300
H	-1.86874100	2.27152900	-0.68942800
H	-4.26611100	2.84137700	-0.79771700
N	-2.12663600	0.22194100	-0.83551800
N	2.12159800	0.00358600	-0.31379500
N	-0.19816700	0.40362400	1.61590600
C	-1.09847600	-2.51801200	-1.37365800
H	-1.02110000	-3.60411600	-1.50965800
H	-0.81382500	-2.04464500	-2.31820700
H	-2.88358400	-2.74642100	-0.16291700
H	-3.12786700	-2.69140300	-1.88045400
C	0.42401100	-1.92868600	2.12267700
H	1.47698900	-1.88730600	1.82028900
H	0.38109700	-2.58957100	2.99258200
H	0.99926900	-3.72973800	-1.17874500
C	2.03546800	-1.92111700	-1.78419100
H	1.37123300	-1.47463700	-2.53601600
H	2.71503700	-2.59294100	-2.31527400
C	0.73026800	2.91620000	-1.77717700
C	1.21857200	4.11136300	-2.43988900
H	1.06729400	4.01700300	-3.52008900
H	0.67061600	4.98428700	-2.07052200
H	2.28614000	4.23331500	-2.22954400
N	0.34227400	1.96707100	-1.24143400

$^{t}8^{iii}$

E (SMD/B3LYP-D3/BS1) = -1470.11129450 au
G (SMD/B3LYP-D3/BS1) = -1469.659037 au
E (SMD/B3LYP-D3/BS2//SMD/B3LYP-D3/BS1) = -2808.02236531 au
charge = 2 spin multiplicity = 3

Ni	-0.02303900	0.15835500	-0.00882700
N	0.00151100	-0.07144100	-2.17280300
C	1.19679600	0.55182000	-2.84620700
H	1.96563300	-0.21112200	-2.96775700
C	-0.02991600	-1.52300400	-2.51900400
H	0.04295200	-1.62433500	-3.61029100

H	-0.99410300	-1.92798700	-2.21215700
C	-2.58129900	0.04936100	-2.36714300
C	2.66559400	1.30729200	-0.95035800
C	4.02375200	1.63056700	-0.86867900
C	4.79407200	1.12500800	0.17549000
H	4.46420600	2.26491500	-1.63089100
C	2.83467400	0.01741600	0.98066900
C	4.19003200	0.28885900	1.11543300
H	5.84996300	1.36894400	0.24745600
H	2.31776200	-0.62083400	1.68700300
H	4.74976400	-0.14460100	1.93727600
C	0.73319700	-2.83762300	-0.46351000
C	0.89659900	-4.18614400	-0.12804700
C	-0.00608400	-2.37565700	1.68946200
C	0.59896300	-4.62543100	1.15844000
H	1.25684500	-4.87842100	-0.88194400
C	0.13741200	-3.69724900	2.09083600
H	-0.35063700	-1.62402700	2.38678400
H	0.72345500	-5.67047500	1.42645900
H	-0.10809700	-3.98120200	3.10853600
C	-3.06723200	0.00318100	-0.93810800
C	-4.45508800	-0.02514900	-0.73008300
C	-4.97183000	-0.13416300	0.55387700
H	-5.11574200	0.03618400	-1.58910900
C	-2.72125000	-0.16317900	1.34925400
C	-4.07921800	-0.20847700	1.62386100
H	-6.04512300	-0.15701500	0.71803600
H	-2.00105900	-0.19803900	2.15387200
H	-4.41969000	-0.29284900	2.65036000
N	-2.20892700	-0.06194700	0.10213800
N	2.08377100	0.52201100	-0.01677900
N	0.27994800	-1.94344400	0.44420600
C	-1.21030000	0.62859000	-2.70781300
H	-1.14663800	0.62836300	-3.80426700
H	-1.14792600	1.66610900	-2.37512000
H	-2.68192000	-0.95958800	-2.78579300
H	-3.29638500	0.65602000	-2.93421100
C	1.07687700	-2.34999700	-1.84617000
H	2.01259300	-1.78255400	-1.78923700
H	1.28252500	-3.21763700	-2.47871000
H	0.89597800	0.85732200	-3.85441600
C	1.82149300	1.74730100	-2.11779000
H	1.04736400	2.44899100	-1.79783200
H	2.45820900	2.27650000	-2.83281700
C	-0.90227600	3.40160800	0.12251500
C	-1.39097500	4.75295700	0.33626500
H	-0.84276100	5.44819400	-0.30766700

H	-2.45786200	4.79929400	0.09535700
H	-1.24315800	5.03489200	1.38374700
N	-0.51053400	2.32734200	-0.05372400
C	0.33677100	1.22894300	3.20791100
C	0.61079800	1.86487400	4.48468800
H	0.32778300	2.92121900	4.43551000
H	0.03529300	1.37073100	5.27410900
H	1.67922900	1.78492800	4.71023000
N	0.12241400	0.72232700	2.18980500

^{t}28

E (SMD/B3LYP-D3/BS1) = -1279.9672598 au
G (SMD/B3LYP-D3/BS1) = -1279.600008 au
E (SMD/B3LYP-D3/BS2//SMD/B3LYP-D3/BS1) = -2617.81237385 au
charge = 0 spin multiplicity = 3

Ni	-0.48944900	-0.07853800	-0.89315600
N	0.25609300	-0.68535400	1.00719200
C	1.73002100	-0.46143000	1.02588500
H	1.89782700	0.60243900	0.85656600
C	-0.38106300	0.04522500	2.13262000
H	0.01756700	-0.33705400	3.08552200
H	-1.44996700	-0.17241900	2.10797500
C	-1.46209300	-2.56220300	1.37987300
C	3.90308200	-0.66567600	-0.20077500
C	5.04106200	-1.41474000	0.12688300
C	6.30434700	-0.84441400	-0.03089100
H	4.93134300	-2.42953300	0.49806700
C	5.21147200	1.13694800	-0.80780000
C	6.39701100	0.46264300	-0.50985000
H	7.19865800	-1.41011400	0.21681100
H	5.24150100	2.15827800	-1.18441900
H	7.35682300	0.95034700	-0.65026100
C	-1.05232200	2.31250400	1.09548900
C	-1.59716500	3.56046500	1.42740300
C	-1.93303800	2.48318900	-1.04499200
C	-2.32644500	4.27672900	0.48353900
H	-1.44192100	3.95718300	2.42594500
C	-2.49675500	3.72613600	-0.78757700
H	-2.02851500	2.02057900	-2.02173800
H	-2.75225800	5.24423900	0.73371700
H	-3.05060500	4.24219400	-1.56489100
C	-2.53931900	-2.14047500	0.40287400
C	-3.76930400	-2.81582200	0.44280900
C	-4.80467900	-2.43127800	-0.39982400
H	-3.89763900	-3.63684700	1.14193600
C	-3.34334800	-0.75629300	-1.27552600
C	-4.58737100	-1.37040500	-1.28258100

H	-5.75932800	-2.94875000	-0.37370000
H	-3.12454300	0.05992100	-1.95473500
H	-5.35781800	-1.02901500	-1.96610900
N	-2.33341900	-1.11874000	-0.45381800
N	3.99353200	0.59864300	-0.66124000
N	-1.23196900	1.77777800	-0.13197300
C	-0.01509700	-2.14705300	1.11284400
H	0.59723800	-2.56894600	1.92575200
H	0.31609300	-2.60212100	0.17691000
H	-1.77307200	-2.24745300	2.38372700
H	-1.45875600	-3.65826600	1.41785700
C	-0.21895800	1.56842800	2.11210100
H	0.83155200	1.85246200	1.97998600
H	-0.49674100	1.93069000	3.10745200
H	2.12285900	-0.71478700	2.02470300
C	2.50881800	-1.22879100	-0.05497400
H	1.96967200	-1.10610700	-1.00724700
H	2.56940000	-2.29438500	0.18483300
O	0.38030200	-0.25898800	-2.36782700

$^{qnt}TS_{20}{}^{i}$

E (SMD/B3LYP-D3/BS1) = -2792.47354458 au
G (SMD/B3LYP-D3/BS1) = -2791.594533 au
E (SMD/B3LYP-D3/BS2//SMD/B3LYP-D3/BS1) = -5468.23697435 au
negative eigenvalue of the frequency: 1682i
charge = 3 spin multiplicity = 5

Ni	-1.39833300	-0.66876200	-0.54160300
N	-2.14807200	-1.85614700	-2.23105800
C	-1.85214400	-3.32592900	-2.17303700
H	-2.69210500	-3.83538600	-1.70323600
C	-3.62013200	-1.68253000	-2.40978300
H	-3.93770100	-2.26979700	-3.28296600
H	-3.82239000	-0.63471900	-2.62723900
C	-1.85135000	0.07265800	-3.92955100
C	-0.79733500	-3.63066700	0.08749700
C	-0.79423900	-4.78648400	0.87559500
C	-1.05388400	-4.69419100	2.23996000
H	-0.58899600	-5.74457800	0.40961500
C	-1.27793900	-2.33264900	1.95009600
C	-1.29482000	-3.43625700	2.79266700
H	-1.05607100	-5.58477800	2.86136100
H	-1.43970100	-1.33610100	2.33655600
H	-1.48567800	-3.30424000	3.85232900
C	-4.50873600	-1.12400700	-0.04515200
C	-5.73531000	-0.86416100	0.58156600
C	-3.42828800	0.19316600	1.52235900
C	-5.79138000	-0.05362800	1.71075100

H	-6.63727400	-1.31144200	0.17679100
C	-4.60192800	0.48194100	2.20272100
H	-2.48882900	0.59039500	1.87454700
H	-6.74063300	0.14946500	2.19777400
H	-4.57147100	1.11189500	3.08535000
C	-2.12973000	1.21613300	-2.98151900
C	-2.56932400	2.42207400	-3.55070600
C	-2.87434900	3.50938000	-2.74382000
H	-2.66884300	2.48512500	-4.62995000
C	-2.29793700	2.15007400	-0.87108800
C	-2.73428200	3.36950700	-1.36169200
H	-3.21209700	4.44437600	-3.18060300
H	-2.17940300	2.00764000	0.19015700
H	-2.95118200	4.18013000	-0.67462400
N	-1.99701700	1.08728700	-1.64606500
N	-1.04809900	-2.41897900	0.62663000
N	-3.36281800	-0.57499900	0.41673300
C	-1.41538300	-1.29974200	-3.41528100
H	-1.53676600	-1.99068700	-4.25985800
H	-0.35677400	-1.28438800	-3.16324300
H	-2.74736400	-0.04307700	-4.55047700
H	-1.06584100	0.39865100	-4.62355900
C	-4.46985500	-2.08773400	-1.20329900
H	-4.16448800	-3.06584600	-0.81300600
H	-5.49337100	-2.22254400	-1.56538600
H	-1.78474000	-3.70709600	-3.19885300
C	-0.58656100	-3.70389300	-1.40031500
H	0.25263900	-3.07506700	-1.70577600
H	-0.31769700	-4.72808300	-1.67330600
O	0.56983900	-0.43830800	-1.27555500
Ni	1.69296400	0.26240900	0.07545700
N	2.86635000	0.90174700	1.68741200
C	2.77772700	-0.00455600	2.91879400
H	3.74941700	-0.48266800	3.04808500
C	4.30656100	0.99859200	1.28714900
H	4.87454000	1.25582900	2.18879300
H	4.40689400	1.82319800	0.58306600
C	2.60255700	3.45211500	1.21594500
C	2.00727700	-2.18480200	1.97463800
C	2.24204500	-3.49660500	2.39689400
C	2.62666700	-4.46049700	1.46926700
H	2.12446100	-3.74447600	3.44631400
C	2.52166300	-2.76590600	-0.21175300
C	2.77665800	-4.08645100	0.13350500
H	2.80929800	-5.48390800	1.78273500
H	2.64137300	-2.43940900	-1.23861000
H	3.08079300	-4.79495500	-0.62909800

C	4.55884800	-0.31133200	-0.81897700
C	5.54256400	-0.66119800	-1.74856400
C	3.04906900	0.08192700	-2.54489300
C	5.25990900	-0.63528000	-3.10933100
H	6.52620200	-0.94022700	-1.38600500
C	3.98685500	-0.23797100	-3.51552600
H	2.04534400	0.35805500	-2.82425200
H	6.01930000	-0.90623300	-3.83631300
H	3.71022200	-0.17928300	-4.56250200
C	1.88257100	3.46699400	-0.10586300
C	1.70004700	4.70952700	-0.73225500
C	1.08135100	4.78323500	-1.97258600
H	2.05280700	5.60515800	-0.23055600
C	0.83172800	2.40950400	-1.87715800
C	0.63775700	3.59882000	-2.56220700
H	0.94089000	5.74080300	-2.46526300
H	0.47334000	1.47305100	-2.27360800
H	0.13426700	3.58910200	-3.52153200
N	1.44976700	2.32706500	-0.67890700
N	2.13761400	-1.83424400	0.67964000
N	3.31489300	0.03725600	-1.22088700
C	2.43460600	2.26638800	2.15301000
H	3.03100800	2.49668700	3.04336100
H	1.40225500	2.17197500	2.46882400
H	3.66369900	3.65664700	1.03123300
H	2.24837200	4.31445000	1.79347700
C	4.88439100	-0.25654100	0.64505700
H	4.54860400	-1.16652700	1.15111500
H	5.97127400	-0.22352900	0.75713700
H	2.62384200	0.64738500	3.77986300
C	1.69068000	-1.07792600	2.93436700
H	0.72711400	-0.63017200	2.69175800
H	1.64549300	-1.47358400	3.95321100
O	-0.01951800	0.30941900	0.94072700
H	0.96902700	-1.27890500	-1.55640700
H	-0.33734100	1.33255000	1.49163700
C	-1.43607000	4.48282000	2.83128600
C	-1.72478100	4.07273500	4.13661300
C	-1.52955700	2.74285700	4.52283100
C	-1.04328300	1.80752700	3.59859500
C	-0.78000100	2.25040500	2.30996800
C	-0.95242300	3.56147600	1.89114400
H	-1.58411700	5.51808800	2.53525300
H	-2.10089300	4.79368800	4.85712000
H	-1.75029300	2.42780000	5.53926900
H	-0.88037100	0.77127100	3.88086100
H	-0.72366600	3.87091000	0.87735400

$^{qnt}TS_{20}{}^{ii}$

E (SMD/B3LYP-D3/BS1) = -2792.47927157 au
G (SMD/B3LYP-D3/BS1) = -2791.593445 au
E (SMD/B3LYP-D3/BS2//SMD/B3LYP-D3/BS1) = -5468.23568668 au
negative eigenvalue of the frequency: 436i
charge = 3 spin multiplicity = 5

Ni	2.05172100	0.08423200	-0.05866700
N	4.08763100	0.73728500	-0.26982200
C	5.01785700	0.26412700	0.79983500
H	5.32462600	-0.75365600	0.56424500
C	4.64281700	0.34232300	-1.59919100
H	5.63849000	0.79534800	-1.70838000
H	4.00163600	0.76222000	-2.37252300
C	3.41849400	2.97341000	-1.38370300
C	3.56687700	-0.94184300	2.48518000
C	3.81171600	-1.76646500	3.58883900
C	3.05155700	-2.91706400	3.78038900
H	4.60595900	-1.50353500	4.27992400
C	1.87510600	-2.36181400	1.77677400
C	2.06658500	-3.22979400	2.84477700
H	3.23573500	-3.56355200	4.63333800
H	1.12676500	-2.57808400	1.02582100
H	1.45537400	-4.12085000	2.93079900
C	3.50105400	-1.95508900	-2.05101400
C	3.55527500	-3.10002000	-2.86080700
C	1.26723200	-2.38064000	-1.59371100
C	2.43300700	-3.90412300	-3.01380000
H	4.48778400	-3.34866900	-3.35783300
C	1.25590100	-3.53610900	-2.36055200
H	0.37626800	-2.04718700	-1.07624500
H	2.47264300	-4.79623600	-3.63187100
H	0.34936100	-4.12534700	-2.43985600
C	2.15673600	2.49781000	-2.07093200
C	1.67398100	3.26663100	-3.14027900
C	0.61682800	2.80171300	-3.91213000
H	2.15707400	4.21192600	-3.36794600
C	0.54746400	0.88245400	-2.49599800
C	0.05327200	1.56420300	-3.59682900
H	0.24361700	3.38675600	-4.74737100
H	0.10494800	-0.05261600	-2.18013000
H	-0.77028000	1.14805200	-4.16643700
N	1.57010000	1.33057700	-1.73969500
N	2.58692900	-1.23286200	1.59949500
N	2.35855500	-1.60274000	-1.42503700
C	3.99109000	2.22837600	-0.17572500
H	4.99676700	2.63870700	-0.00948900

H	3.40492800	2.45051200	0.71185300
H	4.18747600	3.03475000	-2.16341200
H	3.26324300	4.00908800	-1.05683200
C	4.76911400	-1.15773600	-1.86087800
H	5.36591200	-1.65289500	-1.08648100
H	5.36083700	-1.25634500	-2.77719900
H	5.91921900	0.89092200	0.77431100
C	4.44122600	0.25546900	2.21713700
H	3.90112900	1.18041100	2.42585500
H	5.28223900	0.23662900	2.91615900
O	0.79169100	1.25451100	1.24580200
O	-0.05472100	-0.22060200	0.30854000
H	-0.03427700	-0.67835300	1.16538400
Ni	-2.05672900	-0.44971100	-0.33090300
N	-3.64649500	-1.11335400	-1.56593000
C	-3.69344600	-2.60111400	-1.74000200
H	-4.29018100	-3.03425000	-0.93819600
C	-4.94144500	-0.63246200	-1.00363500
H	-5.76394400	-1.02552200	-1.61574000
H	-4.96219500	0.45468600	-1.07295000
C	-3.64579300	0.94705000	-3.10397600
C	-1.86749600	-3.49053700	-0.30814700
C	-1.59523100	-4.76897600	0.18391000
C	-1.33728700	-4.94324100	1.54212600
H	-1.60625300	-5.61372800	-0.49687600
C	-1.62525100	-2.58512900	1.82046500
C	-1.37304700	-3.83026000	2.38280400
H	-1.12681500	-5.93153200	1.93972900
H	-1.64819700	-1.69471000	2.43886100
H	-1.19661300	-3.91442400	3.44929500
C	-4.45336900	-0.17571000	1.49403400
C	-5.12559500	0.21132700	2.65890900
C	-2.49639200	0.83134400	2.27501900
C	-4.44736700	0.90236100	3.65742000
H	-6.17237600	-0.04928300	2.77553800
C	-3.10088700	1.21062700	3.46546800
H	-1.45144000	1.05240600	2.07879200
H	-4.95872600	1.19063400	4.57112500
H	-2.51955700	1.72149400	4.22175200
C	-2.89984800	1.92589800	-2.23592700
C	-2.98502800	3.28181500	-2.57912300
C	-2.25074400	4.22407200	-1.87530400
H	-3.61402600	3.57026700	-3.41570400
C	-1.40693400	2.42779700	-0.52966700
C	-1.43075000	3.78010400	-0.83734900
H	-2.29846300	5.27626800	-2.14023000
H	-0.74466700	2.04405500	0.23945300

H	-0.81035300	4.46594200	-0.27610000
N	-2.13899500	1.50628500	-1.20279600
N	-1.84983900	-2.40980100	0.50393400
N	-3.15366400	0.14953500	1.31138500
C	-3.36152100	-0.53263500	-2.92031300
H	-3.96266100	-1.06785300	-3.66529400
H	-2.30887800	-0.73652600	-3.13992400
H	-4.72050600	1.14951000	-3.03409100
H	-3.38322100	1.17255600	-4.14526400
C	-5.16674300	-1.01817900	0.46409100
H	-4.89755000	-2.06623200	0.64287800
H	-6.24115400	-0.94914700	0.65715900
H	-4.21095700	-2.82401600	-2.67969900
C	-2.30390800	-3.25153100	-1.72706800
H	-1.58302200	-2.61733800	-2.25482400
H	-2.34443600	-4.20305600	-2.26309300
C	-0.35860300	3.33027800	4.03520600
C	0.62061400	2.41088700	3.70117300
C	1.42739800	2.60985000	2.54763300
C	1.24715300	3.78300100	1.77471500
C	0.25069100	4.69572000	2.10878500
C	-0.54922200	4.47033700	3.23293800
H	-0.97212100	3.19156800	4.91989600
H	0.78689700	1.52898300	4.31272500
H	2.36122400	2.07067300	2.50198800
H	1.88206600	3.96721300	0.91608700
H	0.10576800	5.59042500	1.51114000
H	-1.31902600	5.18950100	3.49899100

benzene
E (SMD/B3LYP-D3/BS1) = -232.257007836 au
G (SMD/B3LYP-D3/BS1) = -232.183727 au
E (SMD/B3LYP-D3/BS2//SMD/B3LYP-D3/BS1) = -232.350339115 au
charge = 0 spin multiplicity = 1

C	0.74225100	-1.18545000	0.00003300
C	1.39775000	0.05008300	-0.00006000
C	0.65551900	1.23550300	0.00003800
C	-0.74224900	1.18545100	0.00001000
C	-1.39776100	-0.05007900	0.00000800
C	-0.65550700	-1.23550800	0.00001500
H	1.31927800	-2.10680600	0.00012300
H	2.48422200	0.08899600	-0.00020300
H	1.16514200	2.19579100	0.00012400
H	-1.31928000	2.10680700	-0.00008100
H	-2.48422000	-0.08900400	-0.00015500
H	-1.16516100	-2.19578400	-0.00007600

phenol
E (SMD/B3LYP-D3/BS1) = -307.477820852 au
G (SMD/B3LYP-D3/BS1) = -307.401897 au
E (SMD/B3LYP-D3/BS2//SMD/B3LYP-D3/BS1) = -307.611661688 au
charge = 0 spin multiplicity = 1

C	-1.85920400	0.02992900	-0.00005300
C	-1.17525200	-1.19114200	0.00009400
C	0.21927000	-1.22562700	-0.00001600
C	0.94307300	-0.02538800	-0.00008000
C	0.26744500	1.20230100	-0.00004100
C	-1.12966400	1.22204100	0.00009900
H	-2.94529500	0.05091500	-0.00032100
H	-1.73034000	-2.12584900	0.00011800
H	0.76119000	-2.16691600	-0.00003800
H	0.83445400	2.13085200	-0.00007400
H	-1.64673400	2.17827100	0.00005700
O	2.30590600	-0.11527900	-0.00002100
H	2.68546900	0.78227400	0.00041200

H_2O_2
E (SMD/B3LYP-D3/BS1) = -151.540993689 au
G (SMD/B3LYP-D3/BS1) = -151.537271 au
E (SMD/B3LYP-D3/BS2//SMD/B3LYP-D3/BS1) = -151.629374358 au
charge = 0 spin multiplicity = 1

O	0.71843700	0.10839300	-0.06527500
O	-0.71843400	-0.10838900	-0.06528100
H	-1.00984300	0.61689500	0.52223400
H	1.00981600	-0.61692500	0.52221300

H_2O
E (SMD/B3LYP-D3/BS1) = -76.4149480395 au
G (SMD/B3LYP-D3/BS1) = -76.411728 au
E (SMD/B3LYP-D3/BS2//SMD/B3LYP-D3/BS1) = -76.4712360487 au
charge = 0 spin multiplicity = 1

O	0.00000000	0.00000000	0.12081600
H	0.00000000	0.76098900	-0.48326200
H	0.00000000	-0.76098900	-0.48326200

$(H_2O)_3$
E (SMD/B3LYP-D3/BS1) = -229.272485845 au
G (SMD/B3LYP-D3/BS1) = -229.231070 au
E (SMD/B3LYP-D3/BS2//SMD/B3LYP-D3/BS1) = -229.429865365 au
charge = 0 spin multiplicity = 1

O	1.24194500	-1.26080800	0.00988300
H	1.29676300	-0.28809100	-0.08024300
H	0.27814500	-1.37907500	0.06270700

O	0.58013600	1.51536500	-0.10599700
H	-0.28343000	1.04659000	-0.10372400
H	0.69760300	1.78011000	0.82262100
O	-1.53779700	-0.31680200	0.00840100
H	-2.14392700	-0.25678200	0.76797900
H	-2.11942600	-0.40479800	-0.76763200

MeCN
E (SMD/B3LYP-D3/BS1) = -132.761905656 au
G (SMD/B3LYP-D3/BS1) = -132.740401 au
E (SMD/B3LYP-D3/BS2//SMD/B3LYP-D3/BS1) = -132.821565301 au
charge = 0 spin multiplicity = 1

C	-0.27799700	0.00007400	0.00021500
C	1.18002400	0.00000100	-0.00005000
H	1.55386600	-0.46140000	0.91980100
H	1.55363500	-0.56615300	-0.85953100
H	1.55387600	1.02732800	-0.06057400
N	-1.43907800	-0.00003200	-0.00009800

Et_3N
phenol
E (SMD/B3LYP-D3/BS1) = -292.43269277 au
G (SMD/B3LYP-D3/BS1) = -292.259695 au
E (SMD/B3LYP-D3/BS2//SMD/B3LYP-D3/BS1) = -292.550773363 au
charge = 0 spin multiplicity = 1

N	-0.17136500	0.01754600	-0.37641800
C	0.26747200	1.12496900	0.48353400
H	-0.59830200	1.77525300	0.64709000
H	0.57629200	0.76958200	1.48533600
C	0.89651900	-0.93575300	-0.71509300
H	0.46542800	-1.66577900	-1.41185400
H	1.66992700	-0.39942500	-1.27654100
C	-1.36694500	-0.64800400	0.16120100
H	-1.47121200	-1.60876200	-0.35680000
H	-1.25937700	-0.88414400	1.23667100
C	1.55400900	-1.68605300	0.45478500
H	0.81773300	-2.25955100	1.03001600
H	2.30366600	-2.39016800	0.07305900
H	2.06222600	-1.00089500	1.14301400
C	1.39036100	1.96605700	-0.12478500
H	1.55488700	2.86239400	0.48471300
H	2.34000300	1.42204000	-0.17259500
H	1.12712900	2.28479600	-1.14071400
C	-2.64897500	0.16044700	-0.05500000
H	-3.51869800	-0.41402900	0.28672900
H	-2.64274500	1.10627700	0.49797500
H	-2.78204900	0.38961700	-1.11902900

Et_3NH^+
E (SMD/B3LYP-D3/BS1) = -292.91518812 au
G (SMD/B3LYP-D3/BS1) = -292.725131 au
E (SMD/B3LYP-D3/BS2//SMD/B3LYP-D3/BS1) = -293.029539206 au
charge = 1 spin multiplicity = 1

N	0.16986300	0.00782900	0.39825700
C	-0.28324800	1.16034100	-0.47659900
H	0.56889900	1.83702700	-0.54501600
H	-0.47426700	0.74413800	-1.46719000
C	-0.92459800	-0.99853900	0.71146000
H	-0.46446400	-1.72809000	1.38150300
H	-1.68694500	-0.45875200	1.27461400
C	1.41997100	-0.66058000	-0.14440600
H	1.54582900	-1.57896200	0.43272500
H	1.21104500	-0.92481800	-1.18230300
C	-1.51280300	-1.66927100	-0.51932600
H	-0.75629100	-2.21011900	-1.09558600
H	-2.25884000	-2.39527800	-0.17973300
H	-2.01712300	-0.95485800	-1.17684600
C	-1.49486100	1.88966100	0.08300500
H	-1.65485600	2.79301400	-0.51454500
H	-2.40594500	1.28699700	0.03148900
H	-1.32928200	2.19635500	1.12232400
C	2.65533800	0.21961400	-0.02100200
H	3.52655100	-0.37106500	-0.32279600
H	2.61086600	1.10198600	-0.66522000
H	2.80823800	0.54261000	1.01539400
H	0.42874800	0.41765600	1.30460100
